# Metatranscriptome of human lung microbial communities in a cohort of mechanically ventilated COVID-19 Omicron patients

**DOI:** 10.1038/s41392-023-01684-1

**Published:** 2023-11-10

**Authors:** Lin Wang, Jia-Bao Cao, Bin-Bin Xia, Yue-Juan Li, Xuan Zhang, Guo-Xin Mo, Rui-Juan Wang, Si-Qi Guo, Yu-Qing Zhang, Kun Xiao, Guang-Fa Zhu, Peng-Fei Liu, Li-Cheng Song, Xi-Hui Ma, Ping-Chao Xiang, Jiang Wang, Yu-Hong Liu, Fei Xie, Xu-Dong Zhang, Xiang-Xin Li, Wan-Lu Sun, Yan Cao, Kai-Fei Wang, Wen-Hui Zhang, Wei-Chao Zhao, Peng Yan, Ji-Chao Chen, Yu-Wei Yang, Zhong-Kuo Yu, Jing-Si Tang, Li Xiao, Jie-Min Zhou, Li-Xin Xie, Jun Wang

**Affiliations:** 1 College of Pulmonary & Critical Care Medicine, 8th Medical Center of Chinese PLA General Hospital, Beijing, 100091 China; 2 CAS Key Laboratory of Pathogenic Microbiology and Immunology, Institute of Microbiology, Chinese Academy of Sciences, Beijing, 100101 China; 3 University of Chinese Academy of Sciences, Beijing, 100049 China; 4 Faculty of Biological Science and Technology, Baotou Teacher’s College, Baotou, 014030 China; 5 Department of Respiratory Medicine, PLA Strategic Support Force Medical Center, Beijing, 100101 China; 6 Department of Respiratory and Critical Care Medicine, Beijing Anzhen Hospital, Capital Medical University, Beijing Institute of Heart, Lung and Blood Vessel Diseases, Beijing, 100029 China; 7 Respiratory Research Institute, Department of Pulmonary & Critical Care Medicine, Beijing Key Laboratory of OTIR, the 8th Medical Center of Chinese PLA General Hospital, Beijing, 100091 China; 8 Shougang hospital of Peking University, Beijing, 100144 China; 9 Department of Respiratory Medicine, Beijing Changping Hospital, Beijing, 102200 China; 10 Department of Respiratory and Critical Care Medicine, Beijing Institute of Respiratory Medicine and Beijing Chao-Yang Hospital, Capital Medical University, Beijing, 100020 China; 11 Pulmonary research institute, Senior Department of Respiratory and Critical Care Medicine, the 8th medical center of Chinese PLA general hospital, Beijing, 100091 China; 12 College of Pulmonary & Critical Care Medicine, Chinese PLA General Hospital, Beijing, 100853 China; 13 China Aerospace Science & Industry Corporation 731 hospital, Beijing, 100074 China; 14 Department of Respiratory and Critical Care Medicine, Aerospace Center Hospital, Beijing, 100049 China; 15 Vision Medicals Center for Infectious Diseases, Guangzhou, 510700 China

**Keywords:** Infectious diseases, Infectious diseases

## Abstract

The Omicron variant of the severe acute respiratory syndrome coronavirus 2 (SARS‑CoV‑2) infected a substantial proportion of Chinese population, and understanding the factors underlying the severity of the disease and fatality is valuable for future prevention and clinical treatment. We recruited 64 patients with invasive ventilation for COVID-19 and performed metatranscriptomic sequencing to profile host transcriptomic profiles, plus viral, bacterial, and fungal content, as well as virulence factors and examined their relationships to 28-day mortality were examined. In addition, the bronchoalveolar lavage fluid (BALF) samples from invasive ventilated hospital/community-acquired pneumonia patients (HAP/CAP) sampled in 2019 were included for comparison. Genomic analysis revealed that all Omicron strains belong to BA.5 and BF.7 sub-lineages, with no difference in 28-day mortality between them. Compared to HAP/CAP cohort, invasive ventilated COVID-19 patients have distinct host transcriptomic and microbial signatures in the lower respiratory tract; and in the COVID-19 non-survivors, we found significantly lower gene expressions in pathways related viral processes and positive regulation of protein localization to plasma membrane, higher abundance of opportunistic pathogens including bacterial *Alloprevotella, Caulobacter*, *Escherichia-Shigella*, *Ralstonia* and fungal *Aspergillus sydowii* and *Penicillium rubens*. Correlational analysis further revealed significant associations between host immune responses and microbial compositions, besides synergy within viral, bacterial, and fungal pathogens. Our study presents the relationships of lower respiratory tract microbiome and transcriptome in invasive ventilated COVID-19 patients, providing the basis for future clinical treatment and reduction of fatality.

## Introduction

The coronavirus disease 2019 (COVID-19) pandemic has caused over 6.8 million deaths worldwide since the beginning of 2020.^1^ The most recent variant, Omicron, has the highest transmissibility (R_0_ > 7) of all variants to date.^2^ In December 2022, China relaxed the national “zero COVID” policy; and by January 2023, it was estimated that ~80% of individuals in China had been infected by the Omicron variant, and the general population may have established herd immunity. Despite the decreased fatality and lower proportion of severe cases caused by Omicron compared to previous variants (such as Beta and Delta), the large number of infections still led to a large number of hospitalizations and deaths. Official reports state that the current wave has caused over 1.6 million in-patient hospitalizations and ~60,000 COVID-related deaths in China.^3^ Thus far, several parameters have been linked to increased COVID-19 severity and fatality rates, namely patient age (older), sex (male), and the presence of underlying diseases (especially diabetes, hypertension, and those causing immune suppression).^4^ However, additional contributing factors remain identification which may aid in establishing disease prognoses and developing axillary therapies.

The microbiome of the lower respiratory tract has increasingly been recognized for its importance in both infectious and non-infectious respiratory diseases, refuting an earlier hypothesis that the lung is a sterile environment.^5^ In chronic obstructive pulmonary disease (COPD), the third most common cause of death worldwide, exacerbation of the disease is associated with increases in pathogenic bacteria such as *Proteobacteria* and *Moraxella* in the commensal microbiome^6^; host immune system stimulation by such microbes increases inflammation.^7^ In bacterial, fungal, and viral pneumonia, co-infections commonly occur with opportunistic pathogens present in the commensal microbial community.

Microbiome-based analyses of sputum and fecal samples collected from individuals with COVID-19 have revealed significant shifts in the microbial communities compared to healthy controls,^8,9^ and additional bacterial signatures have been associated with disease severity.^10^ However, the microbiome of the lower respiratory tract (which theoretically has closer interactions with COVID-19, a pneumonic disease) has not been thoroughly characterized; as in severe and fatal cases, the disease manifestation is most prominent in the lower respiratory tract, and the lower respiratory tract microbiome would be the most relevant.

We hypothesized that the eventual clinical outcomes (survival or death) in invasive ventilated cases of COVID-19 were associated with the lung microbiome, and that co-infections and shifts in the commensal microbiome could be identified from analysis of the BALF. Additionally, host gene transcripts from the BALF would provide information regarding differences in immune responses and other potential responses to microbiome changes. To assess this hypothesis, we carried out transcriptomic analysis using both the Oxford Nanopore Technology (mtTGS)^11^ and Illumina (mtNGS) platforms. Analyses were conducted on BALF samples collected within a short period between the end of 2022 and the beginning of 2023 from a Beijing cohort of invasively ventilated COVID-19 patients who required intubated ventilation and had not been exposed to previous variants of severe acute respiratory syndrome coronavirus (SARS-CoV)-2.

## Results

### Cohort description

Between December 7th, 2022, and January 25th, 2023, we collected BALF and blood samples from a total of 64 individuals who were intubated due to severe pneumonia and tested positive for SARS-CoV-2. The individuals were sampled from among eight medical centers in the Beijing area of China. The median age of the probands was 80.00 (70.00–85.00), and 48 (75.00%) were male. Among the 64 probands, a total of 45 died within 28 d of intubation (non-survivors) and the remaining 19 were discharged after recovery (survivors). Samples were also collected from an additional cohort of severe Hospital Acquired Pneumonia/Community-Acquired Pneumonia (HAP/CAP) patients from November to December 2019 (before the COVID-19 pandemic), consisting of 20 survivors and seven non-survivors; there were no significant differences in the age or gender distributions compared to the COVID-19 patient cohort (Supplementary Table. 1).

### Clinical parameters associated with 28-d mortality

We first investigated anthropometric characteristics, clinical parameters, and cytokine profiles associated with clinical outcomes in invasive ventilated COVID-19 patients. We found no significant differences between survivors and non-survivors with respect to age distribution (Mann–Whitney *U*-test, one-tailed *p* = 0.49), age, or blood parameters (Table 1); however, the survivors had significantly lower levels of SOFA (11.00, 9.50–14.00 in non-survivors compared to 8.00, 7.00–11.00 in survivors; *p* < 0.01), lower APACHE II scores (26.00, 24.00–27.00 in non-survivors and 19.00, 17.00–21.00 in survivors; *p* < 0.01), and higher levels of PaO_2_/FiO_2_ (132.86, 86.65–231.00 in non-survivors and 188.00, 145.00–344.00 in survivors; *p* < 0.05) at the time of intubation (Table 1). Cytokine levels in the BALF and plasma indicated that the BALF of non-survivors had significantly higher levels of interleukin (IL)-17 (4.39, 3.53–6.42 in non-survivors and 3.53, 2.73–5.47 in survivors; one-tailed *p* < 0.05), IL-6 (254.58, 21.09–782.93 in non-survivors and 21.16, 10.75–305.34 in survivors; one-tailed *p* < 0.05), IL-10 (4.66, 2.25–9.83 in non-survivors and 2.17, 1.71–4.13 in survivors; one-tailed *p* < 0.01), and TNF-α (22.14, 11.83–58.82 in non-survivors and 9.24, 4.68–27.76 in survivors; one-tailed *p* < 0.01), and significantly lower levels of IL-5 (1.81, 1.20–3.76 in non-survivors and 2.86, 1.84–4.07 in survivors; one-tailed *p* < 0.05) (Table 1). We also investigated the percentage of co-morbidities in non-survivors and survivors, but found no significant differences with respect to diabetes, hypertension, or respiratory system diseases.

**Table 1:** Clinical Characteristics of the Study Population

	Survival (*n* = 19) Median (IQR)	Death (*n* = 45) Median (IQR)	*P* value
Age, yr	80.00 (16.00)	80.00 (13.00)	0.49
Sex			0.22
Male, *n* (%)	16.00 (84.21)	32.00 (75.00)	
Female, *n* (%)	3.00 (15.79)	13.00 (25.00)	
Co-morbidity^a^, *n* (%)	16.00 (84.21)	42.00 (93.33)	0.25
SOFA score	8.00 (4.00)	11.00 (5.00)	<0.01
APACHE II score	19.00 (4.00)	26.00 (3.00)	<0.01
PaO_2_/FiO_2_	188.00 (199.00)	132.86 (144.35)	0.02
D-dimer (μg/L)	2270.00 (5626.00)	2012.00 (3492.00)	0.45
Lactic acid(mmol/L)	1.50 (1.30)	2.00 (1.30)	0.15
BNP(pg/ml)	209.00 (615.00)	278.50 (717.00)	0.33
TnI(pg/mL)	21.89 (128.65)	13.50 (49.20)	0.70
CK-MB(ng/mL)	3.00 (5.77)	3.58 (12.70)	0.19
ALT(U/L)	21.00 (54.40)	22.80 (25.20)	0.92
AST(U/L)	29.90 (42.90)	36.05 (24.70)	0.71
Total bilirubin (μmol/L)	12.90 (9.00)	13.60 (9.55)	0.49
Albumin (g/L)	30.60 (7.80)	30.10 (7.35)	0.06
Creatinine (μmol/L)	88.00 (67.55)	81.50 (89.44)	0.38
BUN(mmol/L)	11.33 (9.16)	11.90 (11.08)	0.73
UA(μmol/L)	262.30 (184.80)	292.80 (171.80)	0.55
GLU(mmol/L)	8.12 (5.24)	9.93 (8.05)	0.51
Neutrophil(10^9^/L)	7.75 (6.36)	10.12 (8.40)	0.21
Neutrophil percentage(%)	91.00 (6.30)	92.40 (10.00)	0.17
Lymphocyte (10^9^/L)	0.51 (0.40)	0.45 (0.37)	0.19
Lymphocyte percentage (%)	5.00 (4.20)	4.20 (5.10)	0.16
Platelet (10^9^/L)	124.40 (85.00)	171.00 (138.50)	0.10
Erythrocyte(10^12^/L)	3.71 (1.58)	3.74 (1.07)	0.99
Leukocyte(10^9^/L)	8.57 (6.37)	11.60 (8.73)	0.44
Monocyte(10^9^/L)	0.39 (0.67)	0.39 (0.36)	0.93
**BALF cytokine**	**Survival (*n*** = 19)	**Death (*n*** = 45)	
IL-5(pg/mL)	3.15 (6.30)	4.95 (3.53)	0.18
IFN-α(pg/mL)	4.56 (3.27)	5.99 (4.86)	0.05
IL-2(pg/mL)	2.32 (1.96)	2.67 (2.02)	0.07
IL-6(pg/mL)	506.78 (1448.00)	610.73 (1386.36)	0.30
IL-1β(pg/mL)	277.98 (1255.93)	919.89 (3290.46)	0.05
IFN-γ(pg/mL)	12.85 (8.13)	19.61 (28.10)	0.05
IL-8(pg/mL)	7864.79 (9553.95)	8930.17 (8409.79)	0.25
IL-17(pg/mL)	3.53 (2.74)	4.39 (2.89)	0.04
IL-12P70(pg/mL)	2.73 (1.25)	2.90 (1.64)	0.15
TNF-α(pg/mL)	9.24 (23.08)	22.14 (46.99)	<0.01
IL-4(pg/mL)	2.98 (1.92)	3.00 (2.04)	0.25
IL-10(pg/mL)	3.58 (2.60)	3.53 (3.67)	0.05
**Plasma cytokine**	**Survival (*n*** = 18)	**Death (*n*** = 42)	
IL-5(pg/mL)	2.86 (2.23)	1.81 (2.56)	0.02
IFN-α(pg/mL)	1.65 (2.50)	1.25 (1.89)	0.17
IL-2(pg/mL)	1.41 (1.60)	1.25 (0.72)	0.22
IL-6(pg/mL)	21.16 (294.59)	254.58 (761.84)	0.03
IL-1β(pg/mL)	1.57 (11.70)	0.00 (4.52)	0.20
IFN-γ(pg/mL)	4.44 (7.97)	3.79 (7.97)	0.39
IL-8(pg/mL)	14.51 (15.87)	21.25 (38.41)	0.06
IL-17(pg/mL)	2.95 (1.73)	3.11 (2.33)	0.25
IL-12P70(pg/mL)	1.75 (0.35)	1.76 (0.35)	0.47
TNF-α(pg/mL)	0.41 (4.71)	0.77 (2.57)	0.46
IL-4(pg/mL)	1.46 (0.31)	1.48 (0.42)	0.49
IL-10(pg/mL)	2.17 (2.42)	4.66 (7.58)	<0.01

^a^Co-morbidity is defined as ≥1 of the following conditions: hypertension, diabetes, cardiac disease, kidney disease, digestive system disease, autoimmune disease, thma, chronic obstructive pulmonary disease, and malignancy. *SOFA* sequential organ failure assessment, *APACHE II* Acute Physiology and Chronic Health Evaluation II, *PaO*_*2*_ */FiO*_*2*_ partial pressure of oxygen (PaO_2_)/fraction of inspired oxygen (FiO_2_), *BNP* brain natriuretic peptide, *TnI* troponin I, *CK-MB* Creatine Kinase MB, *BUN* blood urea nitrogen, *UA* blood uric acid, *GLU* glucose, *IQR* interquartile range

### SARS-CoV-2 genome analysis

**Fig. 1:**
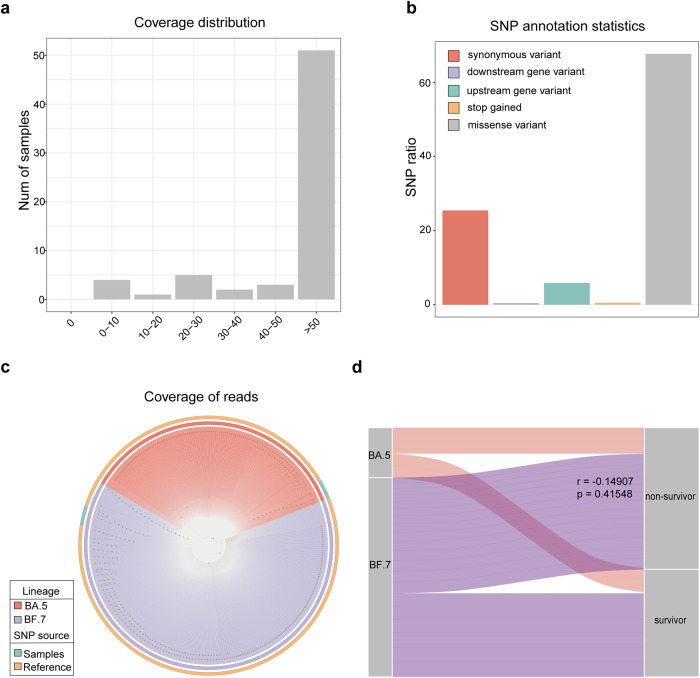
Analysis of SARS-CoV-2 genomes in the BALF samples. **a** Coverage distribution of reads mapped to reference SARS-CoV-2 genome, in which we found 8 complete genomes and 43 > 50% completeness. COVID-19 patients (*n* = 63). **b** Overview of SNP variant types in the assembled genome, in total we found 34 insertions, 18 deletions, and 583 substitutions. COVID-19 patients (*n* = 51). **c** Phylogenetic relationships of the 51 genomes assembled in this study vs. 682 SARS-CoV-2 genomes sampled in Beijing general population from the same period and deposited in GISAID. 20 genomes belong to BA.5 and 24 to BF.7 lineages. COVID-19 patients (*n* = 44). **d** The correlation between SARS-CoV-2 genomes of BF.7 and BA.5 lineages and 28-day mortality (survivors or non-survivors) based on Spearman’s rank test, no significant differences were found between the 28-day mortality rate in the two strains. COVID-19 patients (*n* = 32)

Using long reads generated from the Oxford Nanopore Technology (ONT) platform and short reads from Illumina sequencing (Fig. 1a), we assembled and examined the genomes of the SARS-CoV-2 strains infecting our cohort. A total of 27 complete or near-complete and five partial genomes were assembled from the 64 patients (Supplementary Table. 2). Phylogenetic analysis indicated that each genome belonged to one of the two SARS-CoV-2 lineages that dominated this wave of Omicron infections, BA.5 and BF.7 (Fig. 1b, c). We examined the distribution of the BA.5 and BF.7 lineages between survivors and non-survivors, and found no significant difference in the survival rate between those infected with the two lineages; two survivors and 10 non-survivors were infected with BA.5, whereas six survivors and 14 non-survivors were infected with BF.7 (*p* = 0.41, Spearman’s rank sum test) (Fig. 1d). Single nucleotide polymorphism (SNP) analysis revealed a total of 319 and 243 SNPs in the BA.5 and BF.7 lineages, respectively. A recent genomic analysis of Beijing Omicron strains incorporated 682 genomes assembled primarily from the general population of COVID-19 patients in Beijing during the same time period as our cohort collection; 131 of the SNPs we identified here were also reported in that analysis (Supplementary Table. 3). There was no significant enrichment of individual SNPs in our cohort of invasive ventilated COVID-19 patients compared to the percentages among the 682 genomes (*p* > 0.05, Fisher’s exact test for all SNPs), nor were there significant differences in particular SNPs between the survivor and non-survivor groups (*p* > 0.05, Fisher’s exact test for all SNPs). Thus, it was not likely that particular SARS-CoV-2 Omicron lineages or individual SNPs were responsible for a higher occurrence of invasively ventilated COVID-19 cases or increased mortality.

### Host transcriptomic signatures in survivors and non-survivors of COVID-19

Human transcripts accounted for the majority of the BALF metatranscriptomic data, averaging 56.47% of the reads. These transcripts were used to profile host gene expression in the lung tissue of invasive ventilated COVID-19 patients and to identify signatures specific to COVID-19 patients through comparison to data from severe HAP/CAP patients before the pandemic. First, in comparing gene expression in the invasive ventilated COVID-19 patients compared to the severe HAP/CAP patients, we found a total of 2,324 significantly up-regulated genes (|fold change [FC]| > 1.5, *p* < 0.05) and 33,668 down-regulated genes (|FC | > 1.5, *p* < 0.05) (Supplementary Fig. S1a). Pathway enrichment analysis of the differentially expressed genes suggested that the intrinsic apoptotic signaling and response to virus pathways were significantly elevated in COVID-19 compared to HAP/CAP patients, reflecting a distinct host response to SARS-CoV-2 infections (Supplementary Fig. S1b).

**Fig. 2:**
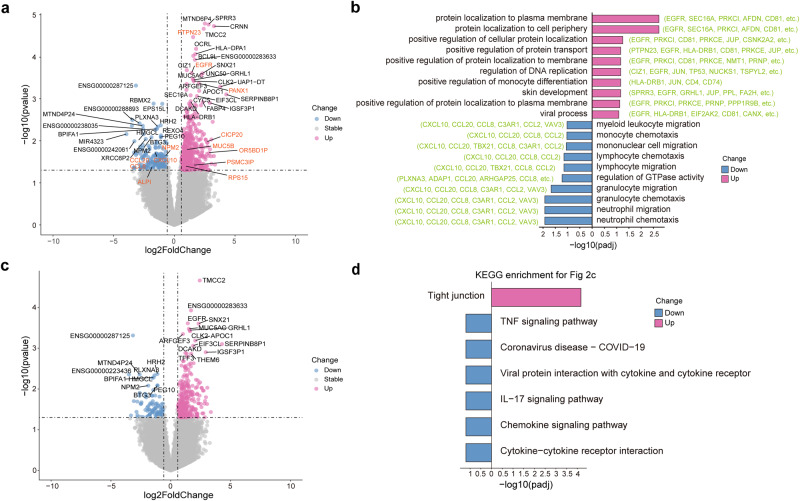
Gene Differential Expression Analysis in BALF of COVID-19 Patients. **a** Volcano plot comparing gene expression between the survival and non-survival groups of COVID-19 patients. 489 genes were up-regulated and 187 genes were down-regulated in the survival group (abs(FC) > 1.5, *p*-value < 0.05), including red-highlighted genes that were functionally characterized or implicated in subsequent analyses. **b** Gene Ontology (GO) enrichment analysis (Biological Process) of the differentially expressed genes identified in (**a**), where the representative genes involved in each GO term are indicated in parentheses. **c** Volcano plot displaying genes with differential expression between COVID-19 patients and HAP/CAP patients, which are also differentially expressed in the survival group of COVID-19 patients. These genes are specific to COVID-19 survival patients and include 268 up-regulated and 97 down-regulated genes. **d** KEGG enrichment analysis of the differentially expressed genes identified in (**c**). HAP/CAP patients (*n* = 27), COVID-19 patients (*n* = 63), non-survivors of the invasive ventilated COVID-19 patients (*n* = 45), survivors of the invasive ventilated COVID-19 patients (*n* = 18)

Next, we compared gene expression between invasive ventilated COVID-19 survivors and non-survivors. In survivors, there were 489 significantly up-regulated genes, with the most strongly up-regulated genes including *EGFR*, *PTPN23*, and *PANX1*. There were 187 significantly up-regulated genes in non-survivors, the most strongly up-regulated of which included *CXCL10*, *CCL20*, and *CCL8* (Fig. 2a). Pathway enrichment analysis indicated that genes associated with viral processes were significantly up-regulated in survivors. Neutrophil chemotaxis and neutrophil migration could be observed to be significantly enriched in non-survivors (Fig. 2b). Further, we compared the intersection of external differential genes (compared to HAP/CAP) with internal differential genes (COVID-19 survivors compared to non-survivors), and we identified 268 and 97 significantly up-regulated genes in survivors and non-survivors in COVID-19 patients, respectively (Fig. 2c). These genes demonstrate both up- and down-regulated genes in COVID-19 survivors, but exclude the portion shared by HAP/CAP that is specific to COVID-19 patients. Among the overlapping differential KEGG pathways in COVID-19 patients vs HAP/CAP patients, and COVID-19 Survivors vs. Non-survivors. Cytokine-cytokine receptor interaction, Chemokine signaling pathway, and IL − 17 signaling pathway were significantly enriched in non-survivors (Fig. 2d), agreeing with previous reports on COVID-19 patients.^12^ Further examination of the intersection of internal differential genes in COVID-19 patients and internal differential genes in HAP/CAP patients, eight of which were shared with survivors and non-survivors of invasive ventilated COVID-19: *IGHA1*, *CACNA1C-AS3* upregulated in survivors of both cohorts, and *BPIFA1*, *RIT1*, *CCDC146*, *CFAP300*, *ENSG00000290744*, *WTAP* upregulated in non-survivors of both cohorts (Supplementary Fig. S1c). The gene *BPIFA1* encodes a product with antimicrobial activity^13^ and was significantly down-regulated in survivors of COVID-19 patients with bacterial co-infections, suggesting attenuated host responses as a result of co-infection. *IGHA1*, which is associated with an antibody immune response, was significantly up-regulated in survivors, suggesting an increased immune response in COVID-19 patients. These genes were significantly associated with clinical outcomes in both COVID-19 and HAP/CAP patients. In addition, COVID-19 non-survivors showed significant enrichment of genes associated with neutrophil chemotaxis and lymphocyte migration compared to the HAP/CAP non-survivors, indicating that these factors may specifically explain clinical outcomes in those infected with COVID-19 (Fig. 1d).

### The lower respiratory tract microbiome and COVID-19 outcome

**Fig. 3:**
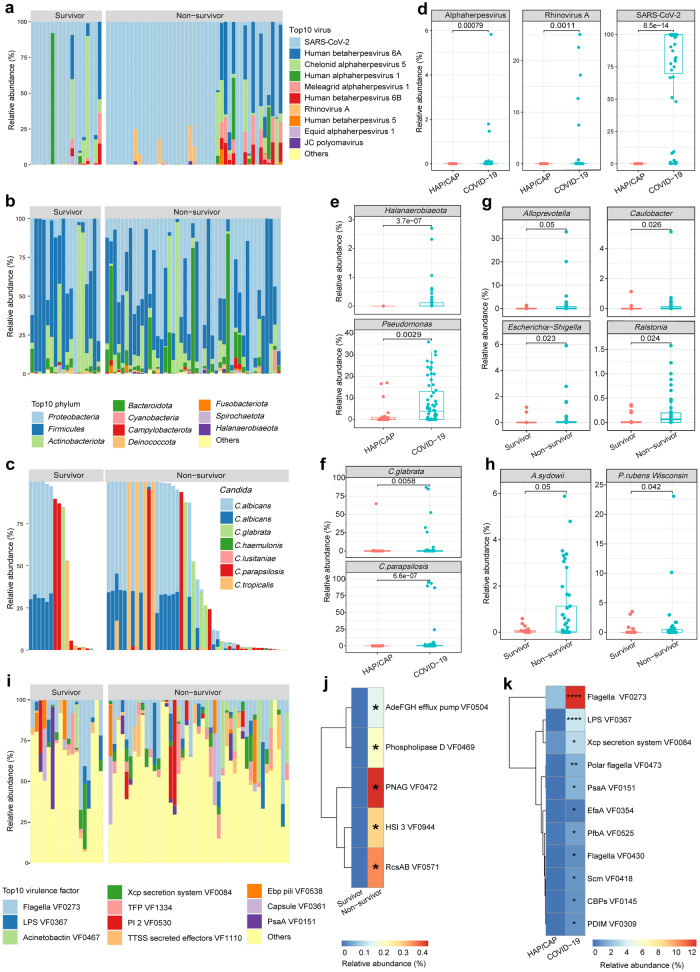
Alternation of microbiome and virulence factors in COVID-19 and HAP/CAP patients. **a** Composition and relative abundance of the top 10 viruses in invasive ventilated COVID-19 patients. **b** Relative abundance of the top ten bacterial phylum levels in invasive ventilated COVID-19 patients. **c** Relative abundance of fungi belonging to the genus *Candida* in COVID-19 patients. **d** Box plot of differential virus between invasive ventilated COVID-19 and HAP/CAP patients, significance was derived from Wilcoxon tests. **e** Box plot of differential bacteria between invasive ventilated COVID-19 and HAP/CAP patients, significance was derived from Wilcoxon tests. **f** Box plot of significantly different fungi between invasive ventilated COVID-19 and HAP/CAP patients. **g** Box plot of significantly different bacterium between survivors and non-survivors of the invasive ventilated COVID-19 patients. **h** Box plot of significantly different fungi between survivors and non-survivors of the invasive ventilated COVID-19 patients. **i** Relative abundance of the top ten virulence factors in invasive ventilated COVID-19 patients. **j** Heatmap of significantly different virulence factors between survivors and non-survivors of invasive ventilated COVID-19 patients. **k** Heatmap of differential virulence factors between invasive ventilated COVID-19 and HAP/CAP patients. HAP/CAP patients (*n* = 27), COVID-19 patients (*n* = 63), non-survivors of the invasive ventilated COVID-19 patients (*n* = 45), survivors of the invasive ventilated COVID-19 patients (*n* = 18). One-tailed wilcoxon rank-sum test was used for all significance statistics, *p-value < 0.05, **p-value < 0.01, ***p-value < 0.001, ****p-value < 0.0001

We next analyzed the lung microbial community composition of each patient by mapping metatranscriptomic data to reference genomes of viral, bacterial, and fungal species. Comparative to negative controls, BALF samples of our cohorts exhibited significant differences in microbial compositions in each comparison, in addition to significantly higher concentrations of nucleic acid after extraction and library construction (Supplementary Fig. S2; Supplementary Table. 4). Of the fragments that could not be mapped to the human transcriptome (i.e., microbial reads), sequences from the virome accounted for an average of 34.82% of the total microbial reads; these were primarily from SARS-CoV-2 (averaging 75.97% of the viral reads) and Human betaherpesvirus (averaging 8.89% of the viral reads) (Fig. 3a). Bacterial reads accounted for 40.52% of all microbial reads, with the most abundant phyla being *Proteobacteria, Firmicutes, Actinobacteriota*, and *Bacteroidota* (averaging 39.49%, 38.31%, 12.60%, and 7.64% of the bacterial reads, respectively). At the genus level, the most abundant taxa included *Acinetobacter, Pseudomonas*, and *Klebsiella* (averaging 12.14%, 9.48%, and 1.76% of the bacterial reads, respectively), all of which belong to the phylum *Proteobacteria* and are pathogens that have previously been reported in co-infections with COVID-19 (Fig. 3b). Importantly, fungal reads were found in all of the invasive ventilated COVID-19 individuals and accounted for an average of 24.65% of the microbial reads, with most of these mapping to *Candida* species (an average of 51.11% of fungal reads) (Fig. 3c).

Compared to the severe HAP/CAP patients from 2019, our cohort of invasive ventilated COVID-19 patients had significantly different microbial signatures. First, invasive ventilated COVID-19 patients showed significant enrichment of SARS-CoV-2 (99.74%, 0–100 in invasive ventilated COVID-19 patients compared to 0 in HAP/CAP patients; *p* = 8.5e-14) *Rhinovirus A* (0%, 0–25.09 compared to 0; *p* = 0.0011) and Alphaherpes virus (0%, 0–5.82 compare to 0; *p* = 0.00079) among the viral reads **(**Fig. 3d**)**. In the bacteriome, invasive ventilated COVID-19 patients were significantly enriched in *Halanaerobiaeota* at the phylum level (0.006%, 0–2.70 compared to 0%, 0–0.0051 in HAP/CAP patients; *p* = 3.7e-07) and *Pseudomonas* at the genus level (4.85%, 0.0026–42.12 compared to 0.46%, 0–56.55 in HAP/CAP patients; *p* = 0.04) **(**Fig. 3e**)**. Several fungal species were also significantly enriched in invasive ventilated COVID-19 patients, such as *Candida glabrata* (0%, 0–86.95 compared to 0%, 0–64.66 in HAP/CAP patients; *p* = 5.7e-03) and *Candida parapsilosis* (0.0049%, 0–95.07 compared to 0%, 0–0.44 in HAP/CAP patients; *p* = 6.6e-07) (Fig. 3f).

In contrast, we found no significant differences in the percentages of SARS-CoV-2 reads out of all viral reads between invasive ventilated COVID-19 survivors and non-survivors (99.75%, 0.05–99.96 and 99.52%, 0–100, respectively; *p* = 0.48) (Supplementary Fig. S3), or other viruses. However, among bacterial reads, there were significant differences in the percentages of *Alloprevotella* (0.0006%, 0–1.41 in survivors and 0.023%, 0–32.86 in non-survivors; *p* < 0.05)*, Caulobacter* (0.0002%, 0–1.14 in survivors and 0.0046%, 0–5.12 in non-survivors; *p* = 0.026)*, Escherichia-Shigella* (0.000018%, 0–1.17 in survivors and 0.0016%, 0–5.91 in non-survivors; *p* = 0.023) and *Ralstonia* (0.0011%, 0–0.37 in survivors and 0.064%, 0–1.58 in non-survivors; *p* = 0.024) (Fig. 3g). Among non-survivors, the fungal reads showed significant enrichment of *Aspergillus sydowii* (0.029%, 0–0.56 in survivors and 0.048%, 0–5.88 in non-survivors; *p* = 0.05) and *Penicillium rubens Wisconsin* (0.00019%, 0–3.51 in survivors and 0.042%, 0–23.09 in non-survivors; *p* = 0.04). These enriched species indicated likely synergistic effects between bacterial, fungal, and viral pathogens that contributed to worse outcomes (Fig. 3h). Sputum culture results for a number of patients in the COVID-19 cohort were indeed positive for *Escherichia* or *Aspergillus* (Supplementary table. 5). Among the KEGG pathways enriched in COVID-19 Survivors compared to HAP/CAP Survivors (Supplementary Fig. S1d) and COVID-19 Non-survivors compared to HAP/CAP Non-survivor (Supplementary Fig. S1e), Coronavirus disease -- COVID-19 pathways and Pathogenic *Escherichia coli* infection and *Salmonella* infection pathways were both enriched, indicating the importance of co-infection of bacteria, especially *Escherichia*/ *Salmonella* with SARS-CoV-2 is signature of the COVID-19 cohort non-survivors and play important role in shaping the clinical outcome. We also found significant differences between severe HAP/CAP survivors and non-survivors in the abundance of *Cardiobacterium* (0%, 0–3.39 in survivors and 0.00028%, 0–0.0032 in non-survivors; *p* < 0.05), *Ligilactobacillus* (0.0036%, 0–0.66 in survivors and 0.035%, 0.004–1.41 in non-survivors; *p* = 0.011), and *Lactobacillus* (0.0013%, 0–0.15 in survivors and 0.025%, 0.00094–3.59 in non-survivors; *p* = 0.0083) among the bacterial reads (Supplementary Fig. S4); in the mycobiome, *Aspergillus aculeatus* (0%, 0–1.94 in survivors and 0.023%, 0–0.88 in non-survivors; *p* = 0.0014) and *Aspergillus flavus* (0%, 0–0.6 in survivors and 0%, 0–11.08 in non-survivors; *p* = 0.0091) were significantly enriched in non-survivors (Supplementary Fig. S5).

We also examined the toxicity factors that may have contributed to clinical outcomes. Among invasive ventilated COVID-19 patients, the most common toxicity factors included VF0273 (flagella), which was present in 92% of patients, VF0084 (Xcp secretion system), which was present in 65% of patients, and VF0467 (acinetobactin), which was present in 60% of patients (Fig. 3i). Among invasive ventilated COVID-19 patients compared to severe HAP/CAP patients in 2019, there was significant enrichment (one-sided Wilcoxon test, *p* < 0.05) of factors involved in motility (VF0237 [flagella], VF0430 [flagella], and VF0473 [polar flagella]); immune modulation (VF0367 [LPS] and VF0309 [PDIM]); adherence (VF0525 [PfbA], VF0418 [Scm], VF0145 [CBPs], and VF0354 [EfaA]); nutritional/metabolic factors (VF0151 [PsaA]), and the effector delivery system (VF0084 [Xcp secretion system]) (Fig. 3k). Toxicity factors that were significantly enriched in non-survivors of COVID-19 cohort included VF0504 (AdeFGH efflux pump) (0 in survivors and 0%, 0–2.85 in non-survivors; *p* = 0.04), VF0944 (HSI-3) (0 in survivors and 0%, 0–4.27 in non-survivors; *p* = 0.04), VF0469 (phospholipase D) (0 in survivors and 0%, 0–2.91 in non-survivors; *p* = 0.03), VF0472 (PNAG) (0 in survivors and 0%, 0–11.09 in non-survivors; *p* = 0.03), and VF0571 (RcsAB) (0 in survivors and 0%, 0–10.38 in non-survivors; *p* = 0.04) (Fig. 3j). We found also significant positive correlation between cytokine levels in the BALF and blood with the common toxicity factors. In the BALF, the strongest positive correlation was between IL-1β and VF0504 (AdeFGH efflux pump) and VF0470 (Phospholipase C). In the blood, all the cytokine levels except IL-6 were positively correlated with six toxicity factors including VF0368 (BvrR-BvrS), VF0003 (Capsule), VF0560 (Capsule), VF1138 (Curli fibers), VF0228 (Enterobactin) and VF0521 (ESX-3) (Supplementary Fig. S6). There were also significant differences between severe HAP/CAP survivors and non-survivors, however, none overlapped with the differential virulence factors we found in the COVID-19 cohort (Supplementary Fig. S7).

### Correlational analysis of lung microbiome, host transcriptome, and immune responses

**Fig. 4:**
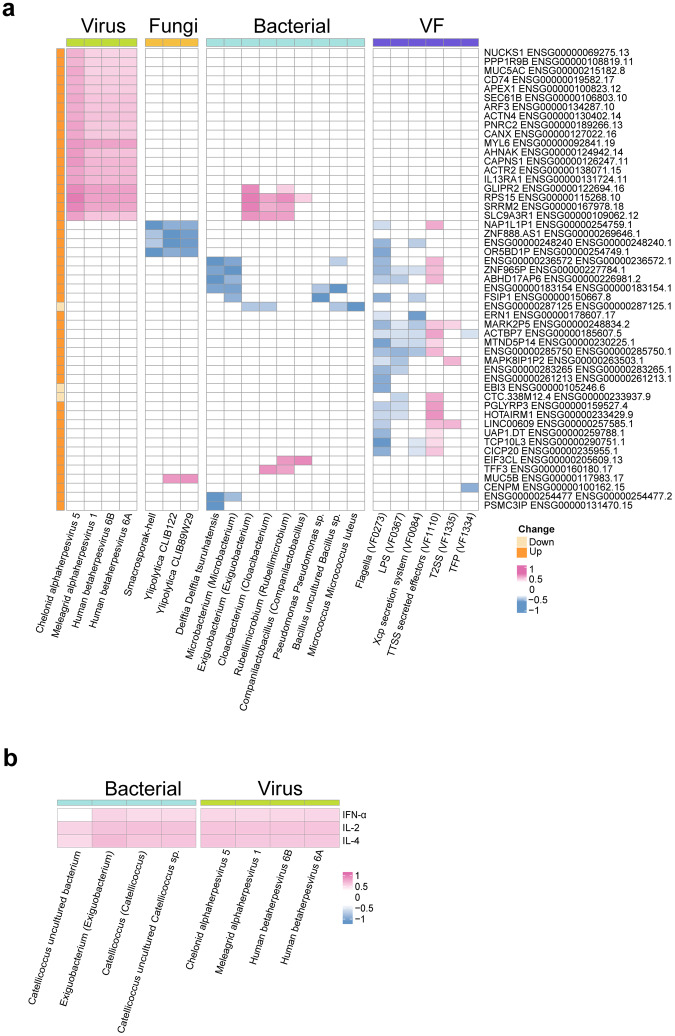
Multi-omics correlation analysis of COVID-19 patients. **a** Heatmaps display the correlation analysis of viral, fungal, bacterial, and virulence factor, with differentially expressed genes in COVID-19 patients. Spearman correlation coefficients with adjusted *p*-values < 0.05 are marked in colored cells. **b** Heatmaps depicting correlation analysis between differentially expressed genes of viral and bacterial with cytokine levels in invasive ventilated COVID-19 patients. Positions with adjusted *p*-values < 0.05 are shown in color code, and the Spearman correlation coefficient numbers are within each grid. Only absolute correlation coefficients greater than 0.5 are displayed. COVID-19 patients (*n* = 63)

Last, we probed potential interactions between the microbiome and invasively ventilated COVID-19 host responses by analyzing correlational networks between and among omics datasets (Fig. 4). The virome had the largest number of significant correlations with host gene expression (76 pairs with |r | > 0.5) and with cytokine expression (12 pairs with |r | > 0.5). The most highly correlated pairs were Chelonid alphaherpesvirus 5 and *RPS15* (r = 0.66), Chelonid alphaherpesvirus 5 and *SRRM2* (r = 0.72). Chelonid alphaherpesvirus 5 and IL-4 were significantly positively correlated (r = 0.60). Chelonid alphaherpesvirus 5 and Human betaherpesvirus 6 A were showed positive correlations with IL-2 (both r = 0.59). The abundance levels of specific bacteria were positively correlated with the differential expression of 14 genes and three cytokines. Notably, the strongest negative correlation (r = -0.70) was between the genus *Delftia* and *PSMC3IP* expression levels, and the strongest positive correlation was between the genus *Exiguobacterium* and *RPS15* expression (r = 0.66). Three fungal taxa were correlated with differential host gene expression, including correlations between *Sordaria macrosporak-hell* and OR5BD1P, *Y. lipolytica* CLIB89W29 and *MUC5B* (r = -0.62 and r = 0.603, respectively) (Fig. 4a). Positive correlations between bacteria and cytokines included *Exiguobacterium* and IL-4 (r = 0.64), *Catellicoccus* and IL-2 (r = 0.61), and an uncultured *Catellicoccus* strain and IL-2 (r = 0.60) (Fig. 4b). Potential synergistic effects were also observed within the microbiome, such as a significant correlation between flagella (VF0273) and 23 bacterial genera; the strongest correlation was with *Allorhizobium-Neorhizobium-Pararhizobium-Rhizobium* (r = 0.68). Notably, there were also strong positive correlations between flagella (VF0273) and the fungal strains *Y. lipolytica* CLIB122 and *Y. lipolytica* CLIB89W29 (r = 0.65 and r = 0.66, respectively) (Supplementary Fig. S8).

## Discussion

In the present study, we profiled clinical parameters, the host transcriptome, and the lower respiratory microbial community in a cohort of invasive ventilated COVID-19 patients infected with Omicron variants. Several signatures were significantly associated with 28-d mortality. There were no significant differences between survivors and non-survivors with respect to age or gender, but SOFA levels, APACHE II scores, and PaO_2_/FiO_2_ levels were associated with mortality, as were levels of IL-17 and TNF-α in the lung and of IL-6 and IL-10 in the serum. The two major variants forming this Omicron wave in Beijing, which affected over 80% of the population within a single month, were BA.5 and BF.7; there were no significant differences between the mortality rates of those they infected. The SARS-CoV-2 viral load was not significantly different between survivors and non-survivors, but we did find significant enrichment among non-survivors of opportunistic bacterial pathogens including *Alloprevotella, Caulobacter, Escherichia-Shigella*, and *Ralstonia*, and of fungal species in the genera *Aspergillus* and *Penicillium*. These genera were thus associated with worse outcomes, which could be further examined in future clinical samples and animal experiments. Host transcriptomic analysis in the lung tissue revealed that genes related to myeloid leukocyte migration and neutrophil migration (namely *CXCL10*, *CCL20*, and *CCL8*) were significantly enriched among non-survivors. Lastly, the microbial compositions in BALF samples are significantly associated with the inflammation levels, and non-survivors did have higher signatures of inflammation, further hinting at the importance of lung microbiome. Our study thus investigated, for the first time, the lung microbiome and host immunological responses in intubated and mechanically-ventilated cases of SARS-CoV-2 Omicron infections, the current most dominant variants that would continue to infect the global populations, and we demonstrate that lower-respiratory tract microbiome is indicative of the clinical outcome and could provide new insights into the diagnosis and treatment of this important disease.

The Omicron variant of SARS-CoV-2 appeared in Oct 2021, and has been associated with a decrease in the percentages of severe and lethal cases. However, the significantly higher transmissibility of Omicron compared to the earlier Beta and Delta variants presents new challenges in public health and medicine.^14^ We here found no difference in SARS-CoV-2 viral load between survivors and non-survivors using sequencing-based or reverse transcription (RT)-PCR approaches; furthermore, there were no significant differences in mortality between the Omicron strains BA.5 and BF.7, suggesting that other patient characteristics or the microbial community structure may have been associated with mortality.^15^ Further analysis indicated that none of the SNPs were linked to increased fatality within each strain, thus at least according to our study cohort, there were no mutations of concern. However, our data could not test adaptation or transmission. The previous studies were centered around the earlier variants of SARS-CoV-2 (e.g. Alpha, Beta, and Delta strains), which had higher fatality and viral loads that were more important for clinical outcomes.^16^ While our study focused on the Omicron variants, in particular BA.5 and BF.7, which have higher transmissibility yet lower fatality, thus other factors such as lung microbiome and other host responses play a more important role in shaping the clinical outcomes. Among the cohort assessed in this study, 28-d mortality was associated with generally worse respiratory function (indicated by factors such as SOFA levels), elevated levels of IL-6 in the serum, and increased levels of IL-17 and TNF-α in the lung; these characteristics reflected a higher level of systemic and/or local inflammation, consistent with previous studies.^17^ Moreover, the host transcriptomic profiles indicated that immune globulin responses were suppressed in non-survivors at the time of mechanical ventilation, similar to findings during the earliest wave of infections.^18^ As reported in previous studies, SARS-CoV-2 induce host cell death via apoptosis, necroptosis, pyroptosis, autophagy, and PANoptosis, in turn, these are processes that can be targeted in therapies. Our study found that COVID-19 patients have upregulated pathways in apoptotic signaling pathways and response to virus/viral process, including genes *TNF, TNFAIP3, TNFSF10, TNFRSF1B, TNFRSF1*. Macrophage-produced TNF-α can directly inhibit viral replication and indirectly activate tissue-resident macrophages, dendric cells, innate NKs and neutrophils;^19^ they are upregulated in COVID-19 infections as a response to viral entry but also this induces cell apoptosis and potentially cytokine storms.^20^ Our observation confirms that the TNF-α and associated genes are the major distinct responses in COVID-19 patients compared to HAP/CAP patients, which potentially contribute to the severity of the disease and has the potential to be targeted in COVID-19 specific therapies. In addition, neutrophil migration was among the most strongly enriched pathways in non-survivors; it has previously been established that neutrophil dysregulation, particularly of neutrophil activation and neutrophil extracellular traps (NETs),^21^ can exacerbate acute respiratory distress (ARDS) and cytokine storm in COVID-19, leading to higher fatality rates among affected individuals. Intriguingly, differentially expressed genes shared between non-survivors of COVID-19 and HAP/CAP included genes associated with both innate immunity (e.g., *BPIFA1*) and adaptative immunity (e.g., *IGHA1*). In COVID-19 non-survivor genes related to neutrophil chemotaxis and migration, including *CXCL10, CCL20, CCL8, C3AR1, CCL2, VAV3* are significantly upregulated; increased neutrophil is a typical signature of COVID-19 and closely linked to fatality, as already reported by other studies. *BPIFA1* is an important component of innate immunity, the upregulation hints at co-infection of bacteria. We found that in HAP/CAP and COVID-19 cohorts, non-survivors had significantly higher levels of expression than survivors, presenting a novel marker for prognosis and also demonstrating the potential importance of bacterial infection to negative clinical outcomes. *BPIFA1* participates also in the inflammatory response of the upper respiratory tract and stimulates the migration of macrophage and neutrophils, agreeing well with the enrichment of neutrophil chemotaxis and migration process in the COVID-19 non-survivors. Additionally, we found increased expression of Histamine H2 Receptor (*HRH2*) and *BPIFA1*. Increased histamine and receptors could elevate the inflammations in the lung, and facilitate the invasion of SARS-CoV-2 into endothelial cells. Lastly in survivors upregulated genes were mainly in protein localization and transportand viral process, and the central gene *EGFR* is known to be related to fibrosis after SARS-CoV-2 infections, suggesting that it is potentially beneficial in terms of short-term clinical outcomes (promoting tissue healing and fighting off infections), yet there is a trade-off in the long term.

The impact of the host commensal microbiome on COVID-19 was first noticed very soon after the pandemic began. A series of studies focused on the microbiota of the oral cavity, upper respiratory tract, and gut because these sites could be readily sampled; they concluded that there were significant changes in microbial community structure in patients with mild to severe COVID-19 compared to healthy individuals.^10^ However, BALF samples, which would be more directly associated with this pneumonic disease, were rarely studied due to the difficulties associated with sampling. Here, metatranscriptomic analysis revealed that a distinct microbiome signature was associated with the clinical outcome (28-d mortality). Furthermore, opportunistic bacterial pathogens and fungal species that are known to cause secondary infections were significantly more abundant in non-survivors at the time of mechanical ventilation,^22^ consistent with previous studies in which the lung microbiota were analyzed post-mortem.^23,24^ In severe COVID-19 patients, a disease that is caused by a viral pathogen, we found that the clinical outcome (28-d mortality) was not associated with viral load of SARS-CoV-2 or other viruses, but a significant increase of opportunistic pathogens including *Alloprevotella*, *Caulobacter, Escherichia-Shigella*, and *Ralstonia*, and of fungal species in the genera *Aspergillus* and *Penicillium*; such sequence-based findings were also supported by sputum culturing results, especially for *Escherichia* and *Aspergillus*. Such pathogens strongly hints at secondary infections or coinfections after being infected with SARS-CoV-2, and further worsen the clinical outcome, a phenomenon that was observed in non-survivors of both COVID-19 and HAP/CAP, and repeatedly reported in those with lung infections caused by either viral or bacterial primary pathogens.^24,25^ Gram-negative pathogens encode Flagella leading to significant elevation in inflammation of the host and likely promoting the pathologies of viral pathogens.^26^ Our correlational analysis also found the typical virulence factors of Gram-negative bacteria lead to worsened outcomes and higher degrees of inflammation. Additionally, co-infections with bacterial pathogens disrupt the mucosal layer and epithelial cells of the lung, interfere with host metabolism, and could even develop into sepsis and cytokine storm.^27^ Similarly, fungal species such as *Aspergillus* are known to cause Aspergillosis by itself, while additional/pre-existing viral or bacterial infections could lead to increased Aspergillus infections. *Aspergillus* and other fungal pathogens cause persistent infections via highly diverse toxins and pro-inflammatory pathogen-associated molecular pattern molecules (PAMPs), and in severe cases can cause fungal antigenemia and also cytokine storms.^28^ In severe influenza infections the occurrences and importance of *Aspergillus* are gaining wide attentions,^29^ as well as in COVID-19.

The lower respiratory tract microbiomes of COVID-19 non-survivors also contained significantly higher abundances of virulence factors related to LPS and flagella, both of which indicated an increased presence of Gram-negative pathogens and known inflammatory factors contributing to the severity of COVID-19 and other pneumonic infections.^30^ Significant correlations between bacterial, fungal, and viral abundance in the BALF samples and cytokine levels in the BALF and/or serum samples further imply an important role of the lower respiratory tract in shaping patient immune responses^31^ and eventual mortality. In the correlational analysis, we explored the co-infected viruses/bacterial/fungi and the transcriptomes of the host, and further revealed signatures associated with clinical outcome (28-day mortality). A total of 19 differential genes between survivors and non-survivors were positively correlated with viral abundances, indicating the importance of co-infected viruses in shaping host responses and eventual outcomes. Among those genes, *MUC5AC* encodes the protective proteins for lung mucus,^32^ while CD74 is essential for the processing of MHC II antigens by stabilizing the heterodimers forming MHC II.^33^ Additionally, RPS15 encodes a protein that participates in the processing and translation of viral mRNAs.^34^ The results indicate that the progress and clinical outcome are not solely dependent on SARS-CoV-2, and other viral members influence the host immune responses as well and affect the final outcome. The same correlations were also found to bacterial and fungal abundances, as well as virulence factors. For example, bacterial components such as Flagella (VF0273), LPS (VF0367), Type IV pili (TFP, VF1334), and XCP secretion system, TTSS, T2SS are significantly correlated to host immune genes, and many parts of the lung microbiome could have contributed to the progression of the disease, affected the severity and determined the eventual outcome.

In this study, we conducted an extensive examination of patient clinical and immune parameters, lower respiratory tract microbiomes, and lower respiratory tract transcriptomes. Using transcriptome-targeted analysis and applying both Illumina sequencing platform (mtNGS) as well as long-reads sequencing of Nanopore (mtTGS),^11^ we provided deeper insights into the RNA viral genome of SARS-CoV-2, and spontaneously profiled the active microbial communities, their function and expressed pathogenic factors, and the host gene responses. Commonly used mNGS that target DNA in infection studies thus can be significantly improved by the application of mtNGS/mtTGS. However, these data can establish only correlations, not causality. Furthermore, the sample sizes and patient types (i.e., those with COVID-19 or HAP/CAP rather than healthy individuals) were limited by the difficulties presented in collecting BALF samples, and indeed correction for multiple testing would render no significant differences in terms of bacterial and fungal compositions, or transcriptomic differences between survivor and non-survivor group. After patients began treatment in the intensive care unit, antibiotic usage, and other medications may also have played roles in the mortality rate^35^; controlling for this effect will require further analysis with continual sampling. The roles of opportunistic bacterial and fungal pathogens in shaping the host immune response and mortality will also require dedicated studies in animal models in the future. In addition, there are known cases of cross-kingdom interactions between gut virome, bacteriome, and mycobiome in the context of healthy and SARS-CoV-2, but further validations of their function, as well as extension into the lung, requires further efforts.^36,37^

In summary, we here assessed the relationships between patient parameters and lower respiratory tract microbiomes with mortality resulting from invasive ventilated COVID-19. Overall, higher levels of inflammation decreased respiratory function, and increased abundance of opportunistic bacterial and/or fungal pathogens in the BALF were indicative of eventual mortality among the cohort studied here. The BALF microbiome was closely associated with patient inflammation levels, as indicated by cytokine levels, and virulence factors were significantly correlated with host transcriptional responses. Taken together, these results provide valuable insights into invasive ventilated COVID-19 prognoses based on clinical and microbial parameters and form a basis for future differential treatment targeting opportunistic pathogens and/or host immune factors.

## Materials and methods

### Study design and participants

In this study, a total of 64 patients admitted to the intensive care units of eight hospitals in Beijing from Dec 7th 2022, and Jan 25th 2023 were included. They were diagnosed with SARS-CoV-2 infection through qPCR or antigen testing and required intubated mechanical ventilation; patients younger than 18 years of age and pregnant women were excluded. All of the patients of COVID-19 infections were from emergency clinics; our clinical records indicated that no antibiotics, antivirals, or immune modulators were used before admission. All patients or their legal representatives agree to join and obtain approval from the Ethics Committee of the General Hospital of the People’s Liberation Army of China (Ethics No.: 2022113030901836). Samples of bronchoalveolar lavage fluid (BALF) are collected using a standardized bronchoscopy protocol, and stored at −80 °C until processing, time between admission and sampling was limited to a few hours. Blood samples were collected from veins and stored at 4 °C until they were processed as soon as possible. We collected 64 BALF and 60 blood samples immediately after invasive mechanical ventilation. Blood parameters including lactic acid, albumin, creatinine, and cytokine levels in blood and BALF were also collected, and the patients were evaluated using SOFA and APACHE II scores, plus PaO_2_/FiO_2_. In addition, a total of 27 BALF samples were collected from patients diagnosed with Hospital-acquired Pneumonia/Community-Acquired Pneumonia (HAP/CAP) (Supplementary Table. 6) and all were on intubated mechanical ventilation, collected from November to December 2019 in an earlier study,^11^ were also included and subjected to the same meta-transcriptomic analysis. Pathogens in the HAP/CAP cohorts were confirmed by routine clinical testing, in addition to sequencing analysis (Supplementary Table. 7).

### Cytokine measurement

The plasma and BALF cytokines were detected using FACS Canto Plus flow cytometry (BD, USA) and the multiplex bead-based flow fluorescence immunoassay kit (Qingdao, China). Prepare standard samples and test samples according to the kit instructions, and then conduct tests to obtain results.

### Meta-transcriptomic sequencing

Total RNA from BALF was extracted from BALF using Trizol, before being treated with DNase I to remove the contaminating DNA, and cDNA was obtained by reverse transcription using random primers. For Oxford Nanopore sequencing (mtTGS), library preparation was performed according to the manufacturer’s instructions for barcoding cDNA/DNA (SQK-LSK109 and EXP-FLP002). Eight samples were pooled together for one library and a total of eight libraries were constructed for sequencing, Oxford Nanopore PromethION R9 FLO-PRO002 flow cells for 48–72 h. For meta-transcriptomic sequencing on Illumina platform (mtNGS), total RNA was extracted using QIAamp UCP pathogen minikit (Qiagen, Valencia, CA, USA), The RNAs were purified, fragmented, and reverse-transcribed and enriched by PCR to construct the final cDNA libraries, one sample failed to build a library. Sequencing was performed on the NextSeq 550 System (Illumina, San Diego, CA). At least 60 million paired-end 150 base pair (bp) reads were generated for each sample. A total of four controls were included to rule out environmental contaminants or nucleotides introduced during library construction and sequencing.

### SARS-CoV-2 genomic analysis

FASTQ reads from Oxford Nanopore Technology (ONT) and Illumina platforms were mapped to the reference SARS-CoV-2 genome (GenBank accession number MN908947.3) using minimap2 v2.17-r941^38^ and STAR 2.7.10b,^39^ respectively. The mapped Illumina sequences were dereplicated by sambamba 0.6.6,^40^ and further applied to call single nucleotide polymorphism (SNP) variants by gatk 4–4.3.0.0.^41^ All SNP variants were annotated and their functional effect was predicted using SnpEff 5.1d.^42^ Assembly of the SARS-CoV-2 genome was carried out using MEGAHIT v1.2.9.^43^

The assembled genomes were compared to a total of 682 SARS-CoV-2 genomes of BF.7 and BA.5 lineages collected from Beijing, China during 2022.10.13 to 2023.1.31 from the GISAID database, and phylogenetic tree was constructed using Neighbor-Joining method using Phylip 3.697.^44^ Second, these genomes were mapped to SARS-CoV-2 reference sequences using MUMmer v3’s^45^ number alignment to call SNP.

### Host gene expression profiling

Meta-transcriptomic sequencing using Oxford Nanopore Technology (ONT) platform (mtTGS) and Illumina platform (mtNGS) was performed to obtain host transcriptomic information. The fastq files from NGS Sequencing were aligned to transcript reference GRCh38 by Hisat2 (version 2.21) to get sam files. Next, the sam files were converted to bam files by Samtools version (1.6). Next, we used featureCounts (version 2.0.1) to quantify gene expression (with parameter “-g gene_id”).

Differential expression analysis was performed with DESeq2 (version 1.34.0)^46^ by taking genes or transcripts count matrix as input. We filtered out the genes/transcripts which were not expressed in any sample. The Fold Change (FC) and p-value of each annotated gene were calculated.

Gene Ontology (GO) and Gene Set Enrichment Analysis (GSEA) were performed using version 4.2.0 of the R package clusterProfiler.^47^ The differentially expressed genes (DEGs) were subjected to GO analysis using the enrichGO function to identify enriched biological processes. To evaluate the biological significance of the gene sets, GSEA was performed using the gseGO function.

### Metatranscriptome analysis of Illumina reads

The quality-controlled illumina sequencing data were filtered out of the human reference genome (GRCH38 reference database) using KneadData (v0.10.0).^48^ Using the Virosaurus^49^ (downloaded in January 2023) viral genome dataset as a reference, the sequences after host removal were mapped and analyzed using bowtie2 (v2.3.4.1),^50^ requiring genome coverage >30% and depth >1X, then the taxonomic classification and relative abundance of viruses were calculated using the ‘samtools idxstats’ command.^51^ Meanwhile, to obtain bacterial annotation information at the species level, read counts mapping to 16 S rRNA genes in the SILVA^52^ database were calculated using the PhyloFlash (v3.4)^53^ selecting a similarity greater than or equal to 98% as the threshold value. The eukaryotic pathogen genome database EUPATHDB-46^54^ was used as a reference for qualitative and quantitative analysis of fungal pathogens using bowtie2 and samtools.

### Profiling of virulence factor in BALF of patients with invasive ventilated COVID-19

Bacterial virulence factor searches were performed on the metatranscriptome using the shortBRED toolkit (v0.9.5),^55^ using the Virulence Factor Core Dataset (VFDB)^56^ (downloaded in March 2023) as a reference. Briefly, the shortbred_identify.py script was used to identify and quantify unique markers of virulence factors.

### Statistical analysis

Continuous data are presented as a median with an interquartile range and analyzed using a Mann–Whitney test. For clinical characteristics of the study chorts, One-tailed degree of significance between groups was set at *P* < 0.05. The statistics for the significance of differences in microbial abundance were all performed using the one-tailed Wilcox test. All statistical analyses were performed in Rstudio using R4.2.0, and plotted using the ggplot2 package. Unless specified, multiple-testing correction was not carried out due to the sample size of the cohort, and mainly the non-corrected statistical tests were presented.

### Multi-omics association analysis

In conducting correlation analysis, a minimum presence of 20 samples for bacteria, fungi, viruses, virulence factors, BALF cytokines, and host differential genes is mandatory, and the features that fail to meet this criterion are omitted beforehand. We utilized the corAndPvalue function of the R package WGCNA^57^ to perform the correlation analysis, which enabled the calculation of the Spearman correlation coefficient. Benjamini & Hochberg step-down false discovery rate controlling procedure was then applied for *P*-value correction, using an alpha value of 0.05. We set the threshold for selecting the significant correlation matrix at an adjusted *p*-value < 0.05. For host correlations with bacteria, fungi, and virulence factors, we displayed only those rows and columns where the correlation coefficient was greater than 0.6, while for other correlation matrices, the minimum correlation coefficient threshold was 0.5.

### Supplementary information

Supplementary Materials-pdf

## Data Availability

Data are available in manuscript or supplementary materials. Meta - transcriptomic sequencing data are deposited to National Microbiological Data Center under the link: https://nmdc.cn/resource/genomics/project/detail/NMDC10018476.
