# Supplementary material for: Metatranscriptome of human lung microbial communities in a cohort of mechanically ventilated COVID-19 Omicron patients
Source: Signal Transduct Target Ther. 2023 Nov 10;8:432. doi: 10.1038/s41392-023-01684-1 (PMC10638395; doi:10.1038/s41392-023-01684-1)
Supplement: Supplementary file 1 — Supplementary Materials-pdf [file 41392_2023_1684_MOESM1_ESM.pdf]

Supplementary Materials for

Metatranscriptome of human lung microbial communities in a cohort of mechanically ventilated COVID-19 Omicron patients

Lin Wang#[1], Jia-Bao Cao#[2], Bin-Bin Xia#[2,3], Yue-Juan Li[2,3]#, Xuan Zhang[2,15], Guo-Xin Mo[1], Rui-Juan Wang[4], Si-Qi Guo[2], Yu-Qing Zhang[2,3], Kun Xiao[1], Guang-Fa Zhu[5], Peng-Fei Liu[1], Li-Cheng Song[1], Xi-Hui Ma[6], Ping-Chao Xiang[7], Jiang Wang[1], Yu-Hong Liu[1], Fei Xie[1], Xu-Dong Zhang[2,3], Xiang-Xin Li[8], Wan-Lu Sun[9], Yan Cao[10], Kai-Fei Wang[11], Wen-Hui Zhang[2,3], Wei-Chao Zhao[4],Peng Yan[12], Ji-Chao Chen[13], Yu-Wei Yang[6], Zhong-Kuo Yu[1], Jing-Si Tang[2], Li Xiao[6], Jie-Min Zhou[14], Li-Xin Xie$[1], Jun Wang$[2,3]

Correspondence to: junwang@im.ac.cn or xielx301@126.com

This PDF file includes:

Figures. S1 to S8
Tables S1 to S7

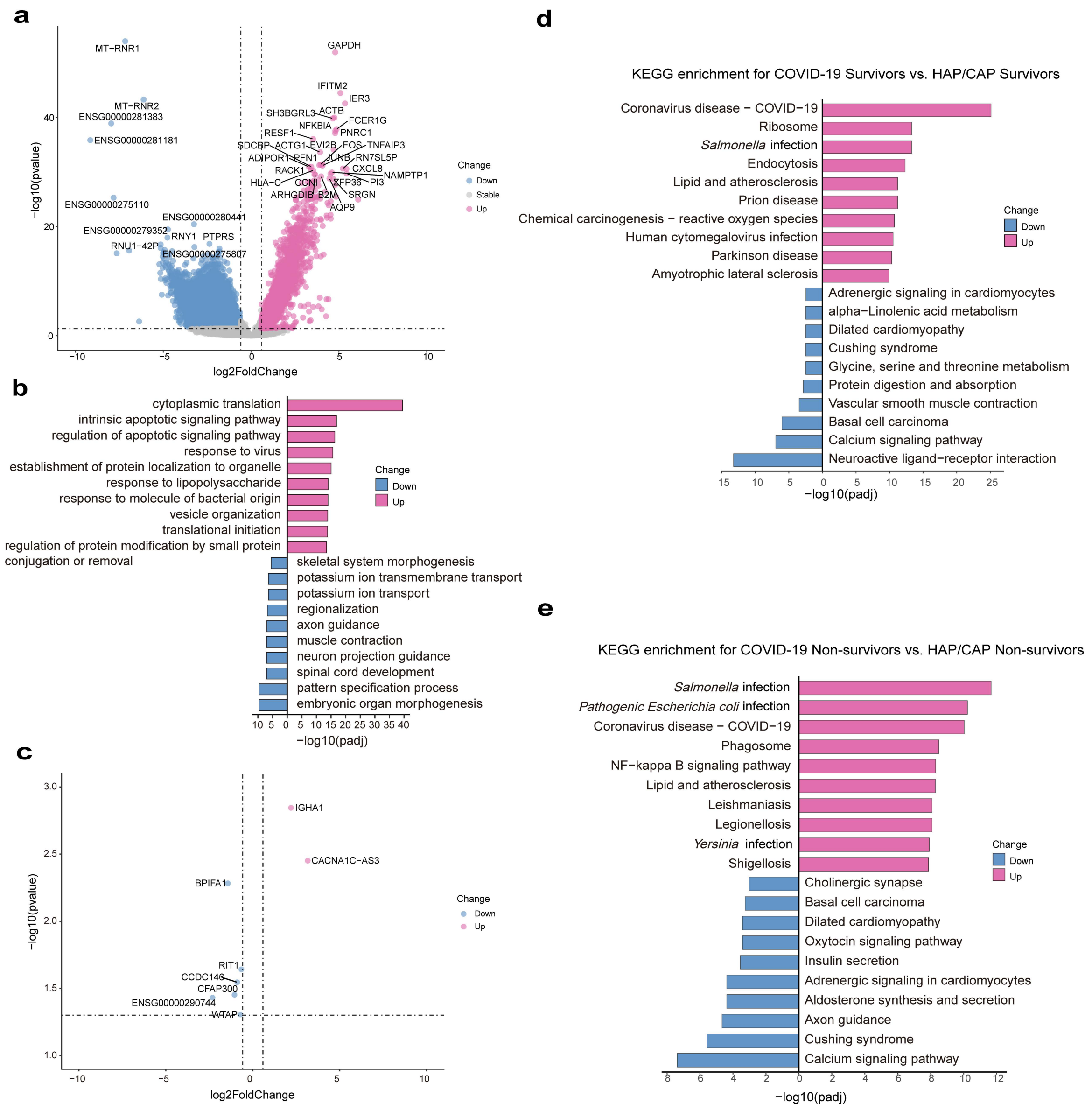

Figure. S1. Gene Differential Expression Analysis in BALF of HAP/CAP Patients.

(a) Volcano plot is presented to illustrate differential gene expression between invasive ventilated COVID-19 patients and HAP/CAP patients. The survival group exhibited up-regulation of 2324 genes and down-regulation of 33668 genes.

(b) Gene Ontology (Biological Process) enrichment analysis results for the differentially expressed genes identified in (a).

(c) The volcano plot shows the shared differentially expressed genes in HAP/CAP patients in both the survival and non-survival groups as well as the COVID-19 patient group, which survived, with matching directions of the differential expression. Down: down-regulated in survival group, Up: up-regulated in survival group.

(d) KEGG enrichment analysis for the differentially expressed genes identified in COVID-19 Survivors vs. HAP/CAP Survivors.

(e) KEGG enrichment analysis for the differentially expressed genes identified in COVID-19 Non-survivors vs. HAP/CAP Non-survivors. HAP/CAP patients (n=27), COVID-19 patients (n=63), non-survivors of the invasive ventilated COVID-19 patients (n=45), survivors of the invasive ventilated COVID-19 patients (n=18).

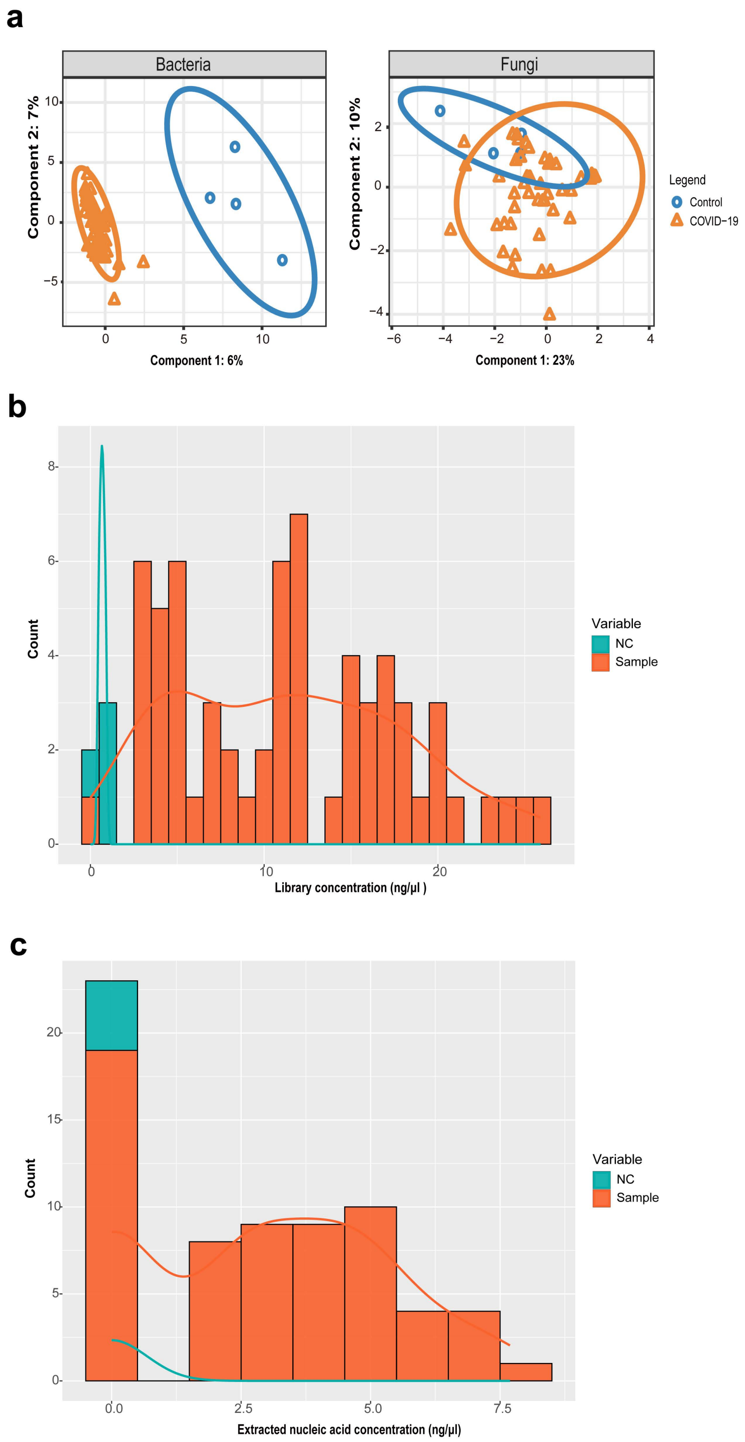

Figure. S2. Comparison of BALF samples with Negative Controls.

(a) Partial least squares discriminant analysis (PLS-DA) of COVID-19 and negative controls based on bacterial and fungal relative abundance. The left and right panels show significant differences in bacterial and fungal composition between COVID-19 and the negative reference, respectively. (b) The Library concentration of BALF samples with negative controls.

(c) The Extracted nucleic acid concentration of BALF samples and negative controls. COVID-19 patients (n=64), Negative Controls(n=4).

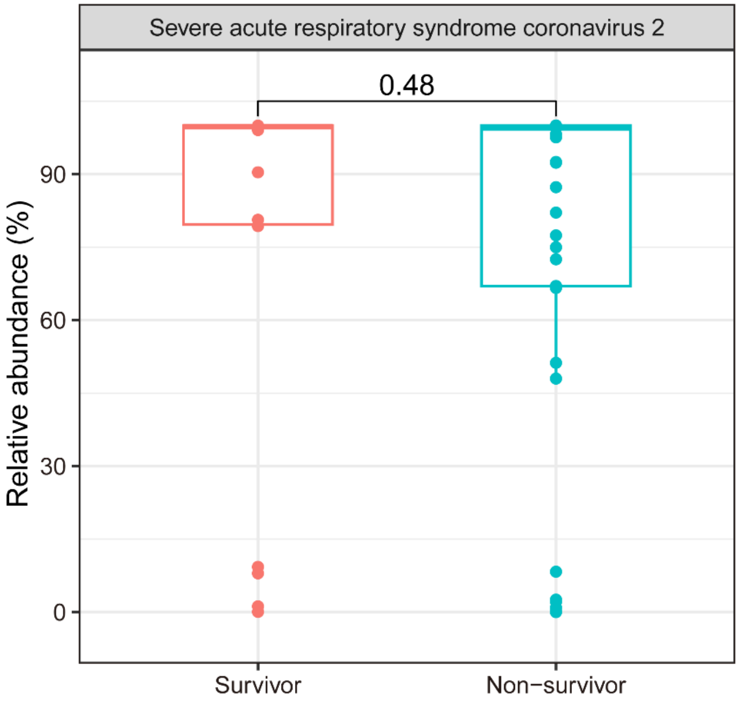

Figure. S3. Comparison of SARS-CoV-2 abundance in survivors and non-survivors of invasive ventilated COVID-19.

Box plot of SARS-CoV-2 relative abundance in survivors and non-survivors of invasive ventilated COVID-19. One-tailed wilcoxon rank-sum test was used for significance statistics. COVID-19 patients (n=63), non-survivors of the invasive ventilated COVID-19 patients (n=45), survivors of the invasive ventilated COVID-19 patients (n=18).

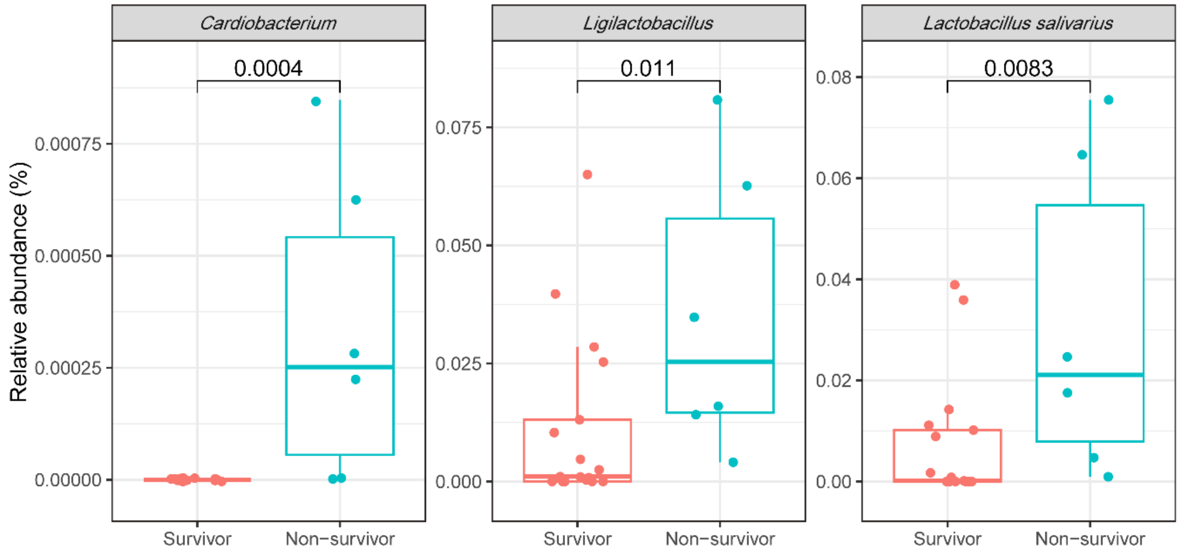

Figure. S4. Significantly different bacteria between survivors and non-survivors of HAP/CAP patients.

One-tailed Wilcoxon rank-sum test was used for significance statistics. HAP/CAP patients (n=27), non-survivors of the HAP/CAP patients (n=7), survivors of the HAP/CAP patients (n=20).

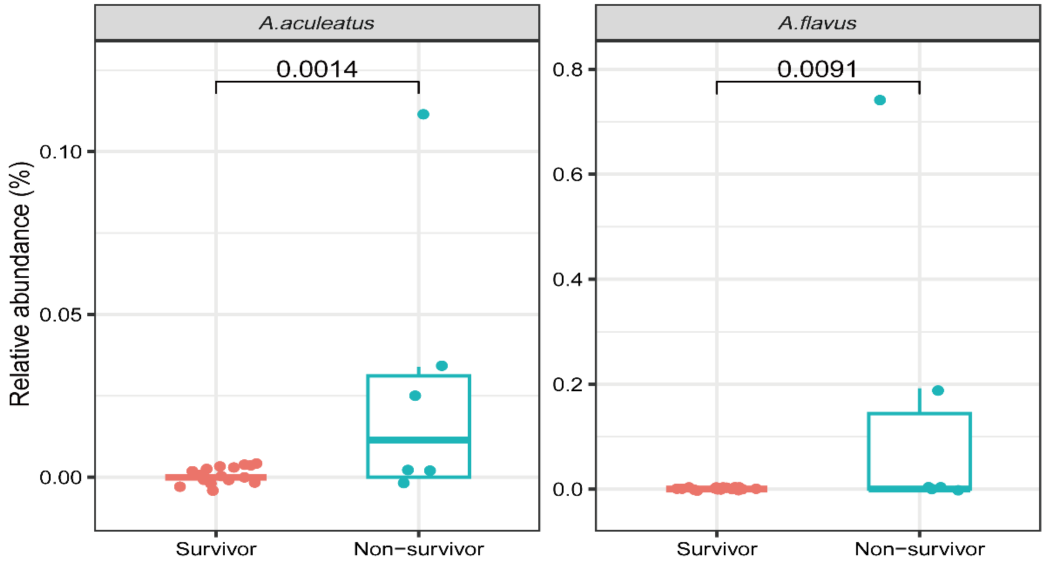

Figure. S5. Significantly different fungi between survivors and non-survivors of HAP/CAP patients.

One-tailed wilcoxon rank-sum test was used for significance statistics. HAP/CAP patients (n=27), non-survivors of the HAP/CAP patients (n=7), survivors of the HAP/CAP patients (n=20).

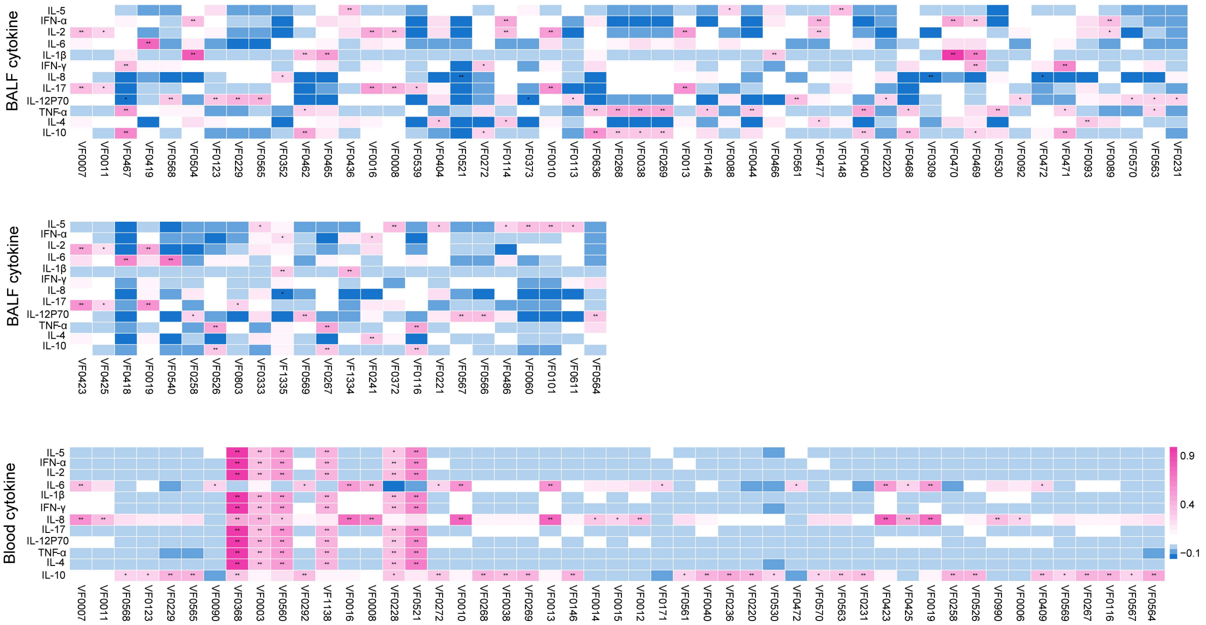

Figure. S6. Heatmaps of cytokine levels with the common toxicity factors.

Spearman correlation coefficients with adjusted p-values <0.05 are marked with an asterisk. COVID-19 patients (n=63).

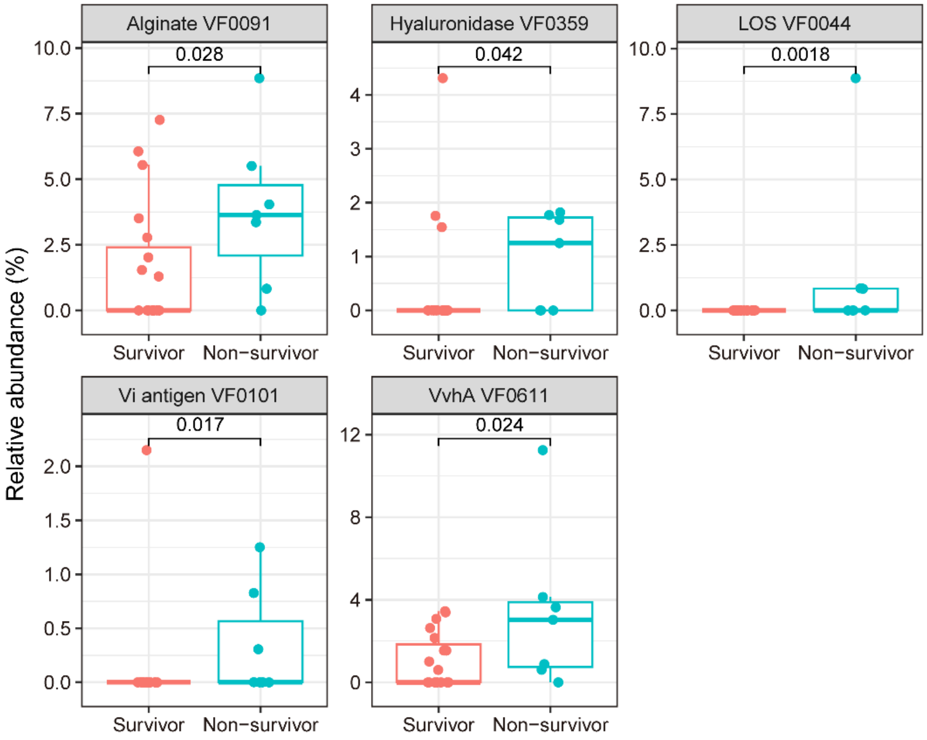

Figure. S7. Significantly different virulence factors between survivors and non-survivors of HAP/CAP patients.

One-tailed wilcoxon rank-sum test was used for significance statistics. HAP/CAP patients (n=27), non-survivors of the HAP/CAP patients (n=7), survivors of the HAP/CAP patients (n=20).

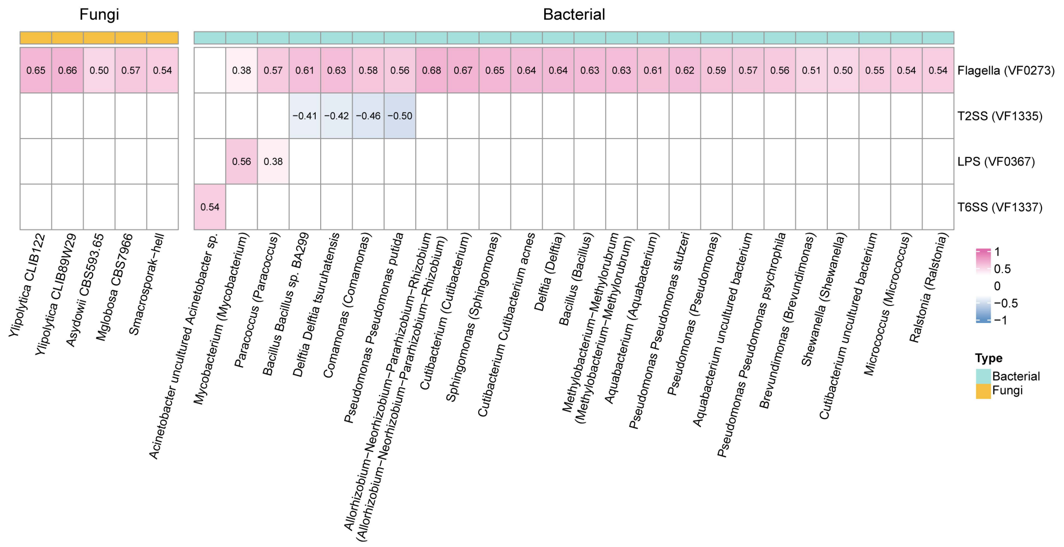

Figure. S8. Correlation analysis between bacterial, fungi gene expression and virulence factors.

Heatmap demonstrating correlation analysis between bacterial, fungi gene expression and virulence factors in invasive ventilated COVID-19 patients. The color-coded positions indicate adjusted p-values < 0.05 and the numbers within each grid represent Spearman correlation coefficients. Only absolute correlation coefficients greater than 0.5 are displayed. COVID-19 patients (n=63).

Table S1. Comparison of two cohorts

	HAP/CAP(n=27) Median (IQR)	COVID-19(n=64) Median (IQR)	*P* value
Age, yr	71.00 (24.00)	80.00 (15.00)	0.08
Sex			0.28
Male，n (%)	23.00(85.19)	48.00(75.00)	
Female，n (%)	4.00(14.81)	16.00(25.00)	

HAP/CAP:Hospital/Community Acquired Pneumonia,IQR=interquartile range, *P* values were evulated by Mann-Whitney test or Chi-square test ,Two-tailed degree of significance between groups was set at $P < 0.05$.

Table S2. The statistics results of genome assembly of the SARS-CoV-2 strains

Samples	Contigs	Largest contig (bp)	Total length (bp)	GC (%)
s10.fa	1	29984	29984	37.88
s11.fa	1	29747	29747	37.85
s12.fa	1	29871	29871	37.93
s14.fa	1	29870	29870	37.86
s16.fa	1	33717	33717	36.71
s18.fa	1	24628	24628	37.37
s1.fa	1	29943	29943	37.89
s20.fa	1	29870	29870	37.82
s22.fa	1	29869	29869	37.81
s23.fa	1	29897	29897	37.92
s24.fa	1	29870	29870	37.86
s25.fa	1	29982	29982	37.83
s28.fa	1	29870	29870	37.82
s29.fa	1	29897	29897	38.03
s2.fa	1	30006	30006	37.84
s31.fa	1	23233	23233	37.58
s34.fa	1	29870	29870	37.88
s35.fa	1	28255	28255	37.49
s3.fa	1	29795	29795	37.86
s4.fa	1	29930	29930	37.89
s5.fa	1	29870	29870	37.82
s6.fa	1	29882	29882	37.82
s7.fa	2	23279	41441	37.93
s8.fa	1	29795	29795	37.87
s9.fa	1	29935	29935	37.87
ss11.fa	1	23403	23403	37.57
ss16.fa	1	29839	29839	37.85
ss18.fa	1	23425	23425	37.55
ss27.fa	1	29861	29861	37.86
ss28.fa	1	29896	29896	37.88
ss4.fa	1	29876	29876	38.16
ss9.fa	1	29862	29862	37.83

Table S3. The statistics results of single nucleotide polymorphism (SNPs) identified in our study

POS	REF	ALT	QUAL	FILTER	INFO	FORMAT	FORMAT_value	TYPE	Reported in 682 genomes
44	C	T	101112.06	PASS	AC=2;AF=1.00;AN=2;DP=371;ExcessHet=0.0000;FS=0.000;MLEAC=2;MLEAF=1.00;MQ=60.00;QD=28.33;SOR=0.855	GT:AD:DP:GQ:PL	1/1:0,357:357:99:10126,1071,0	BA.5	yes
210	G	T	4237.64	PASS	AC=1;AF=0.500;AN=2;BaseQRankSum=-2.200e+00;DP=888;ExcessHet=0.0000;FS=0.588;MLEAC=1;MLEAF=0.500;MQ=60.00;MQRankSum=0.00;QD=4.83;ReadPosRankSum=2.67;SOR=0.614	GT:AD:DP:GQ:PL	0/1:630,247:877:99:4245,0,25460	BA.5	yes
213	G	T	65302.06	PASS	AC=2;AF=1.00;AN=2;DP=1609;ExcessHet=0.0000;FS=0.000;MLEAC=2;MLEAF=1.00;MQ=60.00;QD=25.36;SOR=0.928	GT:AD:DP:GQ:PL	1/1:0,1586:1586:99:65316,4770,0	BA.5	yes
241	C	T	205505.1	PASS	AC=2;AF=1.00;AN=2;DP=5558;ExcessHet=0.0000;FS=0.000;MLEAC=2;MLEAF=1.00;MQ=60.00;QD=25.36;SOR=0.818	GT:AD:DP:GQ:PL	1/1:0,5405:5407:99:205519,16246,0	BA.5	yes
407	G	T	37.32	PASS	AC=2;AF=1.00;AN=2;DP=2;ExcessHet=0.0000;FS=0.000;MLEAC=1;MLEAF=0.500;MQ=60.00;QD=18.66;SOR=0.693	GT:AD:DP:GQ:PL	1/1:0,2:2:6:49,6,0	BA.5	no
410	G	A	37.32	PASS	AC=2;AF=1.00;AN=2;DP=2;ExcessHet=0.0000;FS=0.000;MLEAC=1;MLEAF=0.500;MQ=60.00;QD=18.66;SOR=0.693	GT:AD:DP:GQ:PL	1/1:0,2:2:6:49,6,0	BA.5	no

628	T	G	43.64	PASS	AC=1;AF=0.500;AN=2;BaseQRankSum=-4.970e-01;DP=15;ExcessHet=0.0000;FS=0.000;MLEAC=1;MLEAF=0.500;MQ=60.00;MQRankSum=0.00;QD=3.36;ReadPosRankSum=-2.093e+00;SOR=0.551	GT:AD:DP:GQ:PGT:PID:PL:PS	0|1:11,2:13:51:0|1:628_T_G:51,0,456:628	BA.5	no
631	T	A	43.64	PASS	AC=1;AF=0.500;AN=2;BaseQRankSum=-6.740e-01;DP=13;ExcessHet=0.0000;FS=0.000;MLEAC=1;MLEAF=0.500;MQ=60.00;MQRankSum=0.00;QD=3.36;ReadPosRankSum=-2.093e+00;SOR=0.551	GT:AD:DP:GQ:PGT:PID:PL:PS	0|1:11,2:13:51:0|1:628_T_G:51,0,456:628	BA.5	no
633	T	G	43.64	PASS	AC=1;AF=0.500;AN=2;BaseQRankSum=-6.740e-01;DP=13;ExcessHet=0.0000;FS=0.000;MLEAC=1;MLEAF=0.500;MQ=60.00;MQRankSum=0.00;QD=3.36;ReadPosRankSum=-2.102e+00;SOR=0.551	GT:AD:DP:GQ:PGT:PID:PL:PS	0|1:11,2:13:51:0|1:628_T_G:51,0,456:628	BA.5	no
638	A	T	35.48	PASS	AC=2;AF=1.00;AN=2;DP=1;ExcessHet=0.0000;FS=0.000;MLEAC=1;MLEAF=0.500;MQ=60.00;QD=25.36;SOR=1.609	GT:AD:DP:GQ:PGT:PID:PL:PS	1|1:0,1:1:3:1|1:630_T_TGCC:45,3,0:630	BA.5	no
640	G	A	35.48	PASS	AC=2;AF=1.00;AN=2;DP=1;ExcessHet=0.0000;FS=0.000;MLEAC=1;MLEAF=0.500;MQ=60.00;QD=28.73;SOR=1.609	GT:AD:DP:GQ:PG	1|1:0,1:1:3:1|1:630_T_TGCC:45	BA.5	no

						T:PID:PL:PS	,3,0:630		
643	C	G	35.48	PASS	AC=2;AF=1.00;AN=2;DP=1;ExcessHet=0.0000;FS=0.000;MLEAC=1;MLEAF=0.500;MQ=60.00;QD=30.97;SOR=1.609	GT:AD:DP:GQ:PGT:PID:PL:PS	1|1:0,1:1:3:1|1:630_T_TGCC:45,3,0:630	BA.5	no
670	T	G	228612.1	PASS	AC=2;AF=1.00;AN=2;BaseQRankSum=4.07;DP=5984;ExcessHet=0.0000;FS=0.000;MLEAC=2;MLEAF=1.00;MQ=60.00;MQRankSum=-4.400e-02;QD=28.73;ReadPosRankSum=1.39;SOR=0.353	GT:AD:DP:GQ:PL	1/1:1,5811:5829:99:228626,17484,0	BA.5	yes
706	C	T	31261.06	PASS	AC=2;AF=1.00;AN=2;DP=1140;ExcessHet=0.0000;FS=0.000;MLEAC=2;MLEAF=1.00;MQ=60.00;QD=28.14;SOR=0.739	GT:AD:DP:GQ:PL	1/1:0,1111:1111:99:31275,3332,0	BA.5	no
767	G	A	35.48	PASS	AC=2;AF=1.00;AN=2;DP=1;ExcessHet=0.0000;FS=0.000;MLEAC=1;MLEAF=0.500;MQ=60.00;QD=25.36;SOR=1.609	GT:AD:DP:GQ:PGT:PID:PL:PS	1|1:0,1:1:3:1|1:767_G_A:45,3,0:767	BA.5	no
771	T	A	35.48	PASS	AC=2;AF=1.00;AN=2;DP=1;ExcessHet=0.0000;FS=0.000;MLEAC=1;MLEAF=0.500;MQ=60.00;QD=30.97;SOR=1.609	GT:AD:DP:GQ:PGT:PID:	1|1:0,1:1:3:1|1:767_G_A:45,3,0:767	BA.5	no

						PL:PS			
851	T	C	32.64	PASS	AC=1;AF=0.500;AN=2;BaseQRankSum=-9.670e-01;DP=3;ExcessHet=0.0000;FS=0.000;MLEAC=1;MLEAF=0.500;MQ=60.00;MQRankSum=0.00;QD=10.88;ReadPosRankSum=0.967;SOR=0.223	GT:AD:DP:GQ:PL	0/1:1,2:3:33:40,0,33	BA.5	no
1012	T	A	35.48	PASS	AC=2;AF=1.00;AN=2;DP=1;ExcessHet=0.0000;FS=0.000;MLEAC=1;MLEAF=0.500;MQ=60.00;QD=30.62;SOR=1.609	GT:AD:DP:GQ:PGT:PID:PL:PS	1|1:0,1:1:3:1|1:1007_AG_A:45,3,0:1007	BA.5	no
1013	G	C	35.48	PASS	AC=2;AF=1.00;AN=2;DP=1;ExcessHet=0.0000;FS=0.000;MLEAC=1;MLEAF=0.500;MQ=60.00;QD=28.17;SOR=1.609	GT:AD:DP:GQ:PGT:PID:PL:PS	1|1:0,1:1:3:1|1:1007_AG_A:45,3,0:1007	BA.5	no
1015	A	T	35.48	PASS	AC=2;AF=1.00;AN=2;DP=1;ExcessHet=0.0000;FS=0.000;MLEAC=1;MLEAF=0.500;MQ=60.00;QD=26.80;SOR=1.609	GT:AD:DP:GQ:PGT:PID:PL:PS	1|1:0,1:1:3:1|1:1007_AG_A:45,3,0:1007	BA.5	no
1085	G	T	3685.64	PASS	AC=1;AF=0.500;AN=2;BaseQRankSum=-1.051e+00;DP=897;ExcessHet=0.0000;FS=0.610;MLEAC=1;MLEAF=0.500;MQ=60.00;MQRankSum=0.00;Q	GT:AD:DP:GQ:PL	0/1:665,224:889:99:3693,0,16580	BA.5	yes

					D=4.15;ReadPosRankSum=0.274;SOR=0.762				
1207	A	T	35.48	PASS	AC=2;AF=1.00;AN=2;DP=1;ExcessHet=0.0000;FS=0.000;MLEAC=1;MLEAF=0.500;MQ=60.00;QD=26.00;SOR=1.609	GT:AD:DP:GQ:PGT:PID:PL:PS	1|1:0,1:1:3:1|1:1207_A_T:45,3,0:1207	BA.5	no
1210	G	A	35.48	PASS	AC=2;AF=1.00;AN=2;DP=1;ExcessHet=0.0000;FS=0.000;MLEAC=1;MLEAF=0.500;MQ=60.00;QD=30.02;SOR=1.609	GT:AD:DP:GQ:PGT:PID:PL:PS	1|1:0,1:1:3:1|1:1207_A_T:45,3,0:1207	BA.5	no
1468	T	G	67.64	PASS	AC=1;AF=0.500;AN=2;BaseQRankSum=0.00;DP=5;ExcessHet=0.0000;FS=0.000;MLEAC=1;MLEAF=0.500;MQ=60.00;MQRankSum=0.00;QD=13.53;ReadPosRankSum=-6.740e-01;SOR=0.368	GT:AD:DP:GQ:PGT:PID:PL:PS	0|1:3,2:5:75:0|1:1468_T_G:75,0,100:1468	BA.5	no
1495	G	A	35.48	PASS	AC=2;AF=1.00;AN=2;DP=1;ExcessHet=0.0000;FS=0.000;MLEAC=1;MLEAF=0.500;MQ=60.00;QD=31.98;SOR=1.609	GT:AD:DP:GQ:PGT:PID:PL:PS	1|1:0,1:1:3:1|1:1495_G_A:45,3,0:1495	BA.5	no
1498	C	T	35.48	PASS	AC=2;AF=1.00;AN=2;DP=1;ExcessHet=0.0000;FS=0.000;MLEAC=1;MLEAF=0.5	GT:AD:DP:	1|1:0,1:1:3:1|1:1495_G_A:	BA.5	no

					00;MQ=60.00;QD=27.51;SOR=1.609	GQ:PGT:PID:PL:PS	45,3,0:1495		
1499	T	C	35.48	PASS	AC=2;AF=1.00;AN=2;DP=1;ExcessHet=0.0000;FS=0.000;MLEAC=1;MLEAF=0.500;MQ=60.00;QD=29.11;SOR=1.609	GT:AD:DP:GQ:PGT:PID:PL:PS	1|1:0,1:1:3:1|1:1495_G_A:45,3,0:1495	BA.5	no
1509	G	A	70.64	PASS	AC=1;AF=0.500;AN=2;BaseQRankSum=0.00;DP=4;ExcessHet=0.0000;FS=0.000;MLEAC=1;MLEAF=0.500;MQ=60.00;MQRankSum=0.00;QD=17.66;ReadPosRankSum=1.38;SOR=0.693	GT:AD:DP:GQ:PGT:PID:PL:PS	0|1:2,2:4:78:0|1:1509_G_A:78,0,78:1509	BA.5	no
1512	G	C	70.64	PASS	AC=1;AF=0.500;AN=2;BaseQRankSum=0.00;DP=4;ExcessHet=0.0000;FS=0.000;MLEAC=1;MLEAF=0.500;MQ=60.00;MQRankSum=0.00;QD=17.66;ReadPosRankSum=-1.383e+00;SOR=0.693	GT:AD:DP:GQ:PGT:PID:PL:PS	0|1:2,2:4:78:0|1:1509_G_A:78,0,78:1509	BA.5	no
1515	A	T	70.64	PASS	AC=1;AF=0.500;AN=2;BaseQRankSum=0.00;DP=4;ExcessHet=0.0000;FS=0.000;MLEAC=1;MLEAF=0.500;MQ=60.00;MQRankSum=0.00;QD=17.66;ReadPosRankSum=-1.383e+00;SOR=0.693	GT:AD:DP:GQ:PGT:PID:PL:PS	0|1:2,2:4:78:0|1:1509_G_A:78,0,78:1509	BA.5	no

1627	C	T	205094.1	PASS	AC=2;AF=1.00;AN=2;BaseQRankSum=1.38;DP=5876;ExcessHet=0.0000;FS=0.000;MLEAC=2;MLEAF=1.00;MQ=60.00;MQRankSum=0.00;QD=30.97;ReadPosRankSum=2.85;SOR=0.381	GT:AD:DP:GQ:PL	1/1:5,3760:3781:99:205108,15900,0	BA.5	yes
1799	A	G	35.48	PASS	AC=2;AF=1.00;AN=2;DP=1;ExcessHet=0.0000;FS=0.000;MLEAC=1;MLEAF=0.500;MQ=60.00;QD=28.08;SOR=1.609	GT:AD:DP:GQ:PGT:PID:PL:PS	1|1:0,1:1:3:1|1:1799_A_G:45,3,0:1799	BA.5	no
1801	A	T	35.48	PASS	AC=2;AF=1.00;AN=2;DP=1;ExcessHet=0.0000;FS=0.000;MLEAC=1;MLEAF=0.500;MQ=60.00;QD=23.23;SOR=1.609	GT:AD:DP:GQ:PGT:PID:PL:PS	1|1:0,1:1:3:1|1:1799_A_G:45,3,0:1799	BA.5	no
1917	C	T	301.06	PASS	AC=2;AF=1.00;AN=2;DP=13;ExcessHet=0.0000;FS=0.000;MLEAC=2;MLEAF=1.00;MQ=60.00;QD=27.37;SOR=1.270	GT:AD:DP:GQ:PL	1/1:0,11:11:33:315,33,0	BA.5	no
2245	C	A	78.32	PASS	AC=2;AF=1.00;AN=2;DP=2;ExcessHet=0.0000;FS=0.000;MLEAC=1;MLEAF=0.500;MQ=60.00;QD=27.24;SOR=0.693	GT:AD:DP:GQ:PGT:PID:PL:PS	1|1:0,2:2:6:1|1:2245_C_A:90,6,0:2245	BA.5	no
2247	G	T	78.32	PA	AC=2;AF=1.00;AN=2;DP=2;ExcessHet=0.0000;FS=0.000;MLEAC=1;MLEAF=0.5	GT:AD:	1|1:0,2:2:6:1|1:2245	BA.5	no

				SS	00;MQ=60.00;QD=28.20;SOR=0.693	DP:GQ:PGT:PID:PL:PS	_C_A:90,6,0:2245		
2250	G	A	78.32	PASS	AC=2;AF=1.00;AN=2;DP=2;ExcessHet=0.0000;FS=0.000;MLEAC=1;MLEAF=0.500;MQ=60.00;QD=25.00;SOR=0.693	GT:AD:DP:GQ:PGT:PID:PL:PS	1|1:0,2:2:6:1|1:2245_C_A:90,6,0:2245	BA.5	no
2252	C	G	78.32	PASS	AC=2;AF=1.00;AN=2;DP=2;ExcessHet=0.0000;FS=0.000;MLEAC=1;MLEAF=0.500;MQ=60.00;QD=29.56;SOR=0.693	GT:AD:DP:GQ:PGT:PID:PL:PS	1|1:0,2:2:6:1|1:2245_C_A:90,6,0:2245	BA.5	no
2444	A	T	35.48	PASS	AC=2;AF=1.00;AN=2;DP=1;ExcessHet=0.0000;FS=0.000;MLEAC=1;MLEAF=0.500;MQ=60.00;QD=29.03;SOR=1.609	GT:AD:DP:GQ:PGT:PID:PL:PS	1|1:0,1:1:3:1|1:2444_A_T:45,3,0:2444	BA.5	no
2450	C	T	35.48	PASS	AC=2;AF=1.00;AN=2;DP=1;ExcessHet=0.0000;FS=0.000;MLEAC=1;MLEAF=0.500;MQ=60.00;QD=28.73;SOR=1.609	GT:AD:DP:GQ:PGT:PID:	1|1:0,1:1:3:1|1:2450_C_T:45,3,0:2450	BA.5	no

						PL:PS			
2542	A	T	35.48	PASS	AC=2;AF=1.00;AN=2;DP=1;ExcessHet=0.0000;FS=0.000;MLEAC=1;MLEAF=0.500;MQ=60.00;QD=34.42;SOR=1.609	GT:AD:DP:GQ:PGT:PID:PL:PS	1|1:0,1:1:3:1|1:2532_TTGTC_T:45,3,0:2532	BA.5	yes
2710	C	T	201017.1	PASS	AC=2;AF=1.00;AN=2;BaseQRankSum=-1.562e+00;DP=5481;ExcessHet=0.0000;FS=0.000;MLEAC=2;MLEAF=1.00;MQ=60.00;MQRankSum=-2.840e-01;QD=27.24;ReadPosRankSum=-1.641e+00;SOR=0.434	GT:AD:DP:GQ:PL	1/1:6,5249:5300:99:201031,14874,0	BA.5	yes
2790	C	T	193122.1	PASS	AC=2;AF=1.00;AN=2;BaseQRankSum=-5.310e-01;DP=5275;ExcessHet=0.0000;FS=0.000;MLEAC=2;MLEAF=1.00;MQ=60.00;MQRankSum=-5.690e-01;QD=28.20;ReadPosRankSum=1.75;SOR=0.262	GT:AD:DP:GQ:PL	1/1:1,4995:5000:99:193136,15147,0	BA.5	yes
2822	C	A	31.64	PASS	AC=1;AF=0.500;AN=2;BaseQRankSum=-6.740e-01;DP=2;ExcessHet=0.0000;FS=0.000;MLEAC=1;MLEAF=0.500;MQ=60.00;MQRankSum=0.00;QD=15.82;ReadPosRankSum=-6.740e-01;SOR=0.693	GT:AD:DP:GQ:PGT:PID:PL:PS	0|1:1,1:2:39:0|1:2821_A_AT:39,0,39:2821	BA.5	no
3037	C	T	214844.1	PASS	AC=2;AF=1.00;AN=2;BaseQRankSum=-6.890e-01;DP=5743;ExcessHet=0.0000;FS=0.000;MLEAC=2;MLEAF=1.00;MQ=60.00;MQRankSum=-5.660e-	GT:AD:DP:GQ:PL	1/1:2,5586:5588:99:214858,16754,0	BA.5	yes

<table>
<tr><td></td><td></td><td></td><td></td><td></td><td>01;QD=25.00;ReadPosRankSum=2.26;SOR=0.142</td><td></td><td></td><td></td><td></td></tr>
<tr><td>3537</td><td>T</td><td>C</td><td>64.64</td><td>PASS</td><td>AC=1;AF=0.500;AN=2;BaseQRankSum=0.00;DP=6;ExcessHet=0.0000;FS=0.000;MLEAC=1;MLEAF=0.500;MQ=60.00;MQRankSum=0.00;QD=10.77;ReadPosRankSum=-1.834e+00;SOR=0.693</td><td>GT:AD:DP:GQ:PGT:PID:PL:PS</td><td>0|1:4,2:6:72:0|1:3537_T_C:72,0,162:3537</td><td>BA.5</td><td>no</td></tr>
<tr><td>3539</td><td>G</td><td>A</td><td>64.64</td><td>PASS</td><td>AC=1;AF=0.500;AN=2;BaseQRankSum=0.00;DP=6;ExcessHet=0.0000;FS=0.000;MLEAC=1;MLEAF=0.500;MQ=60.00;MQRankSum=0.00;QD=10.77;ReadPosRankSum=-1.834e+00;SOR=0.693</td><td>GT:AD:DP:GQ:PGT:PID:PL:PS</td><td>0|1:4,2:6:72:0|1:3537_T_C:72,0,162:3537</td><td>BA.5</td><td>no</td></tr>
<tr><td>3773</td><td>C</td><td>A</td><td>37.32</td><td>PASS</td><td>AC=2;AF=1.00;AN=2;DP=2;ExcessHet=0.0000;FS=0.000;MLEAC=1;MLEAF=0.500;MQ=60.00;QD=18.66;SOR=0.693</td><td>GT:AD:DP:GQ:PL</td><td>1/1:0,2:2:6:49,6,0</td><td>BA.5</td><td>no</td></tr>
<tr><td>3949</td><td>T</td><td>A</td><td>35.48</td><td>PASS</td><td>AC=2;AF=1.00;AN=2;DP=1;ExcessHet=0.0000;FS=0.000;MLEAC=1;MLEAF=0.500;MQ=60.00;QD=28.53;SOR=1.609</td><td>GT:AD:DP:GQ:PGT:PID:PL:PS</td><td>1|1:0,1:1:3:1|1:3949_T_A:45,3,0:3949</td><td>BA.5</td><td>no</td></tr>
<tr><td>3950</td><td>G</td><td>A</td><td>35.48</td><td>PASS</td><td>AC=2;AF=1.00;AN=2;DP=1;ExcessHet=0.0000;FS=0.000;MLEAC=1;MLEAF=0.500;MQ=60.00;QD=27.23;SOR=1.609</td><td>GT:AD:DP:GQ:PGT:PID:</td><td>1|1:0,1:1:3:1|1:3949_T_A:45,3,0:3949</td><td>BA.5</td><td>no</td></tr>
</table>

						PL:PS			
3967	T	A	35.48	PASS	AC=2;AF=1.00;AN=2;DP=1;ExcessHet=0.0000;FS=0.000;MLEAC=1;MLEAF=0.500;MQ=60.00;QD=28.17;SOR=1.609	GT:AD:DP:GQ:PGT:PID:PL:PS	1|1:0,1:1:3:1|1:3967_T_A:45,3,0:3967	BA.5	no
3969	G	T	35.48	PASS	AC=2;AF=1.00;AN=2;DP=1;ExcessHet=0.0000;FS=0.000;MLEAC=1;MLEAF=0.500;MQ=60.00;QD=26.80;SOR=1.609	GT:AD:DP:GQ:PGT:PID:PL:PS	1|1:0,1:1:3:1|1:3967_T_A:45,3,0:3967	BA.5	no
3970	T	A	35.48	PASS	AC=2;AF=1.00;AN=2;DP=1;ExcessHet=0.0000;FS=0.000;MLEAC=1;MLEAF=0.500;MQ=60.00;QD=26.00;SOR=1.609	GT:AD:DP:GQ:PGT:PID:PL:PS	1|1:0,1:1:3:1|1:3967_T_A:45,3,0:3967	BA.5	no
3971	G	A	35.48	PASS	AC=2;AF=1.00;AN=2;DP=1;ExcessHet=0.0000;FS=0.000;MLEAC=1;MLEAF=0.500;MQ=60.00;QD=30.02;SOR=1.609	GT:AD:DP:GQ:PGT:PID:PL:PS	1|1:0,1:1:3:1|1:3967_T_A:45,3,0:3967	BA.5	no
3989	A	G	78.32	PASS	AC=2;AF=1.00;AN=2;DP=2;ExcessHet=0.0000;FS=0.000;MLEAC=1;MLEAF=0.5	GT:AD:DP:	1|1:0,2:2:6:1|1:3989_A_G:	BA.5	no

<table>
<tr><th></th><th></th><th></th><th></th><th></th><th></th><th></th><th></th><th></th><th></th></tr>
<tr><td></td><td></td><td></td><td></td><td></td><td>00;MQ=60.00;QD=31.98;SOR=0.693</td><td>GQ:PGT:PID:PL:PS</td><td>90,6,0:3989</td><td></td><td></td></tr>
<tr><td>3992</td><td>C</td><td>T</td><td>78.32</td><td>PASS</td><td>AC=2;AF=1.00;AN=2;DP=2;ExcessHet=0.0000;FS=0.000;MLEAC=1;MLEAF=0.500;MQ=60.00;QD=29.11;SOR=0.693</td><td>GT:AD:DP:GQ:PGT:PID:PL:PS</td><td>1|1:0,2:2:6:1|1:3989_A_G:90,6,0:3989</td><td>BA.5</td><td>no</td></tr>
<tr><td>3996</td><td>A</td><td>C</td><td>78.32</td><td>PASS</td><td>AC=2;AF=1.00;AN=2;DP=2;ExcessHet=0.0000;FS=0.000;MLEAC=1;MLEAF=0.500;MQ=60.00;QD=28.08;SOR=0.693</td><td>GT:AD:DP:GQ:PGT:PID:PL:PS</td><td>1|1:0,2:2:6:1|1:3989_A_G:90,6,0:3989</td><td>BA.5</td><td>no</td></tr>
<tr><td>4184</td><td>G</td><td>A</td><td>196361.1</td><td>PASS</td><td>AC=2;AF=1.00;AN=2;BaseQRankSum=-9.850e-01;DP=5373;ExcessHet=0.0000;FS=0.000;MLEAC=2;MLEAF=1.00;MQ=60.00;MQRankSum=0.00;QD=29.56;ReadPosRankSum=0.770;SOR=0.870</td><td>GT:AD:DP:GQ:PL</td><td>1/1:2,4481:4485:99:196375,15460,0</td><td>BA.5</td><td>yes</td></tr>
<tr><td>4299</td><td>T</td><td>G</td><td>67.64</td><td>PASS</td><td>AC=1;AF=0.500;AN=2;BaseQRankSum=-5.240e-01;DP=5;ExcessHet=0.0000;FS=0.000;MLEAC=1;MLEAF=0.500;MQ=60.00;MQRankSum=0.00;QD=13.53;ReadPosRankSum=-1.645e+00;SOR=0.368</td><td>GT:AD:DP:GQ:PGT:PID:PL:PS</td><td>0|1:3,2:5:75:0|1:4299_T_G:75,0,120:4299</td><td>BA.5</td><td>no</td></tr>
<tr><td>4321</td><td>C</td><td>T</td><td>1851</td><td>PA</td><td>AC=2;AF=1.00;AN=2;BaseQRankSum=4.10;DP=5184;</td><td>GT:A</td><td>1/1:16,4975:</td><td>BA.5</td><td>yes</td></tr>
</table>

			32.1	SS	ExcessHet=0.0000;FS=0.000;MLEAC=2;MLEAF=1.00;MQ=60.00;MQRankSum=0.141;QD=30.62;ReadPosRankSum=5.64;SOR=0.130	D:DP:GQ:PL	4998:99:185146,14114,0		
4391	C	A	178083.1	PASS	AC=2;AF=1.00;AN=2;DP=4576;ExcessHet=0.0000;FS=0.000;MLEAC=2;MLEAF=1.00;MQ=60.00;QD=29.56;SOR=0.986	GT:AD:DP:GQ:PGT:PID:PL:PS	1|1:0,4490:4490:99:1|1:4321_C_T:178097,13500,0:4321	BA.5	no
4454	G	C	31.64	PASS	AC=1;AF=0.500;AN=2;BaseQRankSum=-6.740e-01;DP=2;ExcessHet=0.0000;FS=0.000;MLEAC=1;MLEAF=0.500;MQ=60.00;MQRankSum=0.00;QD=15.82;ReadPosRankSum=-6.740e-01;SOR=0.693	GT:AD:DP:GQ:PGT:PID:PL:PS	0|1:1,1:2:39:0|1:4446_A_AACTGTGAGTC:39,0,39:4446	BA.5	no
4502	G	C	78.32	PASS	AC=2;AF=1.00;AN=2;DP=2;ExcessHet=0.0000;FS=0.000;MLEAC=1;MLEAF=0.500;MQ=60.00;QD=29.52;SOR=0.693	GT:AD:DP:GQ:PGT:PID:PL:PS	1|1:0,2:2:6:1|1:4502_G_C:90,6,0:4502	BA.5	no
4505	G	A	78.32	PASS	AC=2;AF=1.00;AN=2;DP=2;ExcessHet=0.0000;FS=0.000;MLEAC=1;MLEAF=0.500;MQ=60.00;QD=33.47;SOR=0.693	GT:AD:DP:GQ:PGT:PID:PL:PS	1|1:0,2:2:6:1|1:4502_G_C:90,6,0:4502	BA.5	no

4511	G	A	78.32	PASS	AC=2;AF=1.00;AN=2;DP=2;ExcessHet=0.0000;FS=0.000;MLEAC=1;MLEAF=0.500;MQ=60.00;QD=32.91;SOR=0.693	GT:AD:DP:GQ:PGT:PID:PL:PS	1|1:0,2:2:6:1|1:4502_G_C:90,6,0:4502	BA.5	no
4695	T	A	64.64	PASS	AC=1;AF=0.500;AN=2;BaseQRankSum=0.00;DP=6;ExcessHet=0.0000;FS=0.000;MLEAC=1;MLEAF=0.500;MQ=60.00;MQRankSum=0.00;QD=10.77;ReadPosRankSum=-1.834e+00;SOR=0.693	GT:AD:DP:GQ:PL	0/1:4,2:6:72:72,0,162	BA.5	no
4703	C	G	70.64	PASS	AC=1;AF=0.500;AN=2;DP=4;ExcessHet=0.0000;FS=0.000;MLEAC=1;MLEAF=0.500;MQ=60.00;MQRankSum=0.00;QD=17.66;SOR=0.693	GT:AD:DP:GQ:PGT:PID:PL:PS	0|1:2,2:4:38:0|1:4699_T_TTGAGGGAAAACAGGG:78,0,38:4699	BA.5	no
4704	C	T	78.32	PASS	AC=2;AF=1.00;AN=2;DP=2;ExcessHet=0.0000;FS=0.000;MLEAC=1;MLEAF=0.500;MQ=60.00;QD=31.45;SOR=0.693	GT:AD:DP:GQ:PGT:PID:PL:PS	1|1:0,2:2:6:1|1:4699_T_TTGAGGGAAAACAGGG:90,6,0:4699	BA.5	no
4706	G	C	78.32	PASS	AC=2;AF=1.00;AN=2;DP=2;ExcessHet=0.0000;FS=0.000;MLEAC=1;MLEAF=0.500;MQ=60.00;QD=33.27;SOR=0.693	GT:AD:DP:GQ:PG	1|1:0,2:2:6:1|1:4699_T_TTGAGGGA	BA.5	no

						T:PID:PL:PS	AAACAGGG:90,6,0:4699		
4815	C	T	31.64	PASS	AC=1;AF=0.500;AN=2;BaseQRankSum=2.36;DP=15;ExcessHet=0.0000;FS=3.310;MLEAC=1;MLEAF=0.500;MQ=60.00;MQRankSum=0.00;QD=2.11;ReadPosRankSum=1.62;SOR=2.279	GT:AD:DP:GQ:PL	0/1:13,2:15:39:39,0,315	BA.5	no
4926	A	G	70.64	PASS	AC=1;AF=0.500;AN=2;BaseQRankSum=0.00;DP=4;ExcessHet=0.0000;FS=0.000;MLEAC=1;MLEAF=0.500;MQ=60.00;MQRankSum=0.00;QD=17.66;ReadPosRankSum=-1.383e+00;SOR=0.693	GT:AD:DP:GQ:PGT:PID:PL:PS	0|1:2,2:4:78:0|1:4922_T_TGATTTCAGAGAGC:78,0,78:4922	BA.5	no
4928	A	G	70.64	PASS	AC=1;AF=0.500;AN=2;BaseQRankSum=0.00;DP=6;ExcessHet=0.0000;FS=0.000;MLEAC=1;MLEAF=0.500;MQ=60.00;MQRankSum=0.00;QD=17.66;ReadPosRankSum=-1.383e+00;SOR=0.693	GT:AD:DP:GQ:PGT:PID:PL:PS	0|1:2,2:4:78:0|1:4922_T_TGATTTCAGAGAGC:78,0,78:4922	BA.5	no
5310	C	T	65.64	PASS	AC=1;AF=0.500;AN=2;BaseQRankSum=0.00;DP=6;ExcessHet=0.0000;FS=0.000;MLEAC=1;MLEAF=0.500;MQ=60.00;MQRankSum=0.00;QD=10.94;ReadPosRankSum=0.598;SOR=0.693	GT:AD:DP:GQ:PL	0/1:3,3:6:73:73,0,73	BA.5	no
5314	C	T	55.64	PASS	AC=1;AF=0.500;AN=2;BaseQRankSum=-2.820e-01;DP=9;ExcessHet=0.0000;FS=0.000;MLEAC=1;MLEAF=0.500;MQ=60.00;MQRankSum=0.00;QD=6.18;Rea	GT:AD:DP:GQ:PGT:P	0|1:7,2:9:63:0|1:5314_C_T:63,0,288:5314	BA.5	no

					dPosRankSum=-8.120e-01;SOR=0.495	ID:PL:PS			
5519	T	G	37.32	PASS	AC=2;AF=1.00;AN=2;DP=2;ExcessHet=0.0000;FS=0.000;MLEAC=1;MLEAF=0.500;MQ=60.00;QD=18.66;SOR=0.693	GT:AD:DP:GQ:PL	1/1:0,2:2:6:49,6,0	BA.5	no
5574	A	G	78.32	PASS	AC=2;AF=1.00;AN=2;DP=2;ExcessHet=0.0000;FS=0.000;MLEAC=1;MLEAF=0.500;MQ=60.00;QD=27.08;SOR=0.693	GT:AD:DP:GQ:PGT:PID:PL:PS	1|1:0,2:2:6:1|1:5566_T_TG:90,6,0:5566	BA.5	no
5575	C	T	78.32	PASS	AC=2;AF=1.00;AN=2;DP=2;ExcessHet=0.0000;FS=0.000;MLEAC=1;MLEAF=0.500;MQ=60.00;QD=25.42;SOR=0.693	GT:AD:DP:GQ:PGT:PID:PL:PS	1|1:0,2:2:6:1|1:5566_T_TG:90,6,0:5566	BA.5	no
5576	A	G	78.32	PASS	AC=2;AF=1.00;AN=2;DP=2;ExcessHet=0.0000;FS=0.000;MLEAC=1;MLEAF=0.500;MQ=60.00;QD=27.65;SOR=0.693	GT:AD:DP:GQ:PGT:PID:PL:PS	1|1:0,2:2:6:1|1:5566_T_TG:90,6,0:5566	BA.5	no
5765	G	A	251.64	PASS	AC=1;AF=0.500;AN=2;BaseQRankSum=1.79;DP=47;ExcessHet=0.0000;FS=0.000;MLEAC=1;MLEAF=0.500;MQ=60.00;MQRankSum=0.00;QD=5.59;ReadPosRankSum=-4.345e+00;SOR=0.892	GT:AD:DP:GQ:PGT:P	0|1:36,9:45:99:0|1:5765_G_A:259,0,14	BA.5	no

						ID:PL:PS	59:5765		
5766	G	C	251.64	PASS	AC=1;AF=0.500;AN=2;BaseQRankSum=0.295;DP=45;ExcessHet=0.0000;FS=0.000;MLEAC=1;MLEAF=0.500;MQ=60.00;MQRankSum=0.00;QD=5.59;ReadPosRankSum=-4.342e+00;SOR=0.892	GT:AD:DP:GQ:PGT:PID:PL:PS	0|1:36,9:45:99:0|1:5765_G_A:259,0,1459:5765	BA.5	no
5842	C	T	184.04	PASS	AC=2;AF=1.00;AN=2;DP=10;ExcessHet=0.0000;FS=0.000;MLEAC=2;MLEAF=1.00;MQ=60.00;QD=23.00;SOR=0.693	GT:AD:DP:GQ:PL	1/1:0,8:8:24:198,24,0	BA.5	no
6628	C	T	60.84	PASS	AC=2;AF=1.00;AN=2;DP=3;ExcessHet=0.0000;FS=0.000;MLEAC=1;MLEAF=0.500;MQ=60.00;QD=20.28;SOR=1.179	GT:AD:DP:GQ:PL	1/1:0,3:3:9:74,9,0	BA.5	yes
6762	C	A	40625.06	PASS	AC=2;AF=1.00;AN=2;BaseQRankSum=0.631;DP=1434;ExcessHet=0.0000;FS=0.000;MLEAC=2;MLEAF=1.00;MQ=60.00;MQRankSum=0.00;QD=30.14;ReadPosRankSum=0.501;SOR=0.278	GT:AD:DP:GQ:PL	1/1:1,1347:1348:99:40639,4004,0	BA.5	yes
6794	C	A	78.32	PASS	AC=2;AF=1.00;AN=2;DP=2;ExcessHet=0.0000;FS=0.000;MLEAC=1;MLEAF=0.500;MQ=60.00;QD=30.67;SOR=0.693	GT:AD:DP:GQ:PGT:PID:PL:PS	1|1:0,2:2:6:1|1:6792_T_TA:90,6,0:6792	BA.5	no
6931	T	G	108.64	PASS	AC=1;AF=0.500;AN=2;BaseQRankSum=-3.190e-01;DP=8;ExcessHet=0.0000;FS=0.000;MLEAC=1;MLE	GT:AD:DP:	0/1:3,5:8:67:116,0,67	BA.5	no

					AF=0.500;MQ=60.00;MQRankSum=0.00;QD=13.58;ReadPosRankSum=-1.292e+00;SOR=0.799	GQ:PL			
7029	C	T	132491.1	PASS	AC=2;AF=1.00;AN=2;BaseQRankSum=-2.040e+00;DP=4081;ExcessHet=0.0000;FS=0.000;MLEAC=2;MLEAF=1.00;MQ=60.00;MQRankSum=0.00;QD=34.79;ReadPosRankSum=0.685;SOR=0.168	GT:AD:DP:GQ:PL	1/1:10,3798:3813:99:132505,11013,0	BA.5	yes
7471	C	T	85.14	PASS	AC=2;AF=1.00;AN=2;DP=5;ExcessHet=0.0000;FS=0.000;MLEAC=2;MLEAF=1.00;MQ=60.00;QD=21.29;SOR=0.693	GT:AD:DP:GQ:PL	1/1:0,4:4:12:99,12,0	BA.5	no
7528	C	T	3081.64	PASS	AC=1;AF=0.500;AN=2;BaseQRankSum=-7.310e-01;DP=700;ExcessHet=0.0000;FS=1.247;MLEAC=1;MLEAF=0.500;MQ=60.00;MQRankSum=0.00;QD=4.49;ReadPosRankSum=0.977;SOR=0.842	GT:AD:DP:GQ:PL	0/1:500,187:687:99:3089,0,12119	BA.5	yes
7851	C	T	7035.06	PASS	AC=2;AF=1.00;AN=2;BaseQRankSum=0.933;DP=262;ExcessHet=0.0000;FS=0.000;MLEAC=2;MLEAF=1.00;MQ=60.00;MQRankSum=0.00;QD=29.31;ReadPosRankSum=-2.610e-01;SOR=1.019	GT:AD:DP:GQ:PL	1/1:2,238:240:99:7049,671,0	BA.5	no
8255	G	A	55.64	PASS	AC=1;AF=0.500;AN=2;BaseQRankSum=-2.820e-01;DP=9;ExcessHet=0.0000;FS=0.000;MLEAC=1;MLEAF=0.500;MQ=60.00;MQRankSum=0.00;QD=6.18;ReadPosRankSum=-2.820e-01;SOR=0.495	GT:AD:DP:GQ:PGT:PID:PL:PS	0|1:7,2:9:63:0|1:8255_G_A:63,0,288:8255	BA.5	no
8262	C	T	55.64	PA	AC=1;AF=0.500;AN=2;BaseQRankSum=-2.820e-	GT:A	0|1:7,2:9:63:	BA.5	no

				SS	01;DP=9;ExcessHet=0.0000;FS=0.000;MLEAC=1;MLEAF=0.500;MQ=60.00;MQRankSum=0.00;QD=6.18;ReadPosRankSum=-1.383e+00;SOR=0.495	D:DP:GQ:PGT:PID:PL:PS	0|1:8255_G_A:63,0,288:8255		
8289	T	A	78.32	PASS	AC=2;AF=1.00;AN=2;DP=2;ExcessHet=0.0000;FS=0.000;MLEAC=1;MLEAF=0.500;MQ=60.00;QD=28.20;SOR=0.693	GT:AD:DP:GQ:PGT:PID:PL:PS	1|1:0,2:2:6:1|1:8289_T_A:90,6,0:8289	BA.5	no
8292	C	T	78.32	PASS	AC=2;AF=1.00;AN=2;DP=2;ExcessHet=0.0000;FS=0.000;MLEAC=1;MLEAF=0.500;MQ=60.00;QD=25.00;SOR=0.693	GT:AD:DP:GQ:PGT:PID:PL:PS	1|1:0,2:2:6:1|1:8289_T_A:90,6,0:8289	BA.5	no
8295	A	C	78.32	PASS	AC=2;AF=1.00;AN=2;DP=2;ExcessHet=0.0000;FS=0.000;MLEAC=1;MLEAF=0.500;MQ=60.00;QD=29.56;SOR=0.693	GT:AD:DP:GQ:PGT:PID:PL:PS	1|1:0,2:2:6:1|1:8289_T_A:90,6,0:8289	BA.5	no
8463	C	A	166.14	PASS	AC=2;AF=1.00;AN=2;DP=4;ExcessHet=0.0000;FS=0.000;MLEAC=2;MLEAF=1.00;MQ=60.00;QD=33.63;SOR=0.693	GT:AD:DP:GQ:PGT:PID:	1|1:0,4:4:12:1|1:8452_ACGTAGTG_A:180,	BA.5	no

						PL:PS	12,0:8452		
8464	T	G	166.14	PASS	AC=2;AF=1.00;AN=2;DP=4;ExcessHet=0.0000;FS=0.000;MLEAC=2;MLEAF=1.00;MQ=60.00;QD=30.55;SOR=0.693	GT:AD:DP:GQ:PGT:PID:PL:PS	1|1:0,4:4:12:1|1:8452_ACGTAGTG_A:180,12,0:8452	BA.5	no
8592	T	G	163.64	PASS	AC=1;AF=0.500;AN=2;BaseQRankSum=3.03;DP=33;ExcessHet=0.0000;FS=4.263;MLEAC=1;MLEAF=0.500;MQ=60.00;MQRankSum=0.00;QD=4.96;ReadPosRankSum=-1.059e+00;SOR=1.187	GT:AD:DP:GQ:PGT:PID:PL:PS	0|1:27,6:33:99:0|1:8581_GTT_G:171,0,1116:8581	BA.5	no
8593	T	A	163.64	PASS	AC=1;AF=0.500;AN=2;BaseQRankSum=2.78;DP=33;ExcessHet=0.0000;FS=4.263;MLEAC=1;MLEAF=0.500;MQ=60.00;MQRankSum=0.00;QD=4.96;ReadPosRankSum=-1.059e+00;SOR=1.187	GT:AD:DP:GQ:PGT:PID:PL:PS	0|1:27,6:33:99:0|1:8581_GTT_G:171,0,1116:8581	BA.5	no
8598	T	A	157.64	PASS	AC=1;AF=0.500;AN=2;BaseQRankSum=2.92;DP=35;ExcessHet=0.0000;FS=4.212;MLEAC=1;MLEAF=0.500;MQ=60.00;MQRankSum=0.00;QD=4.50;ReadPosRankSum=-4.630e-01;SOR=1.194	GT:AD:DP:GQ:PGT:PID:PL:PS	0|1:29,6:35:99:0|1:8581_GTT_G:165,0,1200:8581	BA.5	no
8599	T	A	157.64	PASS	AC=1;AF=0.500;AN=2;BaseQRankSum=2.92;DP=35;ExcessHet=0.0000;FS=4.212;MLEAC=1;MLEAF=0.500;	GT:AD:DP:	0|1:29,6:35:99:0|1:8581_G	BA.5	no

					MQ=60.00;MQRankSum=0.00;QD=4.50;ReadPosRankSum=-8.160e-01;SOR=1.194	GQ:PGT:PID:PL:PS	TT_G:165,0,1200:8581		
8604	T	A	154.64	PASS	AC=1;AF=0.500;AN=2;BaseQRankSum=3.03;DP=36;ExcessHet=0.0000;FS=4.077;MLEAC=1;MLEAF=0.500;MQ=60.00;MQRankSum=0.00;QD=4.30;ReadPosRankSum=-1.081e+00;SOR=1.219	GT:AD:DP:GQ:PGT:PID:PL:PS	0|1:30,6:36:99:0|1:8581_GTT_G:162,0,1242:8581	BA.5	no
8605	C	A	154.64	PASS	AC=1;AF=0.500;AN=2;BaseQRankSum=3.05;DP=36;ExcessHet=0.0000;FS=4.077;MLEAC=1;MLEAF=0.500;MQ=60.00;MQRankSum=0.00;QD=4.30;ReadPosRankSum=-9.200e-01;SOR=1.219	GT:AD:DP:GQ:PGT:PID:PL:PS	0|1:30,6:36:99:0|1:8581_GTT_G:162,0,1242:8581	BA.5	no
8609	T	A	31.64	PASS	AC=1;AF=0.500;AN=2;BaseQRankSum=-6.740e-01;DP=2;ExcessHet=0.0000;FS=0.000;MLEAC=1;MLEAF=0.500;MQ=60.00;MQRankSum=0.00;QD=15.82;ReadPosRankSum=-6.740e-01;SOR=1.447	GT:AD:DP:GQ:PGT:PID:PL:PS	0|1:1,1:2:39:0|1:8605_CCTT_C:39,0,39:8605	BA.5	no
8614	T	G	31.64	PASS	AC=1;AF=0.500;AN=2;BaseQRankSum=-6.740e-01;DP=2;ExcessHet=0.0000;FS=0.000;MLEAC=1;MLEAF=0.500;MQ=60.00;MQRankSum=0.00;QD=15.82;ReadPosRankSum=-6.740e-01;SOR=1.447	GT:AD:DP:GQ:PGT:PID:PL:PS	0|1:1,1:2:39:0|1:8605_CCTT_C:39,0,39:8605	BA.5	no

8616	C	A	31.64	PASS	AC=1;AF=0.500;AN=2;BaseQRankSum=-6.740e-01;DP=2;ExcessHet=0.0000;FS=0.000;MLEAC=1;MLEAF=0.500;MQ=60.00;MQRankSum=0.00;QD=15.82;ReadPosRankSum=-6.740e-01;SOR=1.447	GT:AD:DP:GQ:PGT:PID:PL:PS	0|1:1,1:2:39:0|1:8605_CCTT_C:39,0,39:8605	BA.5	no
8626	C	T	202700.1	PASS	AC=2;AF=1.00;AN=2;BaseQRankSum=-2.572e+00;DP=5593;ExcessHet=0.0000;FS=0.000;MLEAC=2;MLEAF=1.00;MQ=60.00;MQRankSum=0.00;QD=28.17;ReadPosRankSum=-2.250e-01;SOR=0.816	GT:AD:DP:GQ:PL	1/1:21,5372:5393:99:202714,15262,0	BA.5	yes
8698	T	C	35.48	PASS	AC=2;AF=1.00;AN=2;DP=1;ExcessHet=0.0000;FS=0.000;MLEAC=1;MLEAF=0.500;MQ=60.00;QD=32.62;SOR=1.609	GT:AD:DP:GQ:PGT:PID:PL:PS	1|1:0,1:1:3:1|1:8698_T_C:45,3,0:8698	BA.5	no
8700	T	A	35.48	PASS	AC=2;AF=1.00;AN=2;DP=1;ExcessHet=0.0000;FS=0.000;MLEAC=1;MLEAF=0.500;MQ=60.00;QD=29.33;SOR=1.609	GT:AD:DP:GQ:PGT:PID:PL:PS	1|1:0,1:1:3:1|1:8698_T_C:45,3,0:8698	BA.5	no
8706	G	C	35.48	PASS	AC=2;AF=1.00;AN=2;DP=1;ExcessHet=0.0000;FS=0.000;MLEAC=1;MLEAF=0.500;MQ=60.00;QD=35.36;SOR=1.609	GT:AD:DP:GQ:PGT:PID:	1|1:0,1:1:3:1|1:8698_T_C:45,3,0:8698	BA.5	no

						PL:PS			
9130	G	T	26291.06	PASS	AC=2;AF=1.00;AN=2;DP=1060;ExcessHet=0.0000;FS=0.000;MLEAC=2;MLEAF=1.00;MQ=60.00;QD=28.12;SOR=0.907	GT:AD:DP:GQ:PL	1/1:0,935:935:99:26305,2804,0	BA.5	no
9160	T	A	1374.06	PASS	AC=2;AF=1.00;AN=2;DP=52;ExcessHet=0.0000;FS=0.000;MLEAC=2;MLEAF=1.00;MQ=60.00;QD=26.42;SOR=0.941	GT:AD:DP:GQ:PL	1/1:0,52:52:99:1388,156,0	BA.5	yes
9235	A	G	37.32	PASS	AC=2;AF=1.00;AN=2;DP=2;ExcessHet=0.0000;FS=0.000;MLEAC=1;MLEAF=0.500;MQ=60.00;QD=18.66;SOR=0.693	GT:AD:DP:GQ:PL	1/1:0,2:2:6:49,6,0	BA.5	no
9260	T	G	78.32	PASS	AC=2;AF=1.00;AN=2;DP=2;ExcessHet=0.0000;FS=0.000;MLEAC=1;MLEAF=0.500;MQ=60.00;QD=34.30;SOR=0.693	GT:AD:DP:GQ:PGT:PID:PL:PS	1|1:0,2:2:6:1|1:9260_T_G:90,6,0:9260	BA.5	no
9344	C	T	180600.1	PASS	AC=2;AF=1.00;AN=2;DP=4770;ExcessHet=0.0000;FS=0.000;MLEAC=2;MLEAF=1.00;MQ=60.00;QD=26.80;SOR=1.118	GT:AD:DP:GQ:PGT:PID:PL:PS	1|1:0,4649:4649:99:1|1:9344_C_T:180614,13977,0:9344	BA.5	yes
9406	A	G	78.32	PASS	AC=2;AF=1.00;AN=2;DP=2;ExcessHet=0.0000;FS=0.000;MLEAC=1;MLEAF=0.500;MQ=60.00;QD=35.92;SOR=0.693	GT:AD:DP:GQ:PG	1|1:0,2:2:6:1|1:9402_CTA_C:90,	BA.5	no

						T:PID:PL:PS	6,0:9402		
9414	G	A	78.32	PASS	AC=2;AF=1.00;AN=2;DP=2;ExcessHet=0.0000;FS=0.000;MLEAC=1;MLEAF=0.500;MQ=60.00;QD=29.94;SOR=0.693	GT:AD:DP:GQ:PGT:PID:PL:PS	1|1:0,2:2:6:1|1:9402_CTA_C:90,6,0:9402	BA.5	no
9417	G	T	73.64	PASS	AC=1;AF=0.500;AN=2;BaseQRankSum=-4.670e-01;DP=35;ExcessHet=0.0000;FS=0.000;MLEAC=1;MLEAF=0.500;MQ=60.00;MQRankSum=0.00;QD=2.23;ReadPosRankSum=-1.413e+00;SOR=0.631	GT:AD:DP:GQ:PGT:PID:PL:PS	0|1:29,4:33:81:0|1:9417_G_T:81,0,1206:9417	BA.5	no
9419	A	T	73.64	PASS	AC=1;AF=0.500;AN=2;BaseQRankSum=-1.880e+00;DP=33;ExcessHet=0.0000;FS=0.000;MLEAC=1;MLEAF=0.500;MQ=60.00;MQRankSum=0.00;QD=2.23;ReadPosRankSum=-1.411e+00;SOR=0.631	GT:AD:DP:GQ:PGT:PID:PL:PS	0|1:29,4:33:81:0|1:9417_G_T:81,0,1206:9417	BA.5	no
9420	T	G	73.64	PASS	AC=1;AF=0.500;AN=2;BaseQRankSum=-4.840e-01;DP=33;ExcessHet=0.0000;FS=0.000;MLEAC=1;MLEAF=0.500;MQ=60.00;MQRankSum=0.00;QD=2.23;ReadPosRankSum=-1.747e+00;SOR=0.631	GT:AD:DP:GQ:PGT:PID:PL:PS	0|1:29,4:33:81:0|1:9417_G_T:81,0,1206:9417	BA.5	no
9424	A	G	1741	PA	AC=2;AF=1.00;AN=2;BaseQRankSum=-8.300e-	GT:A	1|1:14,4451:4	BA.5	yes

			27.1	SS	01;DP=4659;ExcessHet=0.0000;FS=0.000;MLEAC=2;MLEAF=1.00;MQ=60.00;MQRankSum=0.00;QD=26.00;ReadPosRankSum=2.27;SOR=0.275	D:DP:GQ:PGT:PID:PL:PS	465:99:1|1:9344_C_T:174141,12818,0:9344		
9534	C	T	1833 98.1	PASS	AC=2;AF=1.00;AN=2;BaseQRankSum=-5.870e-01;DP=5088;ExcessHet=0.0000;FS=0.000;MLEAC=2;MLEAF=1.00;MQ=60.00;MQRankSum=0.524;QD=30.02;ReadPosRankSum=-1.486e+00;SOR=0.262	GT:AD:DP:GQ:PL	1/1:1,4898:4906:99:183412,14688,0	BA.5	yes
9535	T	A	35.48	PASS	AC=2;AF=1.00;AN=2;DP=1;ExcessHet=0.0000;FS=0.000;MLEAC=1;MLEAF=0.500;MQ=60.00;QD=28.86;SOR=1.609	GT:AD:DP:GQ:PGT:PID:PL:PS	1|1:0,1:1:3:1|1:9533_A_ATTG:45,3,0:9533	BA.5	no
9543	G	T	35.48	PASS	AC=2;AF=1.00;AN=2;DP=1;ExcessHet=0.0000;FS=0.000;MLEAC=1;MLEAF=0.500;MQ=60.00;QD=31.11;SOR=1.609	GT:AD:DP:GQ:PGT:PID:PL:PS	1|1:0,1:1:3:1|1:9533_A_ATTG:45,3,0:9533	BA.5	no
9545	T	A	35.48	PASS	AC=2;AF=1.00;AN=2;DP=1;ExcessHet=0.0000;FS=0.000;MLEAC=1;MLEAF=0.500;MQ=60.00;QD=34.66;SOR=1.609	GT:AD:DP:GQ:PGT:PID:PL:PS	1|1:0,1:1:3:1|1:9533_A_ATTG:45,3,0:9533	BA.5	no

9589	T	A	78.32	PASS	AC=2;AF=1.00;AN=2;DP=2;ExcessHet=0.0000;FS=0.000;MLEAC=1;MLEAF=0.500;MQ=60.00;QD=29.40;SOR=0.693	GT:AD:DP:GQ:PGT:PID:PL:PS	1|1:0,2:2:6:1|1:9589_T_A:90,6,0:9589	BA.5	no
9592	C	G	78.32	PASS	AC=2;AF=1.00;AN=2;DP=2;ExcessHet=0.0000;FS=0.000;MLEAC=1;MLEAF=0.500;MQ=60.00;QD=36.76;SOR=0.693	GT:AD:DP:GQ:PGT:PID:PL:PS	1|1:0,2:2:6:1|1:9589_T_A:90,6,0:9589	BA.5	no
9595	G	A	78.32	PASS	AC=2;AF=1.00;AN=2;DP=2;ExcessHet=0.0000;FS=0.000;MLEAC=1;MLEAF=0.500;MQ=60.00;QD=30.54;SOR=0.693	GT:AD:DP:GQ:PGT:PID:PL:PS	1|1:0,2:2:6:1|1:9589_T_A:90,6,0:9589	BA.5	no
9598	C	T	78.32	PASS	AC=2;AF=1.00;AN=2;DP=2;ExcessHet=0.0000;FS=0.000;MLEAC=1;MLEAF=0.500;MQ=60.00;QD=21.53;SOR=0.693	GT:AD:DP:GQ:PGT:PID:PL:PS	1|1:0,2:2:6:1|1:9589_T_A:90,6,0:9589	BA.5	yes
9599	T	A	78.32	PASS	AC=2;AF=1.00;AN=2;DP=2;ExcessHet=0.0000;FS=0.000;MLEAC=1;MLEAF=0.500;MQ=60.00;QD=34.87;SOR=0.693	GT:AD:DP:GQ:PG	1|1:0,2:2:6:1|1:9589_T_A:90,6,0:9589	BA.5	no

						T:PID:PL:PS			
9607	T	A	78.32	PASS	AC=2;AF=1.00;AN=2;DP=2;ExcessHet=0.0000;FS=0.000;MLEAC=1;MLEAF=0.500;MQ=60.00;QD=34.17;SOR=0.693	GT:AD:DP:GQ:PGT:PID:PL:PS	1|1:0,2:2:6:1|1:9589_T_A:90,6,0:9589	BA.5	no
10029	C	T	193634.1	PASS	AC=2;AF=1.00;AN=2;BaseQRankSum=-1.323e+00;DP=5359;ExcessHet=0.0000;FS=0.000;MLEAC=2;MLEAF=1.00;MQ=60.00;MQRankSum=0.00;QD=31.98;ReadPosRankSum=0.805;SOR=0.279	GT:AD:DP:GQ:PL	1/1:6,4814:4871:99:193648,14815,0	BA.5	yes
10198	C	T	217885.1	PASS	AC=2;AF=1.00;AN=2;BaseQRankSum=2.04;DP=5901;ExcessHet=0.0000;FS=0.000;MLEAC=2;MLEAF=1.00;MQ=60.00;MQRankSum=-3.340e-01;QD=27.51;ReadPosRankSum=4.00;SOR=0.722	GT:AD:DP:GQ:PL	1/1:6,5628:5644:99:217899,16732,0	BA.5	yes
10447	G	A	191966.1	PASS	AC=2;AF=1.00;AN=2;DP=4486;ExcessHet=0.0000;FS=0.000;MLEAC=2;MLEAF=1.00;MQ=60.00;QD=27.51;SOR=1.144	GT:AD:DP:GQ:PL	1/1:4,4287:4291:99:191980,12744,0	BA.5	yes
10449	C	A	240612.1	PASS	AC=2;AF=1.00;AN=2;DP=5512;ExcessHet=0.0000;FS=0.000;MLEAC=2;MLEAF=1.00;MQ=60.00;QD=28.08;SOR=1.141	GT:AD:DP:GQ:PL	1/1:0,4451:4530:99:240626,16153,0	BA.5	yes
10561	T	A	9727.64	PASS	AC=1;AF=0.500;AN=2;BaseQRankSum=0.328;DP=1792;ExcessHet=0.0000;FS=0.000;MLEAC=1;MLEAF=0.5	GT:AD:DP:	0/1:1198,519:1717:99:973	BA.5	no

					00;MQ=60.00;MQRankSum=0.00;QD=5.67;ReadPosRankSum=0.235;SOR=0.654	GQ:PL	5,0,29440		
10565	G	C	121.64	PASS	AC=1;AF=0.500;AN=2;BaseQRankSum=-9.420e-01;DP=17;ExcessHet=0.0000;FS=0.000;MLEAC=1;MLEAF=0.500;MQ=60.00;MQRankSum=0.00;QD=7.16;ReadPosRankSum=-2.001e+00;SOR=0.569	GT:AD:DP:GQ:PGT:PID:PL:PS	0|1:13,4:17:99:0|1:10482_GTTCATGTGGTAGTGTTGGTTTTAACATAGATTATGACTGTGTCTCTTTTTGTTACA_G:129,0,534:10482	BA.5	no
10568	C	G	121.64	PASS	AC=1;AF=0.500;AN=2;BaseQRankSum=-9.420e-01;DP=17;ExcessHet=0.0000;FS=0.000;MLEAC=1;MLEAF=0.500;MQ=60.00;MQRankSum=0.00;QD=7.16;ReadPosRankSum=-2.459e+00;SOR=0.569	GT:AD:DP:GQ:PGT:PID:PL:PS	0|1:13,4:17:99:0|1:10482_GTTCATGTGGTAGTGTTGGTTTTAACATAGATTATGACTGTGTCTCTTTTTGTTACA_G:129,0,5	BA.5	no

							34:10482		
10615	C	T	63156.06	PASS	AC=2;AF=1.00;AN=2;DP=2314;ExcessHet=0.0000;FS=0.000;MLEAC=2;MLEAF=1.00;MQ=60.00;QD=29.93;SOR=1.019	GT:AD:DP:GQ:PL	1/1:0,2110:2112:99:63170,6380,0	BA.5	yes
10745	A	T	61.64	PASS	AC=1;AF=0.500;AN=2;BaseQRankSum=-3.660e-01;DP=7;ExcessHet=0.0000;FS=0.000;MLEAC=1;MLEAF=0.500;MQ=60.00;MQRankSum=0.00;QD=8.81;ReadPosRankSum=-1.981e+00;SOR=0.446	GT:AD:DP:GQ:PGT:PID:PL:PS	0|1:5,2:7:69:0|1:10740_A_AGATCTTG:69,0,204:10740	BA.5	no
10746	A	C	61.64	PASS	AC=1;AF=0.500;AN=2;BaseQRankSum=-3.660e-01;DP=7;ExcessHet=0.0000;FS=0.000;MLEAC=1;MLEAF=0.500;MQ=60.00;MQRankSum=0.00;QD=8.81;ReadPosRankSum=-1.981e+00;SOR=0.446	GT:AD:DP:GQ:PGT:PID:PL:PS	0|1:5,2:7:69:0|1:10740_A_AGATCTTG:69,0,204:10740	BA.5	no
10809	C	T	4201.64	PASS	AC=1;AF=0.500;AN=2;BaseQRankSum=1.24;DP=270;ExcessHet=0.0000;FS=1.040;MLEAC=1;MLEAF=0.500;MQ=60.00;MQRankSum=0.00;QD=16.48;ReadPosRankSum=-8.770e-01;SOR=0.785	GT:AD:DP:GQ:PL	0/1:79,176:255:99:4209,0,1373	BA.5	no
10835	G	A	35.48	PASS	AC=2;AF=1.00;AN=2;DP=1;ExcessHet=0.0000;FS=0.000;MLEAC=1;MLEAF=0.500;MQ=60.00;QD=30.20;SOR=1.609	GT:AD:DP:GQ:PGT:PID:PL:PS	1|1:0,1:1:3:1|1:10834_C_CCAACAAT:45,3,0:10834	BA.5	no

11266	G	T	150.97	PASS	AC=2;AF=1.00;AN=2;DP=6;ExcessHet=0.0000;FS=0.000;MLEAC=2;MLEAF=1.00;MQ=60.00;QD=25.16;SOR=0.693	GT:AD:DP:GQ:PL	1/1:0,6:6:18:165,18,0	BA.5	yes
11381	A	G	35.48	PASS	AC=2;AF=1.00;AN=2;DP=1;ExcessHet=0.0000;FS=0.000;MLEAC=1;MLEAF=0.500;MQ=60.00;QD=31.67;SOR=1.609	GT:AD:DP:GQ:PGT:PID:PL:PS	1|1:0,1:1:3:1|1:11378_G_GA:45,3,0:11378	BA.5	no
11382	G	T	35.48	PASS	AC=2;AF=1.00;AN=2;DP=1;ExcessHet=0.0000;FS=0.000;MLEAC=1;MLEAF=0.500;MQ=60.00;QD=29.53;SOR=1.609	GT:AD:DP:GQ:PGT:PID:PL:PS	1|1:0,1:1:3:1|1:11378_G_GA:45,3,0:11378	BA.5	no
11410	G	C	70.64	PASS	AC=1;AF=0.500;AN=2;BaseQRankSum=1.38;DP=6;ExcessHet=0.0000;FS=0.000;MLEAC=1;MLEAF=0.500;MQ=60.00;MQRankSum=0.00;QD=17.66;ReadPosRankSum=-1.383e+00;SOR=2.303	GT:AD:DP:GQ:PGT:PID:PL:PS	0|1:2,2:4:78:0|1:11410_G_C:78,0,78:11410	BA.5	no
11779	C	A	35.48	PASS	AC=2;AF=1.00;AN=2;DP=1;ExcessHet=0.0000;FS=0.000;MLEAC=1;MLEAF=0.500;MQ=60.00;QD=27.97;SOR=1.609	GT:AD:DP:GQ:PGT:PID:PL:PS	1|1:0,1:1:3:1|1:11779_C_A:45,3,0:11779	BA.5	no

11784	T	G	35.48	PASS	AC=2;AF=1.00;AN=2;DP=1;ExcessHet=0.0000;FS=0.000;MLEAC=1;MLEAF=0.500;MQ=60.00;QD=35.21;SOR=1.609	GT:AD:DP:GQ:PGT:PID:PL:PS	1|1:0,1:1:3:1|1:11779_C_A:45,3,0:11779	BA.5	no
11804	G	T	55.64	PASS	AC=1;AF=0.500;AN=2;BaseQRankSum=0.319;DP=9;ExcessHet=0.0000;FS=0.000;MLEAC=1;MLEAF=0.500;MQ=60.00;MQRankSum=0.00;QD=6.18;ReadPosRankSum=-1.383e+00;SOR=0.495	GT:AD:DP:GQ:PGT:PID:PL:PS	0|1:7,2:9:63:0|1:11801_G_GTTAAACA:63,0,268:11801	BA.5	no
11824	C	T	174100.1	PASS	AC=2;AF=1.00;AN=2;BaseQRankSum=-2.339e+00;DP=4938;ExcessHet=0.0000;FS=0.000;MLEAC=2;MLEAF=1.00;MQ=60.00;MQRankSum=0.106;QD=23.23;ReadPosRankSum=4.61;SOR=0.232	GT:AD:DP:GQ:PL	1/1:15,4683:4698:99:174114,13488,0	BA.5	yes
11920	T	G	35.48	PASS	AC=2;AF=1.00;AN=2;DP=1;ExcessHet=0.0000;FS=0.000;MLEAC=1;MLEAF=0.500;MQ=60.00;QD=29.59;SOR=1.609	GT:AD:DP:GQ:PGT:PID:PL:PS	1|1:0,1:1:3:1|1:11920_T_G:45,3,0:11920	BA.5	no
12038	G	T	64.64	PASS	AC=1;AF=0.500;AN=2;BaseQRankSum=0.00;DP=6;ExcessHet=0.0000;FS=0.000;MLEAC=1;MLEAF=0.500;MQ=60.00;MQRankSum=0.00;QD=10.77;ReadPosRankSum=-1.834e+00;SOR=0.693	GT:AD:DP:GQ:PGT:PID:	0|1:4,2:6:72:0|1:12036_C_CATCA:72,0,162:12036	BA.5	no

						PL:PS			
12160	G	A	197696.1	PASS	AC=2;AF=1.00;AN=2;BaseQRankSum=1.96;DP=5451;ExcessHet=0.0000;FS=0.000;MLEAC=2;MLEAF=1.00;MQ=60.00;MQRankSum=0.00;QD=29.03;ReadPosRankSum=4.36;SOR=0.203	GT:AD:DP:GQ:PL	1/1:11,5256:5267:99:197710,15456,0	BA.5	yes
12258	A	C	35.48	PASS	AC=2;AF=1.00;AN=2;DP=1;ExcessHet=0.0000;FS=0.000;MLEAC=1;MLEAF=0.500;MQ=60.00;QD=34.86;SOR=1.609	GT:AD:DP:GQ:PL	1/1:0,1:1:3:45,3,0	BA.5	no
12310	G	A	205706.1	PASS	AC=2;AF=1.00;AN=2;BaseQRankSum=-1.296e+00;DP=5547;ExcessHet=0.0000;FS=0.000;MLEAC=2;MLEAF=1.00;MQ=60.00;MQRankSum=0.00;QD=29.03;ReadPosRankSum=0.707;SOR=0.465	GT:AD:DP:GQ:PL	1/1:4,5283:5291:99:205720,15768,0	BA.5	yes
12880	C	T	168981.1	PASS	AC=2;AF=1.00;AN=2;DP=5126;ExcessHet=0.0000;FS=0.000;MLEAC=2;MLEAF=1.00;MQ=60.00;QD=30.67;SOR=0.882	GT:AD:DP:GQ:PL	1/1:0,4603:4606:99:168995,13844,0	BA.5	yes
13698	T	A	70.64	PASS	AC=1;AF=0.500;AN=2;BaseQRankSum=0.00;DP=4;ExcessHet=0.0000;FS=0.000;MLEAC=1;MLEAF=0.500;MQ=60.00;MQRankSum=0.00;QD=17.66;ReadPosRankSum=1.38;SOR=0.693	GT:AD:DP:GQ:PGT:PID:PL:PS	0|1:2,2:4:78:0|1:13698_T_*:78,0,78:13698	BA.5	no
13700	A	C	70.64	PASS	AC=1;AF=0.500;AN=2;BaseQRankSum=0.00;DP=4;ExcessHet=0.0000;FS=0.000;MLEAC=1;MLEAF=0.500;MQ=60.00;MQRankSum=0.00;QD=17.66;ReadPosRankSum=1.38;SOR=0.693	GT:AD:DP:GQ:PGT:P	0|1:2,2:4:78:0|1:13698_T_*:78,0,78:13698	BA.5	no

						ID:PL:PS			
13708	C	A	70.64	PASS	AC=1;AF=0.500;AN=2;BaseQRankSum=0.00;DP=4;ExcessHet=0.0000;FS=0.000;MLEAC=1;MLEAF=0.500;MQ=60.00;MQRankSum=0.00;QD=17.66;ReadPosRankSum=1.38;SOR=0.693	GT:AD:DP:GQ:PGT:PID:PL:PS	0|1:2,2:4:78:0|1:13698_T_*:78,0,78:13698	BA.5	no
13791	A	C	37.32	PASS	AC=2;AF=1.00;AN=2;DP=2;ExcessHet=0.0000;FS=0.000;MLEAC=1;MLEAF=0.500;MQ=60.00;QD=18.66;SOR=0.693	GT:AD:DP:GQ:PL	1/1:0,2:2:6:49,6,0	BA.5	no
13876	C	A	78.32	PASS	AC=2;AF=1.00;AN=2;DP=2;ExcessHet=0.0000;FS=0.000;MLEAC=1;MLEAF=0.500;MQ=60.00;QD=28.53;SOR=0.693	GT:AD:DP:GQ:PGT:PID:PL:PS	1|1:0,2:2:6:1|1:13876_C_A:90,6,0:13876	BA.5	no
13878	T	C	78.32	PASS	AC=2;AF=1.00;AN=2;DP=2;ExcessHet=0.0000;FS=0.000;MLEAC=1;MLEAF=0.500;MQ=60.00;QD=27.23;SOR=0.693	GT:AD:DP:GQ:PGT:PID:PL:PS	1|1:0,2:2:6:1|1:13876_C_A:90,6,0:13876	BA.5	no
13879	G	A	78.32	PASS	AC=2;AF=1.00;AN=2;DP=2;ExcessHet=0.0000;FS=0.000;MLEAC=1;MLEAF=0.500;MQ=60.00;QD=29.52;SOR=0.693	GT:AD:DP:GQ:PGT:P	1|1:0,2:2:6:1|1:13876_C_A:90,6,0:13876	BA.5	no

						ID:PL:PS			
14181	G	C	3590.64	PASS	AC=1;AF=0.500;AN=2;BaseQRankSum=0.100;DP=654;ExcessHet=0.0000;FS=1.190;MLEAC=1;MLEAF=0.500;MQ=60.00;MQRankSum=0.00;QD=5.75;ReadPosRankSum=2.76;SOR=0.632	GT:AD:DP:GQ:PL	0/1:423,201:624:99:3598,0,9674	BA.5	no
14265	C	T	287003.06	PASS	AC=2;AF=1.00;AN=2;DP=1070;ExcessHet=0.0000;FS=0.000;MLEAC=2;MLEAF=1.00;MQ=60.00;QD=27.98;SOR=0.705	GT:AD:DP:GQ:PL	1/1:0,1026:1026:99:28717,3074,0	BA.5	yes
14359	A	G	61.64	PASS	AC=1;AF=0.500;AN=2;BaseQRankSum=0.524;DP=10;ExcessHet=0.0000;FS=0.000;MLEAC=1;MLEAF=0.500;MQ=60.00;MQRankSum=0.00;QD=6.16;ReadPosRankSum=0.859;SOR=0.693	GT:AD:DP:GQ:PL	0/1:6,4:10:69:69,0,104	BA.5	no
14395	A	G	681.64	PASS	AC=1;AF=0.500;AN=2;BaseQRankSum=-1.447e+00;DP=58;ExcessHet=0.0000;FS=1.022;MLEAC=1;MLEAF=0.500;MQ=60.00;MQRankSum=0.00;QD=11.96;ReadPosRankSum=0.073;SOR=0.495	GT:AD:DP:GQ:PGT:PID:PL:PS	0|1:25,32:57:99:0|1:14395_A_G:689,0,567:14395	BA.5	no
14408	C	T	1937 73.1	PASS	AC=2;AF=1.00;AN=2;BaseQRankSum=1.82;DP=5338;ExcessHet=0.0000;FS=0.000;MLEAC=2;MLEAF=1.00;MQ=60.00;MQRankSum=-5.940e-01;QD=26.61;ReadPosRankSum=0.703;SOR=0.297	GT:AD:DP:GQ:PL	1/1:1,5119:5120:99:193787,15363,0	BA.5	yes
14673	A	G	77.84	PASS	AC=2;AF=1.00;AN=2;DP=4;ExcessHet=0.0000;FS=0.000;MLEAC=1;MLEAF=0.500;MQ=60.00;QD=25.95;SOR=1.179	GT:AD:DP:	1/1:0,3:3:9:91,9,0	BA.5	yes

14874	G	A	31.64	PASS	AC=1;AF=0.500;AN=2;BaseQRankSum=0.00;DP=2;ExcessHet=0.0000;FS=0.000;MLEAC=1;MLEAF=0.500;MQ=60.00;MQRankSum=0.00;QD=15.82;ReadPosRankSum=-6.740e-01;SOR=0.693	GQ:PL GT:AD:DP:GQ:PGT:PID:PL:PS	0|1:1,1:2:39:0|1:14874_G_A:39,0,39:14874	BA.5	no
14877	C	T	31.64	PASS	AC=1;AF=0.500;AN=2;BaseQRankSum=0.00;DP=2;ExcessHet=0.0000;FS=0.000;MLEAC=1;MLEAF=0.500;MQ=60.00;MQRankSum=0.00;QD=15.82;ReadPosRankSum=-6.740e-01;SOR=0.693	GT:AD:DP:GQ:PGT:PID:PL:PS	0|1:1,1:2:39:0|1:14874_G_A:39,0,39:14874	BA.5	yes
14885	G	T	35.48	PASS	AC=2;AF=1.00;AN=2;DP=1;ExcessHet=0.0000;FS=0.000;MLEAC=1;MLEAF=0.500;MQ=60.00;QD=29.95;SOR=1.609	GT:AD:DP:GQ:PGT:PID:PL:PS	1|1:0,1:1:3:1|1:14883_T_TA:45,3,0:14883	BA.5	no
14889	C	T	35.48	PASS	AC=2;AF=1.00;AN=2;DP=1;ExcessHet=0.0000;FS=0.000;MLEAC=1;MLEAF=0.500;MQ=60.00;QD=30.22;SOR=1.609	GT:AD:DP:GQ:PGT:PID:PL:PS	1|1:0,1:1:3:1|1:14883_T_TA:45,3,0:14883	BA.5	no
15262	G	T	78.32	PASS	AC=2;AF=1.00;AN=2;DP=2;ExcessHet=0.0000;FS=0.000;MLEAC=1;MLEAF=0.5	GT:AD:DP:	1|1:0,2:2:6:1|1:15259_A_	BA.5	no

					00;MQ=60.00;QD=31.18;SOR=0.693	GQ:PGT:PID:PL:PS	ATAGGG:90,6,0:15259		
15263	A	T	78.32	PASS	AC=2;AF=1.00;AN=2;DP=2;ExcessHet=0.0000;FS=0.000;MLEAC=1;MLEAF=0.500;MQ=60.00;QD=28.65;SOR=0.693	GT:AD:DP:GQ:PGT:PID:PL:PS	1|1:0,2:2:6:1|1:15259_A_ATAGGG:90,6,0:15259	BA.5	no
15267	A	G	78.32	PASS	AC=2;AF=1.00;AN=2;DP=4;ExcessHet=0.0000;FS=0.000;MLEAC=1;MLEAF=0.500;MQ=60.00;QD=25.00;SOR=0.693	GT:AD:DP:GQ:PGT:PID:PL:PS	1|1:0,2:2:6:1|1:15267_A_G:90,6,0:15267	BA.5	no
15342	C	T	507753.06	PASS	AC=2;AF=1.00;AN=2;BaseQRankSum=2.04;DP=1953;ExcessHet=0.0000;FS=0.000;MLEAC=2;MLEAF=1.00;MQ=60.00;MQRankSum=0.00;QD=34.76;ReadPosRankSum=-4.700e-02;SOR=0.775	GT:AD:DP:GQ:PL	1/1:2,1458:1466:99:50767,5346,0	BA.5	no
15352	C	T	289916.06	PASS	AC=2;AF=1.00;AN=2;DP=1102;ExcessHet=0.0000;FS=0.000;MLEAC=2;MLEAF=1.00;MQ=60.00;QD=26.97;SOR=0.742	GT:AD:DP:GQ:PL	1/1:0,1072:1072:99:28930,3210,0	BA.5	no
15714	C	T	2067770.1	PASS	AC=2;AF=1.00;AN=2;BaseQRankSum=-5.320e-01;DP=5762;ExcessHet=0.0000;FS=0.000;MLEAC=2;MLEAF=1.00;MQ=60.00;MQRankSum=0.00;QD=29.20	GT:AD:DP:GQ:PL	1/1:1,3007:3092:99:206784,16493,0	BA.5	yes

					;ReadPosRankSum=1.05;SOR=0.467				
16377	G	A	148765.1	PASS	AC=2;AF=1.00;AN=2;BaseQRankSum=2.06;DP=4464;ExcessHet=0.0000;FS=0.000;MLEAC=2;MLEAF=1.00;MQ=60.00;MQRankSum=0.00;QD=30.62;ReadPosRankSum=1.72;SOR=0.371	GT:AD:DP:GQ:PL	1/1:1,3409:3435:99:148779,12615,0	BA.5	no
16456	T	C	167.97	PASS	AC=2;AF=1.00;AN=2;DP=6;ExcessHet=0.0000;FS=0.000;MLEAC=2;MLEAF=1.00;MQ=60.00;QD=28.00;SOR=0.693	GT:AD:DP:GQ:PL	1/1:0,6:6:18:182,18,0	BA.5	yes
16616	C	A	215143.1	PASS	AC=2;AF=1.00;AN=2;DP=5666;ExcessHet=0.0000;FS=0.000;MLEAC=2;MLEAF=1.00;MQ=60.00;QD=28.55;SOR=0.943	GT:AD:DP:GQ:PL	1/1:0,5381:5409:99:215157,16555,0	BA.5	yes
16887	C	T	225424.1	PASS	AC=2;AF=1.00;AN=2;BaseQRankSum=1.36;DP=5879;ExcessHet=0.0000;FS=0.000;MLEAC=2;MLEAF=1.00;MQ=60.00;MQRankSum=0.00;QD=34.42;ReadPosRankSum=4.16;SOR=0.201	GT:AD:DP:GQ:PL	1/1:9,4791:4807:99:225438,16819,0	BA.5	yes
17130	C	T	90090.06	PASS	AC=2;AF=1.00;AN=2;BaseQRankSum=0.294;DP=2982;ExcessHet=0.0000;FS=0.000;MLEAC=2;MLEAF=1.00;MQ=60.00;MQRankSum=0.00;QD=31.51;ReadPosRankSum=0.138;SOR=0.435	GT:AD:DP:GQ:PL	1/1:16,2843:2869:99:90104,7965,0	BA.5	yes
17186	T	C	58.64	PASS	AC=1;AF=0.500;AN=2;BaseQRankSum=0.319;DP=8;ExcessHet=0.0000;FS=0.000;MLEAC=1;MLEAF=0.500;MQ=60.00;MQRankSum=0.00;QD=7.33;ReadPosRankSum=2.10;SOR=0.693	GT:AD:DP:GQ:PGT:PID:PL:PS	0|1:6,2:8:66:0|1:17186_T_C:66,0,246:17186	BA.5	no

17208	T	C	226869.1	PASS	AC=2;AF=1.00;AN=2;BaseQRankSum=2.34;DP=5909;ExcessHet=0.0000;FS=0.000;MLEAC=2;MLEAF=1.00;MQ=60.00;MQRankSum=0.00;QD=28.53;ReadPosRankSum=1.03;SOR=0.635	GT:AD:DP:GQ:PL	1/1:5,5746:5751:99:226883,17107,0	BA.5	yes
17410	C	T	207996.1	PASS	AC=2;AF=1.00;AN=2;DP=5545;ExcessHet=0.0000;FS=0.000;MLEAC=2;MLEAF=1.00;MQ=60.00;QD=27.23;SOR=1.142	GT:AD:DP:GQ:PL	1/1:0,5301:5305:99:208010,15974,0	BA.5	yes
17746	C	T	83132.06	PASS	AC=2;AF=1.00;AN=2;DP=2803;ExcessHet=0.0000;FS=0.000;MLEAC=2;MLEAF=1.00;MQ=60.00;QD=30.61;SOR=0.972	GT:AD:DP:GQ:PL	1/1:0,2716:2716:99:83146,8148,0	BA.5	yes
18160	G	A	75141.06	PASS	AC=2;AF=1.00;AN=2;DP=1801;ExcessHet=0.0000;FS=0.000;MLEAC=2;MLEAF=1.00;MQ=60.00;QD=29.56;SOR=0.838	GT:AD:DP:GQ:PL	1/1:0,1722:1740:99:75155,5190,0	BA.5	yes
18163	A	G	189840.1	PASS	AC=2;AF=1.00;AN=2;BaseQRankSum=-1.072e+00;DP=5361;ExcessHet=0.0000;FS=0.000;MLEAC=2;MLEAF=1.00;MQ=60.00;MQRankSum=0.00;QD=29.52;ReadPosRankSum=3.28;SOR=0.947	GT:AD:DP:GQ:PL	1/1:4,4983:4987:99:189854,14854,0	BA.5	yes
18206	C	A	35.48	PASS	AC=2;AF=1.00;AN=2;DP=1;ExcessHet=0.0000;FS=0.000;MLEAC=1;MLEAF=0.500;MQ=60.00;QD=35.95;SOR=1.609	GT:AD:DP:GQ:PGT:PID:PL:PS	1|1:0,1:1:3:1|1:18204_C_CAGCTTT:45,3,0:18204	BA.5	no
18261	C	T	47.64	PA	AC=1;AF=0.500;AN=2;BaseQRankSum=0.674;DP=12;ExcessHet=0.0000;FS=0.00	GT:AD:	0/1:9,3:12:5	BA.5	no

				SS	0;MLEAC=1;MLEAF=0.500;MQ=60.00;MQRankSum=0.00;QD=3.97;ReadPosRankSum=-2.278e+00;SOR=0.941	DP:GQ:PL	5:55,0,204		
18266	G	T	15990.06	PASS	AC=2;AF=1.00;AN=2;DP=601;ExcessHet=0.0000;FS=0.000;MLEAC=2;MLEAF=1.00;MQ=60.00;QD=27.71;SOR=0.739	GT:AD:DP:GQ:PL	1/1:0,577:577:99:16004,1730,0	BA.5	no
18574	G	A	37.32	PASS	AC=2;AF=1.00;AN=2;DP=2;ExcessHet=0.0000;FS=0.000;MLEAC=1;MLEAF=0.500;MQ=60.00;QD=18.66;SOR=0.693	GT:AD:DP:GQ:PL	1/1:0,2:2:6:49,6,0	BA.5	no
18686	G	C	43.64	PASS	AC=1;AF=0.500;AN=2;BaseQRankSum=0.00;DP=8;ExcessHet=0.0000;FS=3.332;MLEAC=1;MLEAF=0.500;MQ=60.00;MQRankSum=0.00;QD=5.45;ReadPosRankSum=0.272;SOR=2.303	GT:AD:DP:GQ:PL	0/1:5,3:8:51:51,0,102	BA.5	no
19018	C	T	51280.06	PASS	AC=2;AF=1.00;AN=2;BaseQRankSum=-1.903e+00;DP=1843;ExcessHet=0.0000;FS=0.000;MLEAC=2;MLEAF=1.00;MQ=60.00;MQRankSum=0.00;QD=28.60;ReadPosRankSum=-1.644e+00;SOR=0.303	GT:AD:DP:GQ:PL	1/1:1,1792:1793:99:51294,5336,0	BA.5	no
19046	A	T	52.64	PASS	AC=1;AF=0.500;AN=2;BaseQRankSum=0.00;DP=10;ExcessHet=0.0000;FS=0.000;MLEAC=1;MLEAF=0.500;MQ=60.00;MQRankSum=0.00;QD=5.26;ReadPosRankSum=-2.287e+00;SOR=0.693	GT:AD:DP:GQ:PGT:PID:PL:PS	0|1:8,2:10:60:0|1:19042_C_CGG:60,0,330:19042	BA.5	no
19596	C	T	8165.64	PASS	AC=1;AF=0.500;AN=2;BaseQRankSum=0.622;DP=1208;ExcessHet=0.0000;FS=1.152;MLEAC=1;MLEAF=0.5	GT:AD:DP:	0/1:695,388:1083:99:8173	BA.5	no

					00;MQ=60.00;MQRankSum=0.00;QD=7.54;ReadPosRankSum=2.85;SOR=0.711	GQ:PL	,0,16923		
19890	A	G	37.32	PASS	AC=2;AF=1.00;AN=2;DP=2;ExcessHet=0.0000;FS=0.000;MLEAC=1;MLEAF=0.500;MQ=60.00;QD=18.66;SOR=0.693	GT:AD:DP:GQ:PL	1/1:0,2:2:6:49,6,0	BA.5	no
19955	C	T	219706.1	PASS	AC=2;AF=1.00;AN=2;DP=5728;ExcessHet=0.0000;FS=0.000;MLEAC=2;MLEAF=1.00;MQ=60.00;QD=33.47;SOR=0.879	GT:AD:DP:GQ:PL	1/1:0,5619:5619:99:219720,16896,0	BA.5	yes
20055	A	G	198885.1	PASS	AC=2;AF=1.00;AN=2;BaseQRankSum=0.272;DP=5685;ExcessHet=0.0000;FS=0.000;MLEAC=2;MLEAF=1.00;MQ=60.00;MQRankSum=0.089;QD=32.91;ReadPosRankSum=-5.190e-01;SOR=0.416	GT:AD:DP:GQ:PL	1/1:4,4322:4326:99:198899,15478,0	BA.5	yes
20456	T	A	35.48	PASS	AC=2;AF=1.00;AN=2;DP=1;ExcessHet=0.0000;FS=0.000;MLEAC=1;MLEAF=0.500;MQ=60.00;QD=33.53;SOR=1.609	GT:AD:DP:GQ:PGT:PID:PL:PS	1|1:0,1:1:3:1|1:20456_T_A:45,3,0:20456	BA.5	no
20457	C	T	77.84	PASS	AC=2;AF=1.00;AN=2;DP=3;ExcessHet=0.0000;FS=0.000;MLEAC=1;MLEAF=0.500;MQ=60.00;QD=25.95;SOR=1.179	GT:AD:DP:GQ:PL	1/1:0,3:3:9:91,9,0	BA.5	no
20459	T	A	35.48	PASS	AC=2;AF=1.00;AN=2;DP=1;ExcessHet=0.0000;FS=0.000;MLEAC=1;MLEAF=0.500;MQ=60.00;QD=36.33;SOR=1.609	GT:AD:DP:GQ:PGT:P	1|1:0,1:1:3:1|1:20456_T_A:45,3,0:20456	BA.5	no

						ID:PL:PS			
20464	G	A	35.48	PASS	AC=2;AF=1.00;AN=2;DP=1;ExcessHet=0.0000;FS=0.000;MLEAC=1;MLEAF=0.500;MQ=60.00;QD=32.31;SOR=1.609	GT:AD:DP:GQ:PGT:PID:PL:PS	1|1:0,1:1:3:1|1:20456_T_A:45,3,0:20456	BA.5	no
20762	C	T	198730.1	PASS	AC=2;AF=1.00;AN=2;DP=5491;ExcessHet=0.0000;FS=0.000;MLEAC=2;MLEAF=1.00;MQ=60.00;QD=31.31;SOR=1.045	GT:AD:DP:GQ:PL	1/1:0,5300:5349:99:198744,15931,0	BA.5	yes
21156	C	A	35.48	PASS	AC=2;AF=1.00;AN=2;DP=1;ExcessHet=0.0000;FS=0.000;MLEAC=1;MLEAF=0.500;MQ=60.00;QD=34.29;SOR=1.609	GT:AD:DP:GQ:PGT:PID:PL:PS	1|1:0,1:1:3:1|1:21156_C_A:45,3,0:21156	BA.5	no
21159	G	A	35.48	PASS	AC=2;AF=1.00;AN=2;DP=1;ExcessHet=0.0000;FS=0.000;MLEAC=1;MLEAF=0.500;MQ=60.00;QD=30.05;SOR=1.609	GT:AD:DP:GQ:PGT:PID:PL:PS	1|1:0,1:1:3:1|1:21156_C_A:45,3,0:21156	BA.5	no
21161	C	G	35.48	PASS	AC=2;AF=1.00;AN=2;DP=1;ExcessHet=0.0000;FS=0.000;MLEAC=1;MLEAF=0.500;MQ=60.00;QD=33.30;SOR=1.609	GT:AD:DP:GQ:PGT:P	1|1:0,1:1:3:1|1:21156_C_A:45,3,0:21156	BA.5	no

						ID:PL:PS			
21306	C	A	3547.64	PASS	AC=1;AF=0.500;AN=2;BaseQRankSum=-4.550e-01;DP=336;ExcessHet=0.0000;FS=0.877;MLEAC=1;MLEAF=0.500;MQ=60.00;MQRankSum=0.00;QD=11.67;ReadPosRankSum=-1.092e+00;SOR=0.639	GT:AD:DP:GQ:PL	0/1:145,159:304:99:3555,0,3175	BA.5	no
21536	A	C	31.64	PASS	AC=1;AF=0.500;AN=2;BaseQRankSum=-6.740e-01;DP=2;ExcessHet=0.0000;FS=0.000;MLEAC=1;MLEAF=0.500;MQ=60.00;MQRankSum=0.00;QD=15.82;ReadPosRankSum=-6.740e-01;SOR=0.693	GT:AD:DP:GQ:PGT:PID:PL:PS	0|1:1,1:2:39:0|1:21536_A_C:39,0,39:21536	BA.5	no
21538	G	C	31.64	PASS	AC=1;AF=0.500;AN=2;BaseQRankSum=-6.740e-01;DP=2;ExcessHet=0.0000;FS=0.000;MLEAC=1;MLEAF=0.500;MQ=60.00;MQRankSum=0.00;QD=15.82;ReadPosRankSum=-6.740e-01;SOR=0.693	GT:AD:DP:GQ:PGT:PID:PL:PS	0|1:1,1:2:39:0|1:21536_A_C:39,0,39:21536	BA.5	no
21575	C	T	49316.06	PASS	AC=2;AF=1.00;AN=2;DP=1377;ExcessHet=0.0000;FS=0.000;MLEAC=2;MLEAF=1.00;MQ=60.00;QD=28.20;SOR=0.850	GT:AD:DP:GQ:PGT:PID:PL:PS	1|1:0,1356:1356:99:1|1:21575_C_T:49330,4073,0:21575	BA.5	yes
21618	C	T	174015.1	PASS	AC=2;AF=1.00;AN=2;DP=4174;ExcessHet=0.0000;FS=0.000;MLEAC=2;MLEAF=1.00;MQ=60.00;QD=31.45;SOR=0.973	GT:AD:DP:	1/1:0,4070:4073:99:17402	BA.5	yes

<table>
<tr><td></td><td></td><td></td><td></td><td></td><td></td><td>GQ:PL</td><td>9,12267,0</td><td></td><td></td></tr>
<tr><td>21711</td><td>C</td><td>T</td><td>66117.64</td><td>PASS</td><td>AC=1;AF=0.500;AN=2;BaseQRankSum=0.076;DP=3288;ExcessHet=0.0000;FS=1.868;MLEAC=1;MLEAF=0.500;MQ=60.00;MQRankSum=0.00;QD=21.05;ReadPosRankSum=2.97;SOR=0.742</td><td>GT:AD:DP:GQ:PL</td><td>0/1:1016,2125:3141:99:66125,0,26661</td><td>BA.5</td><td>no</td></tr>
<tr><td>21908</td><td>T</td><td>C</td><td>78.32</td><td>PASS</td><td>AC=2;AF=1.00;AN=2;DP=2;ExcessHet=0.0000;FS=0.000;MLEAC=1;MLEAF=0.500;MQ=60.00;QD=28.17;SOR=0.693</td><td>GT:AD:DP:GQ:PGT:PID:PL:PS</td><td>1|1:0,2:2:6:1|1:21908_T_C:90,6,0:21908</td><td>BA.5</td><td>no</td></tr>
<tr><td>21910</td><td>C</td><td>T</td><td>78.32</td><td>PASS</td><td>AC=2;AF=1.00;AN=2;DP=2;ExcessHet=0.0000;FS=0.000;MLEAC=1;MLEAF=0.500;MQ=60.00;QD=26.80;SOR=0.693</td><td>GT:AD:DP:GQ:PGT:PID:PL:PS</td><td>1|1:0,2:2:6:1|1:21908_T_C:90,6,0:21908</td><td>BA.5</td><td>no</td></tr>
<tr><td>21913</td><td>A</td><td>T</td><td>70.64</td><td>PASS</td><td>AC=1;AF=0.500;AN=2;BaseQRankSum=0.00;DP=4;ExcessHet=0.0000;FS=0.000;MLEAC=1;MLEAF=0.500;MQ=60.00;MQRankSum=0.00;QD=17.66;ReadPosRankSum=1.38;SOR=0.693</td><td>GT:AD:DP:GQ:PGT:PID:PL:PS</td><td>0|1:2,2:4:68:0|1:21908_T_C:78,0,68:21908</td><td>BA.5</td><td>no</td></tr>
<tr><td>21914</td><td>C</td><td>A</td><td>70.64</td><td>PASS</td><td>AC=1;AF=0.500;AN=2;BaseQRankSum=0.00;DP=4;ExcessHet=0.0000;FS=0.000;MLEAC=1;MLEAF=0.500;MQ=60.00;MQRankSum=0.00;QD=17.66;ReadPosRankSum=1.38;SOR=0.693</td><td>GT:AD:DP:GQ:PGT:P</td><td>0|1:2,2:4:68:0|1:21908_T_C:78,0,68:21908</td><td>BA.5</td><td>no</td></tr>
</table>

						ID:PL:PS			
21987	G	A	208544.1	PASS	AC=2;AF=1.00;AN=2;DP=5743;ExcessHet=0.0000;FS=0.000;MLEAC=2;MLEAF=1.00;MQ=60.00;MQRankSum=-7.850e-01;QD=27.76;SOR=0.593	GT:AD:DP:GQ:PL	1/1:1,5539:5540:99:208558,16646,0	BA.5	yes
22200	T	G	204588.1	PASS	AC=2;AF=1.00;AN=2;BaseQRankSum=4.86;DP=5704;ExcessHet=0.0000;FS=0.000;MLEAC=2;MLEAF=1.00;MQ=60.00;MQRankSum=0.00;QD=29.09;ReadPosRankSum=1.01;SOR=0.592	GT:AD:DP:GQ:PL	1/1:5,5526:5531:99:204602,16529,0	BA.5	yes
22480	C	T	236696.06	PASS	AC=2;AF=1.00;AN=2;DP=902;ExcessHet=0.0000;FS=0.000;MLEAC=2;MLEAF=1.00;MQ=60.00;QD=27.68;SOR=0.707	GT:AD:DP:GQ:PL	1/1:0,856:856:99:23710,2565,0	BA.5	yes
22578	G	A	156759.1	PASS	AC=2;AF=1.00;AN=2;BaseQRankSum=-7.220e-01;DP=4295;ExcessHet=0.0000;FS=0.000;MLEAC=2;MLEAF=1.00;MQ=60.00;MQRankSum=0.024;QD=26.08;ReadPosRankSum=3.94;SOR=0.490	GT:AD:DP:GQ:PL	1/1:16,4210:4226:99:156773,12016,0	BA.5	yes
22599	G	C	11326.64	PASS	AC=1;AF=0.500;AN=2;BaseQRankSum=1.56;DP=922;ExcessHet=0.0000;FS=0.000;MLEAC=1;MLEAF=0.500;MQ=60.00;MQRankSum=0.00;QD=12.73;ReadPosRankSum=-1.241e+00;SOR=0.696	GT:AD:DP:GQ:PL	0/1:386,504:890:99:11334,0,7946	BA.5	yes
22674	C	T	155955.1	PASS	AC=2;AF=1.00;AN=2;BaseQRankSum=-6.360e-01;DP=3682;ExcessHet=0.0000;FS=0.000;MLEAC=2;MLEAF=1.00;MQ=60.00;MQRankSum=0.00;QD=33.85;ReadPosRankSum=1.70;SOR=0.318	GT:AD:DP:GQ:PGT:PID:	1|1:1,3497:3498:99:1|1:22674_C_T:155969,1	BA.5	yes

						PL:PS	0484,0:22674		
22679	T	C	152384.1	PASS	AC=2;AF=1.00;AN=2;DP=3393;ExcessHet=0.0000;FS=0.000;MLEAC=2;MLEAF=1.00;MQ=60.00;QD=27.08;SOR=0.891	GT:AD:DP:GQ:PGT:PID:PL:PS	1|1:0,3393:3393:99:1|1:22674_C_T:152398,10213,0:22674	BA.5	yes
22683	T	A	305.64	PASS	AC=1;AF=0.500;AN=2;BaseQRankSum=-6.730e-01;DP=69;ExcessHet=0.0000;FS=1.061;MLEAC=1;MLEAF=0.500;MQ=60.00;MQRankSum=0.00;QD=4.43;ReadPosRankSum=2.60;SOR=0.532	GT:AD:DP:GQ:PL	0/1:51,18:69:99:313,0,1355	BA.5	no
22686	C	T	146518.1	PASS	AC=2;AF=1.00;AN=2;BaseQRankSum=1.61;DP=3260;ExcessHet=0.0000;FS=0.000;MLEAC=2;MLEAF=1.00;MQ=60.00;MQRankSum=0.00;QD=25.42;ReadPosRankSum=1.35;SOR=0.162	GT:AD:DP:GQ:PGT:PID:PL:PS	1|1:2,3258:3260:99:1|1:22674_C_T:146532,9756,0:22674	BA.5	yes
22688	A	G	142916.1	PASS	AC=2;AF=1.00;AN=2;BaseQRankSum=2.06;DP=3194;ExcessHet=0.0000;FS=0.000;MLEAC=2;MLEAF=1.00;MQ=60.00;MQRankSum=0.00;QD=27.65;ReadPosRankSum=1.93;SOR=0.176	GT:AD:DP:GQ:PL	1/1:2,3128:3134:99:142930,9518,0	BA.5	yes
22775	G	A	109433.1	PASS	AC=2;AF=1.00;AN=2;BaseQRankSum=1.09;DP=3179;ExcessHet=0.0000;FS=0.000;MLEAC=2;MLEAF=1.00;MQ=60.00;MQRankSum=0.00;QD=27.08;ReadPosRankSum=2.08;SOR=0.456	GT:AD:DP:GQ:PL	1/1:26,2987:3013:99:109447,8245,0	BA.5	yes

22776	A	G	17805.64	PASS	AC=1;AF=0.500;AN=2;BaseQRankSum=7.25;DP=2222;ExcessHet=0.0000;FS=1.853;MLEAC=1;MLEAF=0.500;MQ=60.00;MQRankSum=0.00;QD=8.43;ReadPosRankSum=10.34;SOR=0.852	GT:AD:DP:GQ:PL	0/1:1415,698:2126:99:17813,0,39342	BA.5	no
22786	A	C	130133.1	PASS	AC=2;AF=1.00;AN=2;BaseQRankSum=2.83;DP=3063;ExcessHet=0.0000;FS=0.000;MLEAC=2;MLEAF=1.00;MQ=60.00;MQRankSum=0.00;QD=25.42;ReadPosRankSum=0.012;SOR=0.108	GT:AD:DP:GQ:PL	1/1:2,2995:2997:99:130147,8987,0	BA.5	yes
22813	G	T	156291.1	PASS	AC=2;AF=1.00;AN=2;DP=3544;ExcessHet=0.0000;FS=0.000;MLEAC=2;MLEAF=1.00;MQ=60.00;QD=27.65;SOR=0.739	GT:AD:DP:GQ:PL	1/1:0,3530:3530:99:156305,10620,0	BA.5	yes
22882	T	G	184891.1	PASS	AC=2;AF=1.00;AN=2;DP=4220;ExcessHet=0.0000;FS=0.000;MLEAC=2;MLEAF=1.00;MQ=60.00;QD=29.48;SOR=0.833	GT:AD:DP:GQ:PL	1/1:0,4205:4205:99:184905,12652,0	BA.5	yes
22917	T	G	188641.1	PASS	AC=2;AF=1.00;AN=2;BaseQRankSum=2.99;DP=4634;ExcessHet=0.0000;FS=0.000;MLEAC=2;MLEAF=1.00;MQ=60.00;MQRankSum=2.000e-03;QD=30.56;ReadPosRankSum=1.50;SOR=0.140	GT:AD:DP:GQ:PL	1/1:2,4052:4102:99:188655,13568,0	BA.5	yes
22973	G	C	506.06	PASS	AC=2;AF=1.00;AN=2;DP=13;ExcessHet=0.0000;FS=0.000;MLEAC=2;MLEAF=1.00;MQ=60.00;QD=30.62;SOR=0.836	GT:AD:DP:GQ:PGT:PID:PL:PS	1|1:0,13:13:39:1|1:22973_G_C:520,39,0:22973	BA.5	no

22992	G	A	152522.1	PASS	AC=2;AF=1.00;AN=2;BaseQRankSum=-5.170e-01;DP=4218;ExcessHet=0.0000;FS=0.000;MLEAC=2;MLEAF=1.00;MQ=60.00;MQRankSum=0.00;QD=33.63;ReadPosRankSum=-3.970e-01;SOR=0.415	GT:AD:DP:GQ:PL	1/1:1,3570:3575:99:152536,10780,0	BA.5	yes
22995	C	A	148879.1	PASS	AC=2;AF=1.00;AN=2;DP=3322;ExcessHet=0.0000;FS=0.000;MLEAC=2;MLEAF=1.00;MQ=60.00;QD=29.52;SOR=1.078	GT:AD:DP:GQ:PL	1/1:0,2901:2902:99:148893,9997,0	BA.5	yes
23013	A	C	153624.1	PASS	AC=2;AF=1.00;AN=2;BaseQRankSum=-1.794e+00;DP=3475;ExcessHet=0.0000;FS=0.000;MLEAC=2;MLEAF=1.00;MQ=60.00;MQRankSum=0.00;QD=32.62;ReadPosRankSum=0.934;SOR=0.509	GT:AD:DP:GQ:PL	1/1:15,3455:3470:99:153638,10093,0	BA.5	yes
23018	T	G	132847.1	PASS	AC=2;AF=1.00;AN=2;DP=3256;ExcessHet=0.0000;FS=0.000;MLEAC=2;MLEAF=1.00;MQ=60.00;QD=29.33;SOR=1.011	GT:AD:DP:GQ:PL	1/1:0,3028:3028:99:132861,9192,0	BA.5	yes
23055	A	G	112012.1	PASS	AC=2;AF=1.00;AN=2;BaseQRankSum=-2.387e+00;DP=2556;ExcessHet=0.0000;FS=0.000;MLEAC=2;MLEAF=1.00;MQ=60.00;MQRankSum=0.00;QD=35.36;ReadPosRankSum=-1.310e-01;SOR=0.025	GT:AD:DP:GQ:PGT:PID:PL:PS	1|1:32,2523:2555:99:1|1:23055_A_G:112026,6306,0:23055	BA.5	yes
23063	A	T	107511.1	PASS	AC=2;AF=1.00;AN=2;BaseQRankSum=-3.064e+00;DP=2465;ExcessHet=0.0000;FS=0.000;MLEAC=2;MLEAF=1.00;MQ=60.00;MQRankSum=0.00;QD=34.30;ReadPosRankSum=-6.910e-01;SOR=0.276	GT:AD:DP:GQ:PGT:PID:	1|1:31,2434:2465:99:1|1:23055_A_G:107525,6	BA.5	yes

						PL:PS	033,0:23055		
23075	T	C	103540.1	PASS	AC=2;AF=1.00;AN=2;BaseQRankSum=-8.300e-01;DP=2451;ExcessHet=0.0000;FS=0.000;MLEAC=2;MLEAF=1.00;MQ=60.00;MQRankSum=0.00;QD=27.00;ReadPosRankSum=0.629;SOR=0.102	GT:AD:DP:GQ:PGT:PID:PL:PS	1|1:3,2346:2349:99:1|1:23055_A_G:103554,7078,0:23055	BA.5	yes
23084	T	C	86.64	PASS	AC=1;AF=0.500;AN=2;BaseQRankSum=1.82;DP=42;ExcessHet=0.0000;FS=0.000;MLEAC=1;MLEAF=0.500;MQ=60.00;MQRankSum=0.00;QD=2.11;ReadPosRankSum=0.742;SOR=0.641	GT:AD:DP:GQ:PL	0/1:35,6:41:94:94,0,912	BA.5	no
23156	A	G	37.32	PASS	AC=2;AF=1.00;AN=2;DP=2;ExcessHet=0.0000;FS=0.000;MLEAC=1;MLEAF=0.500;MQ=60.00;QD=18.66;SOR=0.693	GT:AD:DP:GQ:PL	1/1:0,2:2:6:49,6,0	BA.5	no
23191	C	T	62593.06	PASS	AC=2;AF=1.00;AN=2;DP=2120;ExcessHet=0.0000;FS=0.000;MLEAC=2;MLEAF=1.00;MQ=60.00;QD=30.30;SOR=0.784	GT:AD:DP:GQ:PL	1/1:0,2066:2074:99:62607,6198,0	BA.5	yes
23270	G	T	226.05	PASS	AC=2;AF=1.00;AN=2;DP=9;ExcessHet=0.0000;FS=0.000;MLEAC=2;MLEAF=1.00;MQ=60.00;QD=25.12;SOR=0.892	GT:AD:DP:GQ:PL	1/1:0,9:9:27:240,27,0	BA.5	yes
23315	C	T	43.64	PASS	AC=1;AF=0.500;AN=2;BaseQRankSum=1.92;DP=15;ExcessHet=0.0000;FS=3.424;MLEAC=1;MLEAF=0.500;MQ=60.00;MQRankSum=0.00;QD=3.97;ReadPosRankSum=1.10;SOR=2.272	GT:AD:DP:GQ:PL	0/1:9,2:11:51:51,0,206	BA.5	no

23403	A	G	2259 95.1	PASS	AC=2;AF=1.00;AN=2;DP=5929;ExcessHet=0.0000;FS=0.000;MLEAC=2;MLEAF=1.00;MQ=60.00;QD=29.48;SOR=0.828	GT:AD:DP:GQ:PL	1/1:0,5754:5754:99:226009,17305,0	BA.5	yes
23520	C	T	1237 29.1	PASS	AC=2;AF=1.00;AN=2;BaseQRankSum=2.44;DP=3657;ExcessHet=0.0000;FS=0.000;MLEAC=2;MLEAF=1.00;MQ=60.00;MQRankSum=0.00;QD=34.96;ReadPosRankSum=1.34;SOR=0.702	GT:AD:DP:GQ:PL	1/1:18,3521:3539:99:123743,10093,0	BA.5	no
23525	C	T	2194 52.1	PASS	AC=2;AF=1.00;AN=2;BaseQRankSum=-4.170e-01;DP=5794;ExcessHet=0.0000;FS=0.000;MLEAC=2;MLEAF=1.00;MQ=60.00;MQRankSum=0.00;QD=30.56;ReadPosRankSum=1.26;SOR=0.474	GT:AD:DP:GQ:PL	1/1:1,3904:3911:99:219466,16893,0	BA.5	yes
23599	T	G	2213 46.1	PASS	AC=2;AF=1.00;AN=2;DP=4975;ExcessHet=0.0000;FS=0.000;MLEAC=2;MLEAF=1.00;MQ=60.00;QD=35.92;SOR=1.243	GT:AD:DP:GQ:PGT:PID:PL:PS	1|1:0,4957:4957:99:1|1:23525_C_T:221360,14921,0:23525	BA.5	yes
23604	C	A	2140 33.1	PASS	AC=2;AF=1.00;AN=2;DP=4865;ExcessHet=0.0000;FS=0.000;MLEAC=2;MLEAF=1.00;MQ=60.00;QD=29.63;SOR=1.232	GT:AD:DP:GQ:PL	1/1:0,4742:4743:99:214047,14435,0	BA.5	yes
23854	C	A	1249 08.1	PASS	AC=2;AF=1.00;AN=2;BaseQRankSum=-1.835e+00;DP=3997;ExcessHet=0.0000;FS=0.000;MLEAC=2;MLEAF=1.00;MQ=60.00;MQRankSum=0.00;QD=33.93;ReadPosRankSum=-9.450e-01;SOR=0.563	GT:AD:DP:GQ:PL	1/1:43,3638:3700:99:124922,9492,0	BA.5	yes

23933	A	C	45804.06	PASS	AC=2;AF=1.00;AN=2;BaseQRankSum=0.799;DP=1571;ExcessHet=0.0000;FS=0.000;MLEAC=2;MLEAF=1.00;MQ=60.00;MQRankSum=0.00;QD=31.85;ReadPosRankSum=0.854;SOR=0.413	GT:AD:DP:GQ:PL	1/1:8,1430:1438:99:45818,4082,0	BA.5	no
23948	G	T	132767.1	PASS	AC=2;AF=1.00;AN=2;BaseQRankSum=2.93;DP=3837;ExcessHet=0.0000;FS=0.000;MLEAC=2;MLEAF=1.00;MQ=60.00;MQRankSum=0.00;QD=28.76;ReadPosRankSum=-2.023e+00;SOR=0.733	GT:AD:DP:GQ:PL	1/1:2,3680:3692:99:132781,11041,0	BA.5	yes
23959	T	C	35.48	PASS	AC=2;AF=1.00;AN=2;DP=1;ExcessHet=0.0000;FS=0.000;MLEAC=1;MLEAF=0.500;MQ=60.00;QD=31.26;SOR=1.609	GT:AD:DP:GQ:PGT:PID:PL:PS	1|1:0,1:1:3:1|1:23957_G_GA:45,3,0:23957	BA.5	no
23960	T	C	35.48	PASS	AC=2;AF=1.00;AN=2;DP=1;ExcessHet=0.0000;FS=0.000;MLEAC=1;MLEAF=0.500;MQ=60.00;QD=27.76;SOR=1.609	GT:AD:DP:GQ:PGT:PID:PL:PS	1|1:0,1:1:3:1|1:23957_G_GA:45,3,0:23957	BA.5	no
23961	T	C	35.48	PASS	AC=2;AF=1.00;AN=2;DP=1;ExcessHet=0.0000;FS=0.000;MLEAC=1;MLEAF=0.500;MQ=60.00;QD=29.09;SOR=1.609	GT:AD:DP:GQ:PGT:PID:PL:PS	1|1:0,1:1:3:1|1:23957_G_GA:45,3,0:23957	BA.5	no

24210	C	T	176.02	PASS	AC=2;AF=1.00;AN=2;DP=7;ExcessHet=0.0000;FS=0.000;MLEAC=2;MLEAF=1.00;MQ=60.00;QD=25.15;SOR=0.941	GT:AD:DP:GQ:PL	1/1:0,7:7:21:190,21,0	BA.5	yes
24424	A	T	232854.1	PASS	AC=2;AF=1.00;AN=2;DP=5665;ExcessHet=0.0000;FS=0.000;MLEAC=2;MLEAF=1.00;MQ=60.00;QD=32.62;SOR=0.841	GT:AD:DP:GQ:PL	1/1:0,5508:5513:99:232868,16564,0	BA.5	yes
24442	C	T	90932.06	PASS	AC=2;AF=1.00;AN=2;BaseQRankSum=-9.650e-01;DP=2173;ExcessHet=0.0000;FS=0.000;MLEAC=2;MLEAF=1.00;MQ=60.00;MQRankSum=0.00;QD=32.91;ReadPosRankSum=1.69;SOR=0.403	GT:AD:DP:GQ:PGT:PID:PL:PS	1|1:3,2048:2051:99:1|1:24424_A_T:90946,6138,0:24424	BA.5	no
24469	T	A	230851.1	PASS	AC=2;AF=1.00;AN=2;BaseQRankSum=-5.040e-01;DP=5510;ExcessHet=0.0000;FS=0.000;MLEAC=2;MLEAF=1.00;MQ=60.00;MQRankSum=0.532;QD=29.33;ReadPosRankSum=1.01;SOR=0.149	GT:AD:DP:GQ:PL	1/1:2,5441:5446:99:230865,16321,0	BA.5	yes
24515	G	A	32.64	PASS	AC=1;AF=0.500;AN=2;BaseQRankSum=-9.670e-01;DP=3;ExcessHet=0.0000;FS=0.000;MLEAC=1;MLEAF=0.500;MQ=60.00;MQRankSum=0.00;QD=10.88;ReadPosRankSum=-9.670e-01;SOR=0.223	GT:AD:DP:GQ:PL	0/1:1,2:3:33:40,0,33	BA.5	no
24589	T	C	37.32	PASS	AC=2;AF=1.00;AN=2;DP=2;ExcessHet=0.0000;FS=0.000;MLEAC=1;MLEAF=0.500;MQ=60.00;QD=18.66;SOR=0.693	GT:AD:DP:GQ:PL	1/1:0,2:2:6:49,6,0	BA.5	no

24998	G	T	255.97	PASS	AC=2;AF=1.00;AN=2;DP=6;ExcessHet=0.0000;FS=0.000;MLEAC=2;MLEAF=1.00;MQ=60.00;QD=26.61;SOR=0.693	GT:AD:DP:GQ:PGT:PID:PL:PS	1|1:0,6:6:18:1|1:24998_G_T:270,18,0:24998	BA.5	no
25000	C	T	199600.1	PASS	AC=2;AF=1.00;AN=2;BaseQRankSum=2.27;DP=5372;ExcessHet=0.0000;FS=0.000;MLEAC=2;MLEAF=1.00;MQ=60.00;MQRankSum=-3.850e-01;QD=35.36;ReadPosRankSum=2.14;SOR=0.566	GT:AD:DP:GQ:PL	1/1:3,5221:5224:99:199614,15612,0	BA.5	yes
25169	C	T	85.14	PASS	AC=2;AF=1.00;AN=2;DP=6;ExcessHet=0.0000;FS=0.000;MLEAC=2;MLEAF=1.00;MQ=60.00;QD=21.29;SOR=0.693	GT:AD:DP:GQ:PL	1/1:0,4:4:12:99,12,0	BA.5	no
25290	G	T	5239.64	PASS	AC=1;AF=0.500;AN=2;BaseQRankSum=2.11;DP=582;ExcessHet=0.0000;FS=1.101;MLEAC=1;MLEAF=0.500;MQ=60.00;MQRankSum=0.00;QD=9.70;ReadPosRankSum=4.14;SOR=0.753	GT:AD:DP:GQ:PL	0/1:296,244:540:99:5247,0,6368	BA.5	yes
25350	C	T	344499.06	PASS	AC=2;AF=1.00;AN=2;BaseQRankSum=-1.949e+00;DP=1286;ExcessHet=0.0000;FS=0.000;MLEAC=2;MLEAF=1.00;MQ=60.00;MQRankSum=0.00;QD=28.42;ReadPosRankSum=0.074;SOR=0.236	GT:AD:DP:GQ:PL	1/1:1,1213:1214:99:34513,3596,0	BA.5	no
25584	C	T	214267.1	PASS	AC=2;AF=1.00;AN=2;BaseQRankSum=1.31;DP=5821;ExcessHet=0.0000;FS=0.000;MLEAC=2;MLEAF=1.00;MQ=60.00;MQRankSum=0.00;QD=34.30;ReadPosRankSum=0.542;SOR=0.652	GT:AD:DP:GQ:PL	1/1:3,5623:5627:99:214281,16846,0	BA.5	yes

26022	C	T	37.32	PASS	AC=2;AF=1.00;AN=2;DP=2;ExcessHet=0.0000;FS=0.000;MLEAC=1;MLEAF=0.500;MQ=60.00;QD=18.66;SOR=0.693	GT:AD:DP:GQ:PL	1/1:0,2:2:6:49,6,0	BA.5	yes
26042	C	T	52.64	PASS	AC=1;AF=0.500;AN=2;BaseQRankSum=-2.530e-01;DP=10;ExcessHet=0.0000;FS=3.310;MLEAC=1;MLEAF=0.500;MQ=60.00;MQRankSum=0.00;QD=5.26;ReadPosRankSum=0.00;SOR=2.303	GT:AD:DP:GQ:PGT:PID:PL:PS	0|1:8,2:10:60:0|1:26042_C_T:60,0,330:26042	BA.5	no
26044	C	G	52.64	PASS	AC=1;AF=0.500;AN=2;BaseQRankSum=-1.348e+00;DP=10;ExcessHet=0.0000;FS=3.310;MLEAC=1;MLEAF=0.500;MQ=60.00;MQRankSum=0.00;QD=5.26;ReadPosRankSum=0.00;SOR=2.303	GT:AD:DP:GQ:PGT:PID:PL:PS	0|1:8,2:10:60:0|1:26042_C_T:60,0,330:26042	BA.5	no
26046	A	G	52.64	PASS	AC=1;AF=0.500;AN=2;BaseQRankSum=-3.190e-01;DP=10;ExcessHet=0.0000;FS=3.332;MLEAC=1;MLEAF=0.500;MQ=60.00;MQRankSum=0.00;QD=6.58;ReadPosRankSum=-6.190e-01;SOR=2.303	GT:AD:DP:GQ:PL	0/1:6,2:8:60:60,0,330	BA.5	no
26060	C	T	226765.1	PASS	AC=2;AF=1.00;AN=2;BaseQRankSum=9.29;DP=5981;ExcessHet=0.0000;FS=0.000;MLEAC=2;MLEAF=1.00;MQ=60.00;MQRankSum=0.00;QD=27.00;ReadPosRankSum=4.62;SOR=0.214	GT:AD:DP:GQ:PL	1/1:17,5421:5445:99:226779,16863,0	BA.5	yes
26192	C	T	176.02	PASS	AC=2;AF=1.00;AN=2;DP=7;ExcessHet=0.0000;FS=0.000;MLEAC=2;MLEAF=1.00;MQ=60.00;QD=25.15;SOR=0.941	GT:AD:DP:	1/1:0,7:7:21:190,21,0	BA.5	no

						GQ:PL			
26270	C	T	228138.1	PASS	AC=2;AF=1.00;AN=2;BaseQRankSum=3.79;DP=5939;ExcessHet=0.0000;FS=0.000;MLEAC=2;MLEAF=1.00;MQ=60.00;MQRankSum=0.00;QD=30.82;ReadPosRankSum=2.41;SOR=1.208	GT:AD:DP:GQ:PL	1/1:2,5749:5753:99:228152,17240,0	BA.5	yes
26505	G	A	32.48	PASS	AC=2;AF=1.00;AN=2;DP=1;ExcessHet=0.0000;FS=0.000;MLEAC=1;MLEAF=0.500;MQ=60.00;QD=32.48;SOR=1.609	GT:AD:DP:GQ:PL	1/1:0,1:1:3:42,3,0	BA.5	no
26529	G	A	236700.1	PASS	AC=2;AF=1.00;AN=2;BaseQRankSum=0.081;DP=5608;ExcessHet=0.0000;FS=0.000;MLEAC=2;MLEAF=1.00;MQ=60.00;MQRankSum=0.00;QD=29.27;ReadPosRankSum=0.997;SOR=0.594	GT:AD:DP:GQ:PL	1/1:2,5523:5525:99:236714,16564,0	BA.5	yes
26577	C	G	248009.1	PASS	AC=2;AF=1.00;AN=2;DP=5865;ExcessHet=0.0000;FS=0.000;MLEAC=2;MLEAF=1.00;MQ=60.00;MQRankSum=0.00;QD=35.92;SOR=0.741	GT:AD:DP:GQ:PL	1/1:4,5791:5798:99:248023,17303,0	BA.5	yes
26709	G	A	230694.1	PASS	AC=2;AF=1.00;AN=2;BaseQRankSum=3.15;DP=5971;ExcessHet=0.0000;FS=0.000;MLEAC=2;MLEAF=1.00;MQ=60.00;MQRankSum=-2.000e-03;QD=29.63;ReadPosRankSum=3.46;SOR=0.461	GT:AD:DP:GQ:PL	1/1:5,5792:5797:99:230708,17313,0	BA.5	yes
27012	C	T	216080.1	PASS	AC=2;AF=1.00;AN=2;BaseQRankSum=0.160;DP=5784;ExcessHet=0.0000;FS=0.000;MLEAC=2;MLEAF=1.00;MQ=60.00;MQRankSum=-3.510e-01;QD=28.76;ReadPosRankSum=2.69;SOR=0.266	GT:AD:DP:GQ:PL	1/1:5,5535:5540:99:216094,16523,0	BA.5	yes

27038	A	G	141993.64	PASS	AC=1;AF=0.500;AN=2;BaseQRankSum=-3.900e-01;DP=1033;ExcessHet=0.0000;FS=0.526;MLEAC=1;MLEAF=0.500;MQ=60.00;MQRankSum=0.00;QD=14.72;ReadPosRankSum=3.22;SOR=0.733	GT:AD:DP:GQ:PL	0/1:397,567:964:99:14201,0,9112	BA.5	yes
27335	C	T	392.64	PASS	AC=1;AF=0.500;AN=2;BaseQRankSum=-1.163e+00;DP=175;ExcessHet=0.0000;FS=0.767;MLEAC=1;MLEAF=0.500;MQ=60.00;MQRankSum=0.00;QD=2.28;ReadPosRankSum=-4.711e+00;SOR=0.849	GT:AD:DP:GQ:PGT:PID:PL:PS	0|1:143,29:172:99:0|1:27335_C_T:400,0,5535:27335	BA.5	no
27438	T	C	48338.06	PASS	AC=2;AF=1.00;AN=2;DP=1781;ExcessHet=0.0000;FS=0.000;MLEAC=2;MLEAF=1.00;MQ=60.00;QD=28.20;SOR=0.722	GT:AD:DP:GQ:PL	1/1:0,1714:1714:99:48352,5130,0	BA.5	yes
27476	C	T	97117.06	PASS	AC=2;AF=1.00;AN=2;BaseQRankSum=-4.188e+00;DP=2545;ExcessHet=0.0000;FS=0.000;MLEAC=2;MLEAF=1.00;MQ=60.00;MQRankSum=0.00;QD=26.08;ReadPosRankSum=2.80;SOR=0.004	GT:AD:DP:GQ:PL	1/1:14,2173:2187:99:97131,6843,0	BA.5	yes
27513	C	T	206059.1	PASS	AC=2;AF=1.00;AN=2;BaseQRankSum=-4.290e-01;DP=5426;ExcessHet=0.0000;FS=0.000;MLEAC=2;MLEAF=1.00;MQ=60.00;MQRankSum=-4.820e-01;QD=29.94;ReadPosRankSum=1.61;SOR=0.726	GT:AD:DP:GQ:PL	1/1:2,5258:5260:99:206073,15732,0	BA.5	yes
27532	C	T	6574.64	PASS	AC=1;AF=0.500;AN=2;BaseQRankSum=1.20;DP=460;ExcessHet=0.0000;FS=1.148;MLEAC=1;MLEAF=0.500;MQ=60.00;MQRankSum=	GT:AD:DP:GQ:PL	0/1:163,274:437:99:6582,0,3345	BA.5	yes

<table>
<tr><td></td><td></td><td></td><td></td><td></td><td>0.00;QD=15.04;ReadPosRankSum=0.836;SOR=0.589</td><td></td><td></td><td></td><td></td></tr>
<tr><td>27807</td><td>C</td><td>T</td><td>225790.1</td><td>PASS</td><td>AC=2;AF=1.00;AN=2;BaseQRankSum=-3.750e-01;DP=5934;ExcessHet=0.0000;FS=0.000;MLEAC=2;MLEAF=1.00;MQ=60.00;MQRankSum=0.00;QD=31.54;ReadPosRankSum=0.330;SOR=0.242</td><td>GT:AD:DP:GQ:PL</td><td>1/1:1,4299:4326:99:225804,17113,0</td><td>BA.5</td><td>yes</td></tr>
<tr><td>27889</td><td>C</td><td>T</td><td>229649.1</td><td>PASS</td><td>AC=2;AF=1.00;AN=2;BaseQRankSum=4.02;DP=5954;ExcessHet=0.0000;FS=0.000;MLEAC=2;MLEAF=1.00;MQ=60.00;MQRankSum=0.351;QD=28.86;ReadPosRankSum=2.66;SOR=1.138</td><td>GT:AD:DP:GQ:PL</td><td>1/1:6,5776:5782:99:229663,17208,0</td><td>BA.5</td><td>yes</td></tr>
<tr><td>27998</td><td>C</td><td>T</td><td>52.64</td><td>PASS</td><td>AC=1;AF=0.500;AN=2;BaseQRankSum=-3.960e-01;DP=13;ExcessHet=0.0000;FS=0.000;MLEAC=1;MLEAF=0.500;MQ=60.00;MQRankSum=0.00;QD=4.05;ReadPosRankSum=0.362;SOR=0.527</td><td>GT:AD:DP:GQ:PL</td><td>0/1:9,4:13:60:60,0,201</td><td>BA.5</td><td>no</td></tr>
<tr><td>28088</td><td>T</td><td>C</td><td>16997.64</td><td>PASS</td><td>AC=1;AF=0.500;AN=2;BaseQRankSum=-8.210e-01;DP=2312;ExcessHet=0.0000;FS=0.546;MLEAC=1;MLEAF=0.500;MQ=60.00;MQRankSum=0.00;QD=7.74;ReadPosRankSum=0.413;SOR=0.768</td><td>GT:AD:DP:GQ:PL</td><td>0/1:1439,757:2196:99:17005,0,38930</td><td>BA.5</td><td>no</td></tr>
<tr><td>28251</td><td>T</td><td>G</td><td>70.64</td><td>PASS</td><td>AC=1;AF=0.500;AN=2;BaseQRankSum=-1.760e-01;DP=19;ExcessHet=0.0000;FS=0.000;MLEAC=1;MLEAF=0.500;MQ=60.00;MQRankSum=0.00;QD=3.72;ReadPosRankSum=-2.081e+00;SOR=1.179</td><td>GT:AD:DP:GQ:PGT:PID:PL:PS</td><td>0|1:16,3:19:78:0|1:28251_T_G:78,0,662:28251</td><td>BA.5</td><td>no</td></tr>
<tr><td>28252</td><td>T</td><td>A</td><td>70.64</td><td>PA</td><td>AC=1;AF=0.500;AN=2;BaseQRankSum=-1.760e-01;DP=20;ExcessHet=0.000</td><td>GT:AD:</td><td>0|1:16,3:19:78:0|1:2</td><td>BA.5</td><td>no</td></tr>
</table>

				SS	0;FS=0.000;MLEAC=1;MLEAF=0.500;MQ=60.00;MQRankSum=0.00;QD=3.72;ReadPosRankSum=-1.971e+00;SOR=1.179	DP:GQ:PGT:PID:PL:PS	8251_T_G:78,0,662:28251		
28253	C	A	70.64	PASS	AC=1;AF=0.500;AN=2;BaseQRankSum=-5.240e-01;DP=20;ExcessHet=0.0000;FS=0.000;MLEAC=1;MLEAF=0.500;MQ=60.00;MQRankSum=0.00;QD=3.72;ReadPosRankSum=-1.631e+00;SOR=1.179	GT:AD:DP:GQ:PGT:PID:PL:PS	0|1:16,3:19:78:0|1:28251_T_G:78,0,662:28251	BA.5	no
28271	A	T	238437.1	PASS	AC=2;AF=1.00;AN=2;BaseQRankSum=-4.060e-01;DP=5985;ExcessHet=0.0000;FS=0.000;MLEAC=2;MLEAF=1.00;MQ=60.00;MQRankSum=0.825;QD=29.41;ReadPosRankSum=0.046;SOR=0.416	GT:AD:DP:GQ:PGT:PID:PL:PS	1|1:1,5661:5662:99:1|1:28271_A_T:238451,17084,0:28271	BA.5	yes
28311	C	T	253000.1	PASS	AC=2;AF=1.00;AN=2;BaseQRankSum=-4.950e-01;DP=5656;ExcessHet=0.0000;FS=0.000;MLEAC=2;MLEAF=1.00;MQ=60.00;MQRankSum=0.00;QD=31.11;ReadPosRankSum=1.31;SOR=0.292	GT:AD:DP:GQ:PGT:PID:PL:PS	1|1:1,5654:5655:99:1|1:28271_A_T:253014,16975,0:28271	BA.5	yes
28330	A	G	240337.1	PASS	AC=2;AF=1.00;AN=2;DP=5383;ExcessHet=0.0000;FS=0.000;MLEAC=2;MLEAF=1.00;MQ=60.00;QD=34.66;SOR=0.807	GT:AD:DP:GQ:PL	1/1:0,5164:5167:99:240351,16167,0	BA.5	yes
28344	C	T	35.48	PA	AC=2;AF=1.00;AN=2;DP=1;ExcessHet=0.0000;FS=0.000;MLEAC=1;MLEAF=0.5	GT:AD:	1|1:0,1:1:3:1|1:2834	BA.5	no

				SS	00;MQ=60.00;QD=25.41;SOR=1.609	DP:GQ:PGT:PID:PL:PS	1_C_CA:45,3,0:28341		
28349	A	C	35.48	PASS	AC=2;AF=1.00;AN=2;DP=1;ExcessHet=0.0000;FS=0.000;MLEAC=1;MLEAF=0.500;MQ=60.00;QD=28.50;SOR=1.609	GT:AD:DP:GQ:PGT:PID:PL:PS	1|1:0,1:1:3:1|1:28341_C_CA:45,3,0:28341	BA.5	no
28509	G	T	74478.06	PASS	AC=2;AF=1.00;AN=2;BaseQRankSum=3.97;DP=2609;ExcessHet=0.0000;FS=0.000;MLEAC=2;MLEAF=1.00;MQ=60.00;MQRankSum=0.00;QD=30.30;ReadPosRankSum=1.28;SOR=0.029	GT:AD:DP:GQ:PL	1/1:5,2453:2458:99:74492,7296,0	BA.5	yes
28881	G	A	207932.1	PASS	AC=2;AF=1.00;AN=2;BaseQRankSum=-1.608e+00;DP=4835;ExcessHet=0.0000;FS=0.000;MLEAC=2;MLEAF=1.00;MQ=60.00;MQRankSum=0.090;QD=36.76;ReadPosRankSum=4.16;SOR=0.079	GT:AD:DP:GQ:PL	1/1:17,4650:4671:99:207946,12777,0	BA.5	yes
28882	G	A	180709.1	PASS	AC=2;AF=1.00;AN=2;BaseQRankSum=-1.000e-01;DP=4815;ExcessHet=0.0000;FS=0.000;MLEAC=2;MLEAF=1.00;MQ=60.00;MQRankSum=0.00;QD=30.54;ReadPosRankSum=1.16;SOR=0.165	GT:AD:DP:GQ:PL	1/1:25,4608:4635:99:208315,12806,0	BA.5	yes
28883	G	C	208133.1	PASS	AC=2;AF=1.00;AN=2;BaseQRankSum=1.57;DP=4736;ExcessHet=0.0000;FS=0.000;MLEAC=2;MLEAF=1.00;MQ=60.00;MQRankSum=-9.300e-	GT:AD:DP:GQ:PL	1/1:16,4612:4645:99:208208,13021,0	BA.5	yes

<table>
<tr><td></td><td></td><td></td><td></td><td></td><td>02;QD=21.53;ReadPosRankSum=4.93;SOR=0.079</td><td></td><td></td><td></td><td></td></tr>
<tr><td>28994</td><td>C</td><td>A</td><td>159309.1</td><td>PASS</td><td>AC=2;AF=1.00;AN=2;BaseQRankSum=-1.003e+00;DP=4294;ExcessHet=0.0000;FS=0.000;MLEAC=2;MLEAF=1.00;MQ=60.00;MQRankSum=0.00;QD=29.53;ReadPosRankSum=2.87;SOR=0.465</td><td>GT:AD:DP:GQ:PL</td><td>1/1:12,4164:4176:99:159323,12050,0</td><td>BA.5</td><td>yes</td></tr>
<tr><td>29029</td><td>T</td><td>C</td><td>31.64</td><td>PASS</td><td>AC=1;AF=0.500;AN=2;BaseQRankSum=-6.740e-01;DP=2;ExcessHet=0.0000;FS=0.000;MLEAC=1;MLEAF=0.500;MQ=60.00;MQRankSum=0.00;QD=15.82;ReadPosRankSum=0.674;SOR=1.447</td><td>GT:AD:DP:GQ:PGT:PID:PL:PS</td><td>0|1:1,1:2:39:0|1:29029_T_C:39,0,39:29029</td><td>BA.5</td><td>no</td></tr>
<tr><td>29039</td><td>A</td><td>T</td><td>31.64</td><td>PASS</td><td>AC=1;AF=0.500;AN=2;BaseQRankSum=-6.740e-01;DP=2;ExcessHet=0.0000;FS=0.000;MLEAC=1;MLEAF=0.500;MQ=60.00;MQRankSum=0.00;QD=15.82;ReadPosRankSum=0.674;SOR=1.447</td><td>GT:AD:DP:GQ:PGT:PID:PL:PS</td><td>0|1:1,1:2:39:0|1:29029_T_C:39,0,39:29029</td><td>BA.5</td><td>no</td></tr>
<tr><td>29049</td><td>G</td><td>A</td><td>35.48</td><td>PASS</td><td>AC=2;AF=1.00;AN=2;DP=1;ExcessHet=0.0000;FS=0.000;MLEAC=1;MLEAF=0.500;MQ=60.00;QD=31.31;SOR=1.609</td><td>GT:AD:DP:GQ:PGT:PID:PL:PS</td><td>1|1:0,1:1:3:1|1:29029_T_C:45,3,0:29029</td><td>BA.5</td><td>no</td></tr>
<tr><td>29090</td><td>G</td><td>T</td><td>67655.06</td><td>PASS</td><td>AC=2;AF=1.00;AN=2;DP=2228;ExcessHet=0.0000;FS=0.000;MLEAC=2;MLEAF=1.00;MQ=60.00;QD=31.35;SOR=0.903</td><td>GT:AD:DP:GQ:PL</td><td>1/1:0,2158:2158:99:67669,6475,0</td><td>BA.5</td><td>yes</td></tr>
</table>

29187	C	T	37.64	PASS	AC=1;AF=0.500;AN=2;BaseQRankSum=-9.340e-01;DP=16;ExcessHet=0.0000;FS=0.000;MLEAC=1;MLEAF=0.500;MQ=60.00;MQRankSum=0.00;QD=2.51;ReadPosRankSum=-1.791e+00;SOR=0.569	GT:AD:DP:GQ:PGT:PID:PL:PS	0|1:13,2:15:45:0|1:29187_C_T:45,0,540:29187	BA.5	no
29188	A	G	37.64	PASS	AC=1;AF=0.500;AN=2;BaseQRankSum=-9.340e-01;DP=15;ExcessHet=0.0000;FS=0.000;MLEAC=1;MLEAF=0.500;MQ=60.00;MQRankSum=0.00;QD=2.51;ReadPosRankSum=-1.967e+00;SOR=0.569	GT:AD:DP:GQ:PGT:PID:PL:PS	0|1:13,2:15:45:0|1:29187_C_T:45,0,540:29187	BA.5	no
29510	A	C	207658.1	PASS	AC=2;AF=1.00;AN=2;BaseQRankSum=3.92;DP=5606;ExcessHet=0.0000;FS=0.000;MLEAC=2;MLEAF=1.00;MQ=60.00;MQRankSum=0.347;QD=34.87;ReadPosRankSum=3.36;SOR=0.420	GT:AD:DP:GQ:PL	1/1:4,5290:5294:99:207672,15817,0	BA.5	yes
29614	C	T	94152.06	PASS	AC=2;AF=1.00;AN=2;DP=2735;ExcessHet=0.0000;FS=0.000;MLEAC=2;MLEAF=1.00;MQ=60.00;QD=26.08;SOR=1.010	GT:AD:DP:GQ:PL	1/1:0,2588:2589:99:94166,8038,0	BA.5	yes
29632	C	T	35953.06	PASS	AC=2;AF=1.00;AN=2;BaseQRankSum=-6.040e-01;DP=1437;ExcessHet=0.0000;FS=0.000;MLEAC=2;MLEAF=1.00;MQ=60.00;MQRankSum=0.00;QD=26.57;ReadPosRankSum=1.32;SOR=0.849	GT:AD:DP:GQ:PL	1/1:29,1324:1356:99:35967,2754,0	BA.5	yes
29666	C	T	54998.06	PASS	AC=2;AF=1.00;AN=2;DP=1539;ExcessHet=0.0000;FS=0.000;MLEAC=2;MLEAF=1.00;MQ=60.00;QD=33.85;SOR=0.978	GT:AD:DP:	1/1:0,1513:1513:99:55012	BA.5	yes

<table>
<tr><th></th><th></th><th></th><th></th><th></th><th></th><th></th><th></th><th></th><th></th></tr>
<tr><td></td><td></td><td></td><td></td><td></td><td></td><td>GQ:PL</td><td>,4539,0</td><td></td><td></td></tr>
<tr><td>29810</td><td>G</td><td>T</td><td>85.14</td><td>PASS</td><td>AC=2;AF=1.00;AN=2;DP=4;ExcessHet=0.0000;FS=0.000;MLEAC=2;MLEAF=1.00;MQ=60.00;QD=21.29;SOR=0.693</td><td>GT:AD:DP:GQ:PL</td><td>1/1:0,4:4:12:99,12,0</td><td>BA.5</td><td>yes</td></tr>
<tr><td>29868</td><td>G</td><td>A</td><td>876.06</td><td>PASS</td><td>AC=2;AF=1.00;AN=2;DP=35;ExcessHet=0.0000;FS=0.000;MLEAC=2;MLEAF=1.00;MQ=60.00;QD=30.21;SOR=1.460</td><td>GT:AD:DP:GQ:PL</td><td>1/1:0,29:29:87:890,87,0</td><td>BA.5</td><td>yes</td></tr>
<tr><td>29871</td><td>A</td><td>T</td><td>118.84</td><td>PASS</td><td>AC=2;AF=1.00;AN=2;DP=3;ExcessHet=0.0000;FS=0.000;MLEAC=1;MLEAF=0.500;MQ=60.00;QD=35.36;SOR=1.179</td><td>GT:AD:DP:GQ:PGT:PID:PL:PS</td><td>1|1:0,3:3:9:1|1:29868_G_A:132,9,0:29868</td><td>BA.5</td><td>no</td></tr>
<tr><td>44</td><td>C</td><td>T</td><td>27593.06</td><td>PASS</td><td>AC=2;AF=1.00;AN=2;DP=850;ExcessHet=0.0000;FS=0.000;MLEAC=2;MLEAF=1.00;MQ=60.00;QD=34.45;SOR=1.076</td><td>GT:AD:DP:GQ:PL</td><td>1/1:0,801:801:99:27607,2403,0</td><td>BF.7</td><td>yes</td></tr>
<tr><td>210</td><td>G</td><td>T</td><td>225744.1</td><td>PASS</td><td>AC=2;AF=1.00;AN=2;DP=5307;ExcessHet=0.0000;FS=0.000;MLEAC=2;MLEAF=1.00;MQ=60.00;QD=25.36;SOR=0.848</td><td>GT:AD:DP:GQ:PGT:PID:PL:PS</td><td>1|1:0,5246:5246:99:1|1:210_G_T:225758,15783,0:210</td><td>BF.7</td><td>yes</td></tr>
<tr><td>213</td><td>G</td><td>T</td><td>4797.06</td><td>PASS</td><td>AC=2;AF=1.00;AN=2;DP=175;ExcessHet=0.0000;FS=0.000;MLEAC=2;MLEAF=1.00;MQ=60.00;QD=27.89;SOR=0.815</td><td>GT:AD:DP:GQ:PL</td><td>1/1:0,172:172:99:4811,516,0</td><td>BF.7</td><td>yes</td></tr>
</table>

241	C	T	242253.1	PASS	AC=2;AF=1.00;AN=2;DP=5858;ExcessHet=0.0000;FS=0.000;MLEAC=2;MLEAF=1.00;MQ=60.00;QD=28.73;SOR=0.974	GT:AD:DP:GQ:PL	1/1:0,5793:5793:99:242267,17421,0	BF.7	yes
670	T	G	234716.1	PASS	AC=2;AF=1.00;AN=2;DP=6101;ExcessHet=0.0000;FS=0.000;MLEAC=2;MLEAF=1.00;MQ=60.00;QD=30.97;SOR=0.725	GT:AD:DP:GQ:PL	1/1:0,5920:5948:99:234730,17799,0	BF.7	yes
1085	G	T	215236.1	PASS	AC=2;AF=1.00;AN=2;DP=5615;ExcessHet=0.0000;FS=0.000;MLEAC=2;MLEAF=1.00;MQ=60.00;QD=27.24;SOR=0.793	GT:AD:DP:GQ:PL	1/1:0,5436:5438:99:215250,16612,0	BF.7	yes
1427	C	T	1309.06	PASS	AC=2;AF=1.00;AN=2;DP=55;ExcessHet=0.0000;FS=0.000;MLEAC=2;MLEAF=1.00;MQ=60.00;QD=26.72;SOR=0.733	GT:AD:DP:GQ:PL	1/1:0,49:49:99:1323,147,0	BF.7	no
1444	G	A	78.32	PASS	AC=2;AF=1.00;AN=2;DP=2;ExcessHet=0.0000;FS=0.000;MLEAC=1;MLEAF=0.500;MQ=60.00;QD=28.17;SOR=0.693	GT:AD:DP:GQ:PGT:PID:PL:PS	1|1:0,2:2:6:1|1:1434_A_AGT:90,6,0:1434	BF.7	no
1474	T	A	34.64	PASS	AC=1;AF=0.500;AN=2;BaseQRankSum=0.00;DP=16;ExcessHet=0.0000;FS=0.000;MLEAC=1;MLEAF=0.500;MQ=60.00;MQRankSum=0.00;QD=2.16;ReadPosRankSum=0.828;SOR=0.693	GT:AD:DP:GQ:PGT:PID:PL:PS	0|1:14,2:16:42:0|1:1472_A_AG:42,0,582:1472	BF.7	no
1479	C	G	34.64	PA	AC=1;AF=0.500;AN=2;BaseQRankSum=0.00;DP=16;E	GT:A	0|1:14,2:16:4	BF.7	no

<table>
<tr><td></td><td></td><td></td><td></td><td>SS</td><td>xcessHet=0.0000;FS=0.000;MLEAC=1;MLEAF=0.500;MQ=60.00;MQRankSum=0.00;QD=2.16;ReadPosRankSum=-1.526e+00;SOR=0.693</td><td>D:DP:GQ:PGT:PID:PL:PS</td><td>2:0|1:1472_A_AG:42,0,582:1472</td><td></td><td></td></tr>
<tr><td>1480</td><td>C</td><td>T</td><td>34.64</td><td>PASS</td><td>AC=1;AF=0.500;AN=2;BaseQRankSum=0.00;DP=16;ExcessHet=0.0000;FS=0.000;MLEAC=1;MLEAF=0.500;MQ=60.00;MQRankSum=0.00;QD=2.16;ReadPosRankSum=-2.135e+00;SOR=0.693</td><td>GT:AD:DP:GQ:PGT:PID:PL:PS</td><td>0|1:14,2:16:42:0|1:1472_A_AG:42,0,582:1472</td><td>BF.7</td><td>no</td></tr>
<tr><td>1627</td><td>C</td><td>T</td><td>216143.1</td><td>PASS</td><td>AC=2;AF=1.00;AN=2;BaseQRankSum=0.148;DP=5902;ExcessHet=0.0000;FS=0.000;MLEAC=2;MLEAF=1.00;MQ=60.00;MQRankSum=0.00;QD=28.20;ReadPosRankSum=2.69;SOR=0.293</td><td>GT:AD:DP:GQ:PL</td><td>1/1:4,4385:4393:99:216157,16738,0</td><td>BF.7</td><td>yes</td></tr>
<tr><td>1727</td><td>G</td><td>A</td><td>35.48</td><td>PASS</td><td>AC=2;AF=1.00;AN=2;DP=1;ExcessHet=0.0000;FS=0.000;MLEAC=1;MLEAF=0.500;MQ=60.00;QD=26.80;SOR=1.609</td><td>GT:AD:DP:GQ:PGT:PID:PL:PS</td><td>1|1:0,1:1:3:1|1:1727_G_A:45,3,0:1727</td><td>BF.7</td><td>no</td></tr>
<tr><td>1733</td><td>A</td><td>G</td><td>35.48</td><td>PASS</td><td>AC=2;AF=1.00;AN=2;DP=1;ExcessHet=0.0000;FS=0.000;MLEAC=1;MLEAF=0.500;MQ=60.00;QD=30.02;SOR=1.609</td><td>GT:AD:DP:GQ:PGT:PID:PL:PS</td><td>1|1:0,1:1:3:1|1:1727_G_A:45,3,0:1727</td><td>BF.7</td><td>no</td></tr>
</table>

1734	C	A	35.48	PASS	AC=2;AF=1.00;AN=2;DP=1;ExcessHet=0.0000;FS=0.000;MLEAC=1;MLEAF=0.500;MQ=60.00;QD=31.98;SOR=1.609	GT:AD:DP:GQ:PGT:PID:PL:PS	1|1:0,1:1:3:1|1:1727_G_A:45,3,0:1727	BF.7	no
1736	G	A	35.48	PASS	AC=2;AF=1.00;AN=2;DP=1;ExcessHet=0.0000;FS=0.000;MLEAC=1;MLEAF=0.500;MQ=60.00;QD=27.51;SOR=1.609	GT:AD:DP:GQ:PGT:PID:PL:PS	1|1:0,1:1:3:1|1:1727_G_A:45,3,0:1727	BF.7	no
1738	G	A	35.48	PASS	AC=2;AF=1.00;AN=2;DP=1;ExcessHet=0.0000;FS=0.000;MLEAC=1;MLEAF=0.500;MQ=60.00;QD=29.11;SOR=1.609	GT:AD:DP:GQ:PGT:PID:PL:PS	1|1:0,1:1:3:1|1:1727_G_A:45,3,0:1727	BF.7	no
1796	G	C	35.48	PASS	AC=2;AF=1.00;AN=2;DP=1;ExcessHet=0.0000;FS=0.000;MLEAC=1;MLEAF=0.500;MQ=60.00;QD=23.23;SOR=1.609	GT:AD:DP:GQ:PGT:PID:PL:PS	1|1:0,1:1:3:1|1:1769_ATTGTTGAATCCTGTGGTAATT_A:45,3,0:1769	BF.7	no
1798	T	A	35.48	PASS	AC=2;AF=1.00;AN=2;DP=1;ExcessHet=0.0000;FS=0.000;MLEAC=1;MLEAF=0.5	GT:AD:DP:	1|1:0,1:1:3:1|1:1769_ATT	BF.7	no

<table>
<tr><td></td><td></td><td></td><td></td><td></td><td>00;MQ=60.00;QD=29.03;SOR=1.609</td><td>GQ:PGT:PID:PL:PS</td><td>GTTGAATCCTGTGGTAATT_A:45,3,0:1769</td><td></td><td></td></tr>
<tr><td>1824</td><td>C</td><td>T</td><td>78.32</td><td>PASS</td><td>AC=2;AF=1.00;AN=2;DP=2;ExcessHet=0.0000;FS=0.000;MLEAC=1;MLEAF=0.500;MQ=60.00;QD=26.61;SOR=0.693</td><td>GT:AD:DP:GQ:PGT:PID:PL:PS</td><td>1|1:0,2:2:6:1|1:1824_C_T:90,6,0:1824</td><td>BF.7</td><td>no</td></tr>
<tr><td>1827</td><td>G</td><td>T</td><td>78.32</td><td>PASS</td><td>AC=2;AF=1.00;AN=2;DP=2;ExcessHet=0.0000;FS=0.000;MLEAC=1;MLEAF=0.500;MQ=60.00;QD=29.20;SOR=0.693</td><td>GT:AD:DP:GQ:PGT:PID:PL:PS</td><td>1|1:0,2:2:6:1|1:1824_C_T:90,6,0:1824</td><td>BF.7</td><td>no</td></tr>
<tr><td>1830</td><td>A</td><td>G</td><td>78.32</td><td>PASS</td><td>AC=2;AF=1.00;AN=2;DP=2;ExcessHet=0.0000;FS=0.000;MLEAC=1;MLEAF=0.500;MQ=60.00;QD=28.55;SOR=0.693</td><td>GT:AD:DP:GQ:PGT:PID:PL:PS</td><td>1|1:0,2:2:6:1|1:1824_C_T:90,6,0:1824</td><td>BF.7</td><td>no</td></tr>
<tr><td>1834</td><td>T</td><td>C</td><td>78.32</td><td>PASS</td><td>AC=2;AF=1.00;AN=2;DP=2;ExcessHet=0.0000;FS=0.000;MLEAC=1;MLEAF=0.500;MQ=60.00;QD=34.42;SOR=0.693</td><td>GT:AD:DP:GQ:PGT:PID:</td><td>1|1:0,2:2:6:1|1:1824_C_T:90,6,0:1824</td><td>BF.7</td><td>no</td></tr>
</table>

						PL:PS			
1845	A	G	73.64	PASS	AC=1;AF=0.500;AN=2;BaseQRankSum=-4.310e-01;DP=3;ExcessHet=0.0000;FS=0.000;MLEAC=1;MLEAF=0.500;MQ=60.00;MQRankSum=0.00;QD=24.55;ReadPosRankSum=-9.670e-01;SOR=0.223	GT:AD:DP:GQ:PGT:PID:PL:PS	0|1:1,2:3:36:0|1:1824_C_T:81,0,36:1824	BF.7	no
1848	C	A	35.48	PASS	AC=2;AF=1.00;AN=2;DP=3;ExcessHet=0.0000;FS=0.000;MLEAC=1;MLEAF=0.500;MQ=60.00;QD=28.53;SOR=1.609	GT:AD:DP:GQ:PGT:PID:PL:PS	1|1:0,1:1:3:1|1:1848_C_A:45,3,0:1848	BF.7	no
1851	T	G	35.48	PASS	AC=2;AF=1.00;AN=2;DP=1;ExcessHet=0.0000;FS=0.000;MLEAC=1;MLEAF=0.500;MQ=60.00;QD=27.23;SOR=1.609	GT:AD:DP:GQ:PGT:PID:PL:PS	1|1:0,1:1:3:1|1:1848_C_A:45,3,0:1848	BF.7	no
1973	C	T	220912.1	PASS	AC=2;AF=1.00;AN=2;BaseQRankSum=-8.510e-01;DP=6012;ExcessHet=0.0000;FS=0.000;MLEAC=2;MLEAF=1.00;MQ=60.00;MQRankSum=-8.500e-02;QD=25.00;ReadPosRankSum=4.21;SOR=0.722	GT:AD:DP:GQ:PL	1/1:102,5738:5840:99:220926,13503,0	BF.7	yes
2179	T	A	35.48	PASS	AC=2;AF=1.00;AN=2;DP=1;ExcessHet=0.0000;FS=0.000;MLEAC=1;MLEAF=0.500;MQ=60.00;QD=32.91;SOR=1.609	GT:AD:DP:GQ:PG	1|1:0,1:1:3:1|1:2179_T_A:45,3,0:2179	BF.7	no

						T:PID:PL:PS			
2180	G	A	35.48	PASS	AC=2;AF=1.00;AN=2;DP=1;ExcessHet=0.0000;FS=0.000;MLEAC=1;MLEAF=0.500;MQ=60.00;QD=31.31;SOR=1.609	GT:AD:DP:GQ:PGT:PID:PL:PS	1|1:0,1:1:3:1|1:2179_T_A:45,3,0:2179	BF.7	no
2359	T	C	41.64	PASS	AC=1;AF=0.500;AN=2;BaseQRankSum=2.83;DP=15;ExcessHet=0.0000;FS=6.990;MLEAC=1;MLEAF=0.500;MQ=60.00;MQRankSum=0.00;QD=2.78;ReadPosRankSum=-1.899e+00;SOR=2.807	GT:AD:DP:GQ:PGT:PID:PL:PS	0|1:12,3:15:49:0|1:2359_T_C:49,0,497:2359	BF.7	no
2438	G	C	31.64	PASS	AC=1;AF=0.500;AN=2;BaseQRankSum=0.00;DP=2;ExcessHet=0.0000;FS=0.000;MLEAC=1;MLEAF=0.500;MQ=60.00;MQRankSum=0.00;QD=15.82;ReadPosRankSum=0.674;SOR=0.693	GT:AD:DP:GQ:PGT:PID:PL:PS	0|1:1,1:2:39:0|1:2422_GTGTGTTAAATC_G:39,0,39:2422	BF.7	no
2447	G	T	31.64	PASS	AC=1;AF=0.500;AN=2;BaseQRankSum=0.00;DP=2;ExcessHet=0.0000;FS=0.000;MLEAC=1;MLEAF=0.500;MQ=60.00;MQRankSum=0.00;QD=15.82;ReadPosRankSum=0.674;SOR=0.693	GT:AD:DP:GQ:PGT:PID:PL:PS	0|1:1,1:2:39:0|1:2422_GTGTGTTAAATC_G:39,0,39:2422	BF.7	no
2451	T	C	35.48	PA	AC=2;AF=1.00;AN=2;DP=1;ExcessHet=0.0000;FS=0.0	GT:A	1|1:0,1:1:3:1|	BF.7	no

<table>
<tr><td></td><td></td><td></td><td></td><td>SS</td><td>00;MLEAC=1;MLEAF=0.500;MQ=60.00;QD=33.27;SOR=1.609</td><td>D:DP:GQ:PGT:PID:PL:PS</td><td>1:2422_GTGTGTTAAATC_G:45,3,0:2422</td><td></td><td></td></tr>
<tr><td>2493</td><td>A</td><td>C</td><td>35.48</td><td>PASS</td><td>AC=2;AF=1.00;AN=2;DP=1;ExcessHet=0.0000;FS=0.000;MLEAC=1;MLEAF=0.500;MQ=60.00;QD=29.09;SOR=1.609</td><td>GT:AD:DP:GQ:PGT:PID:PL:PS</td><td>1|1:0,1:1:3:1|1:2489_T_TACCATCA:45,3,0:2489</td><td>BF.7</td><td>no</td></tr>
<tr><td>2790</td><td>C</td><td>T</td><td>201370.1</td><td>PASS</td><td>AC=2;AF=1.00;AN=2;BaseQRankSum=-4.500e-01;DP=5388;ExcessHet=0.0000;FS=0.000;MLEAC=2;MLEAF=1.00;MQ=60.00;MQRankSum=0.622;QD=29.56;ReadPosRankSum=1.27;SOR=0.266</td><td>GT:AD:DP:GQ:PL</td><td>1/1:1,5117:5118:99:201384,15533,0</td><td>BF.7</td><td>yes</td></tr>
<tr><td>2822</td><td>C</td><td>T</td><td>35.48</td><td>PASS</td><td>AC=2;AF=1.00;AN=2;DP=1;ExcessHet=0.0000;FS=0.000;MLEAC=1;MLEAF=0.500;MQ=60.00;QD=33.85;SOR=1.609</td><td>GT:AD:DP:GQ:PGT:PID:PL:PS</td><td>1|1:0,1:1:3:1|1:2817_A_AAAAGAAAACAGATATATGTGAGC:45,3,0:2817</td><td>BF.7</td><td>no</td></tr>
<tr><td>2823</td><td>T</td><td>A</td><td>35.48</td><td>PASS</td><td>AC=2;AF=1.00;AN=2;DP=1;ExcessHet=0.0000;FS=0.000;MLEAC=1;MLEAF=0.500;MQ=60.00;QD=27.08;SOR=1.609</td><td>GT:AD:DP:GQ:PGT:P</td><td>1|1:0,1:1:3:1|1:2817_A_AAAAGAAAAC</td><td>BF.7</td><td>no</td></tr>
</table>

<table>
<tr><td></td><td></td><td></td><td></td><td></td><td></td><td>ID:PL:PS</td><td>AGATATATGTGAGC:45,3,0:2817</td><td></td><td></td></tr>
<tr><td>3037</td><td>C</td><td>T</td><td>229601.1</td><td>PASS</td><td>AC=2;AF=1.00;AN=2;DP=6036;ExcessHet=0.0000;FS=0.000;MLEAC=2;MLEAF=1.00;MQ=60.00;QD=30.62;SOR=0.759</td><td>GT:AD:DP:GQ:PL</td><td>1/1:0,5808:5811:99:229615,17334,0</td><td>BF.7</td><td>yes</td></tr>
<tr><td>3315</td><td>C</td><td>T</td><td>1766.64</td><td>PASS</td><td>AC=1;AF=0.500;AN=2;BaseQRankSum=-1.055e+00;DP=189;ExcessHet=0.0000;FS=0.000;MLEAC=1;MLEAF=0.500;MQ=60.00;MQRankSum=0.00;QD=9.60;ReadPosRankSum=0.177;SOR=0.721</td><td>GT:AD:DP:GQ:PL</td><td>0/1:102,82:184:99:1774,0,2479</td><td>BF.7</td><td>no</td></tr>
<tr><td>3559</td><td>A</td><td>G</td><td>35.48</td><td>PASS</td><td>AC=2;AF=1.00;AN=2;DP=1;ExcessHet=0.0000;FS=0.000;MLEAC=1;MLEAF=0.500;MQ=60.00;QD=29.33;SOR=1.609</td><td>GT:AD:DP:GQ:PGT:PID:PL:PS</td><td>1|1:0,1:1:3:1|1:3555_T_TGACATCCC:45,3,0:3555</td><td>BF.7</td><td>no</td></tr>
<tr><td>3571</td><td>T</td><td>A</td><td>440.06</td><td>PASS</td><td>AC=2;AF=1.00;AN=2;BaseQRankSum=0.514;DP=21;ExcessHet=0.0000;FS=3.245;MLEAC=2;MLEAF=1.00;MQ=60.00;MQRankSum=0.00;QD=23.16;ReadPosRankSum=-1.195e+00;SOR=0.556</td><td>GT:AD:DP:GQ:PL</td><td>1/1:1,18:19:32:454,32,0</td><td>BF.7</td><td>no</td></tr>
<tr><td>3614</td><td>G</td><td>C</td><td>35.48</td><td>PASS</td><td>AC=2;AF=1.00;AN=2;DP=1;ExcessHet=0.0000;FS=0.000;MLEAC=1;MLEAF=0.500;MQ=60.00;QD=35.36;SOR=1.609</td><td>GT:AD:DP:GQ:PGT:PID:</td><td>1|1:0,1:1:3:1|1:3614_G_C:45,3,0:3614</td><td>BF.7</td><td>no</td></tr>
</table>

						PL:PS			
3635	A	T	35.48	PASS	AC=2;AF=1.00;AN=2;DP=1;ExcessHet=0.0000;FS=0.000;MLEAC=1;MLEAF=0.500;MQ=60.00;QD=27.00;SOR=1.609	GT:AD:DP:GQ:PGT:PID:PL:PS	1|1:0,1:1:3:1|1:3635_A_T:45,3,0:3635	BF.7	no
3636	A	T	35.48	PASS	AC=2;AF=1.00;AN=2;DP=1;ExcessHet=0.0000;FS=0.000;MLEAC=1;MLEAF=0.500;MQ=60.00;QD=30.82;SOR=1.609	GT:AD:DP:GQ:PGT:PID:PL:PS	1|1:0,1:1:3:1|1:3635_A_T:45,3,0:3635	BF.7	no
3639	G	C	35.48	PASS	AC=2;AF=1.00;AN=2;DP=1;ExcessHet=0.0000;FS=0.000;MLEAC=1;MLEAF=0.500;MQ=60.00;QD=29.27;SOR=1.609	GT:AD:DP:GQ:PGT:PID:PL:PS	1|1:0,1:1:3:1|1:3635_A_T:45,3,0:3635	BF.7	no
3657	T	A	35.48	PASS	AC=2;AF=1.00;AN=2;DP=1;ExcessHet=0.0000;FS=0.000;MLEAC=1;MLEAF=0.500;MQ=60.00;QD=28.76;SOR=1.609	GT:AD:DP:GQ:PGT:PID:PL:PS	1|1:0,1:1:3:1|1:3635_A_T:45,3,0:3635	BF.7	no
3683	C	T	43.64	PASS	AC=1;AF=0.500;AN=2;BaseQRankSum=-8.150e-01;DP=17;ExcessHet=0.0000;FS=0.000;MLEAC=1;ML	GT:AD:DP:	0/1:12,4:16:51:51,0,283	BF.7	no

					EAF=0.500;MQ=60.00;MQRankSum=0.00;QD=2.73;ReadPosRankSum=1.06;SOR=0.446	GQ:PL			
3716	G	A	78.32	PASS	AC=2;AF=1.00;AN=2;DP=2;ExcessHet=0.0000;FS=0.000;MLEAC=1;MLEAF=0.500;MQ=60.00;QD=31.54;SOR=0.693	GT:AD:DP:GQ:PGT:PID:PL:PS	1|1:0,2:2:6:1|1:3716_G_A:90,6,0:3716	BF.7	no
3762	T	C	35.48	PASS	AC=2;AF=1.00;AN=2;DP=1;ExcessHet=0.0000;FS=0.000;MLEAC=1;MLEAF=0.500;MQ=60.00;QD=31.11;SOR=1.609	GT:AD:DP:GQ:PGT:PID:PL:PS	1|1:0,1:1:3:1|1:3758_T_TCTCGGCTGG:45,3,0:3758	BF.7	no
3765	A	T	35.48	PASS	AC=2;AF=1.00;AN=2;DP=1;ExcessHet=0.0000;FS=0.000;MLEAC=1;MLEAF=0.500;MQ=60.00;QD=34.66;SOR=1.609	GT:AD:DP:GQ:PGT:PID:PL:PS	1|1:0,1:1:3:1|1:3758_T_TCTCGGCTGG:45,3,0:3758	BF.7	no
3766	T	G	35.48	PASS	AC=2;AF=1.00;AN=2;DP=1;ExcessHet=0.0000;FS=0.000;MLEAC=1;MLEAF=0.500;MQ=60.00;QD=29.40;SOR=1.609	GT:AD:DP:GQ:PGT:PID:PL:PS	1|1:0,1:1:3:1|1:3758_T_TCTCGGCTGG:45,3,0:3758	BF.7	no
3767	A	G	35.48	PA	AC=2;AF=1.00;AN=2;DP=1;ExcessHet=0.0000;FS=0.0	GT:A	1|1:0,1:1:3:1|	BF.7	no

				SS	00;MLEAC=1;MLEAF=0.500;MQ=60.00;QD=36.76;SOR=1.609	D:DP:GQ:PGT:PID:PL:PS	1:3758_T_TCTCGGCTGG:45,3,0:3758		
3821	C	A	31.64	PASS	AC=1;AF=0.500;AN=2;DP=2;ExcessHet=0.0000;FS=0.000;MLEAC=1;MLEAF=0.500;MQ=60.00;MQRankSum=0.00;QD=15.82;SOR=0.693	GT:AD:DP:GQ:PGT:PID:PL:PS	0|1:1,1:2:39:0|1:3818_A_AATCT:39,0,39:3818	BF.7	no
4057	T	C	456.06	PASS	AC=2;AF=1.00;AN=2;BaseQRankSum=0.00;DP=21;ExcessHet=0.0000;FS=0.000;MLEAC=2;MLEAF=1.00;MQ=60.00;MQRankSum=0.00;QD=21.72;ReadPosRankSum=-1.079e+00;SOR=0.223	GT:AD:DP:GQ:PL	1/1:1,20:21:38:470,38,0	BF.7	no
4120	T	C	106593.1	PASS	AC=2;AF=1.00;AN=2;BaseQRankSum=0.721;DP=4035;ExcessHet=0.0000;FS=0.000;MLEAC=2;MLEAF=1.00;MQ=60.00;MQRankSum=0.00;QD=27.55;ReadPosRankSum=2.69;SOR=0.597	GT:AD:DP:GQ:PL	1/1:306,3563:3869:99:106607,1706,0	BF.7	no
4184	G	A	206678.1	PASS	AC=2;AF=1.00;AN=2;BaseQRankSum=-1.870e+00;DP=5580;ExcessHet=0.0000;FS=0.000;MLEAC=2;MLEAF=1.00;MQ=60.00;MQRankSum=0.00;QD=28.17;ReadPosRankSum=3.82;SOR=0.028	GT:AD:DP:GQ:PL	1/1:31,2980:3027:99:206692,14784,0	BF.7	yes
4321	C	T	205751.1	PASS	AC=2;AF=1.00;AN=2;BaseQRankSum=1.43;DP=5569;ExcessHet=0.0000;FS=0.000;MLEAC=2;MLEAF=1.00;MQ=60.00;MQRankSum=-	GT:AD:DP:	1/1:25,5383:5408:99:2057	BF.7	yes

					1.460e-01;QD=26.80;ReadPosRankSum=7.94;SOR=0.769	GQ:PL	65,15449,0		
4901	C	T	35.48	PASS	AC=2;AF=1.00;AN=2;DP=1;ExcessHet=0.0000;FS=0.000;MLEAC=1;MLEAF=0.500;MQ=60.00;QD=34.17;SOR=1.609	GT:AD:DP:GQ:PGT:PID:PL:PS	1|1:0,1:1:3:1|1:4901_C_T:45,3,0:4901	BF.7	no
4905	A	C	35.48	PASS	AC=2;AF=1.00;AN=2;DP=1;ExcessHet=0.0000;FS=0.000;MLEAC=1;MLEAF=0.500;MQ=60.00;QD=28.60;SOR=1.609	GT:AD:DP:GQ:PGT:PID:PL:PS	1|1:0,1:1:3:1|1:4901_C_T:45,3,0:4901	BF.7	no
5330	A	C	49.64	PASS	AC=1;AF=0.500;AN=2;BaseQRankSum=-2.300e-01;DP=11;ExcessHet=0.0000;FS=0.000;MLEAC=1;MLEAF=0.500;MQ=60.00;MQRankSum=0.00;QD=4.51;ReadPosRankSum=-2.362e+00;SOR=0.527	GT:AD:DP:GQ:PGT:PID:PL:PS	0|1:9,2:11:57:0|1:5325_A_AATTACTTGCAAT:57,0,372:5325	BF.7	no
5512	C	T	225059.1	PASS	AC=2;AF=1.00;AN=2;BaseQRankSum=-3.600e-01;DP=5811;ExcessHet=0.0000;FS=0.000;MLEAC=2;MLEAF=1.00;MQ=60.00;MQRankSum=0.00;QD=26.80;ReadPosRankSum=1.54;SOR=0.664	GT:AD:DP:GQ:PL	1/1:3,5540:5549:99:225073,16748,0	BF.7	yes
5628	C	T	753.64	PASS	AC=1;AF=0.500;AN=2;BaseQRankSum=2.72;DP=106;ExcessHet=0.0000;FS=0.772;MLEAC=1;MLEAF=0.500;MQ=60.00;MQRankSum=	GT:AD:DP:	0/1:65,37:102:99:761,0,1355	BF.7	no

					0.00;QD=7.39;ReadPosRankSum=-1.250e-01;SOR=0.665	GQ:PL			
5695	A	T	73.64	PASS	AC=1;AF=0.500;AN=2;BaseQRankSum=0.00;DP=3;ExcessHet=0.0000;FS=0.000;MLEAC=1;MLEAF=0.500;MQ=60.00;MQRankSum=0.00;QD=24.55;ReadPosRankSum=0.967;SOR=0.223	GT:AD:DP:GQ:PGT:PID:PL:PS	0|1:1,2:3:36:0|1:5695_A_T:81,0,36:5695	BF.7	no
5697	C	A	73.64	PASS	AC=1;AF=0.500;AN=2;BaseQRankSum=0.00;DP=3;ExcessHet=0.0000;FS=0.000;MLEAC=1;MLEAF=0.500;MQ=60.00;MQRankSum=0.00;QD=24.55;ReadPosRankSum=0.967;SOR=0.223	GT:AD:DP:GQ:PGT:PID:PL:PS	0|1:1,2:3:36:0|1:5695_A_T:81,0,36:5695	BF.7	no
5842	C	T	224040.1	PASS	AC=2;AF=1.00;AN=2;BaseQRankSum=-4.610e-01;DP=5985;ExcessHet=0.0000;FS=0.000;MLEAC=2;MLEAF=1.00;MQ=60.00;MQRankSum=0.00;QD=26.80;ReadPosRankSum=1.68;SOR=1.079	GT:AD:DP:GQ:PL	1/1:1,3954:4052:99:224054,17182,0	BF.7	yes
6268	C	A	107.64	PASS	AC=1;AF=0.500;AN=2;BaseQRankSum=0.00;DP=8;ExcessHet=0.0000;FS=0.000;MLEAC=1;MLEAF=0.500;MQ=60.00;MQRankSum=0.00;QD=13.45;ReadPosRankSum=-2.450e+00;SOR=0.693	GT:AD:DP:GQ:PGT:PID:PL:PS	0|1:4,4:8:99:0|1:6266_G_GA:115,0,156:6266	BF.7	no
6404	G	T	145.64	PASS	AC=1;AF=0.500;AN=2;BaseQRankSum=-5.890e-01;DP=9;ExcessHet=0.0000;FS=0.000;MLEAC=1;MLEAF=0.500;MQ=60.00;MQR	GT:AD:DP:GQ	0|1:5,4:9:99:0|1:6404_G_T:153,	BF.7	no

<table>
<tr><td></td><td></td><td></td><td></td><td></td><td></td><td></td><td></td><td></td><td></td></tr>
<tr><td></td><td></td><td></td><td></td><td></td><td>ankSum=0.00;QD=16.18;ReadPosRankSum=-1.803e+00;SOR=0.446</td><td>:PGT:PID:PL:PS</td><td>0,198:6404</td><td></td><td></td></tr>
<tr><td>6407</td><td>T</td><td>G</td><td>136.64</td><td>PASS</td><td>AC=1;AF=0.500;AN=2;BaseQRankSum=-5.890e-01;DP=12;ExcessHet=0.0000;FS=0.000;MLEAC=1;MLEAF=0.500;MQ=60.00;MQRankSum=0.00;QD=11.39;ReadPosRankSum=-1.803e+00;SOR=0.368</td><td>GT:AD:DP:GQ:PGT:PID:PL:PS</td><td>0|1:8,4:12:99:0|1:6404_G_T:144,0,324:6404</td><td>BF.7</td><td>no</td></tr>
<tr><td>6411</td><td>A</td><td>T</td><td>136.64</td><td>PASS</td><td>AC=1;AF=0.500;AN=2;BaseQRankSum=-7.990e-01;DP=12;ExcessHet=0.0000;FS=0.000;MLEAC=1;MLEAF=0.500;MQ=60.00;MQRankSum=0.00;QD=11.39;ReadPosRankSum=-1.169e+00;SOR=0.368</td><td>GT:AD:DP:GQ:PGT:PID:PL:PS</td><td>0|1:8,4:12:99:0|1:6404_G_T:144,0,324:6404</td><td>BF.7</td><td>no</td></tr>
<tr><td>6414</td><td>A</td><td>C</td><td>136.64</td><td>PASS</td><td>AC=1;AF=0.500;AN=2;BaseQRankSum=-7.990e-01;DP=12;ExcessHet=0.0000;FS=0.000;MLEAC=1;MLEAF=0.500;MQ=60.00;MQRankSum=0.00;QD=11.39;ReadPosRankSum=-3.087e+00;SOR=0.368</td><td>GT:AD:DP:GQ:PGT:PID:PL:PS</td><td>0|1:8,4:12:99:0|1:6404_G_T:144,0,324:6404</td><td>BF.7</td><td>no</td></tr>
<tr><td>6729</td><td>A</td><td>G</td><td>145.64</td><td>PASS</td><td>AC=1;AF=0.500;AN=2;BaseQRankSum=-1.085e+00;DP=9;ExcessHet=0.0000;FS=0.000;MLEAC=1;MLEAF=0.500;MQ=60.00;MQRankSum=0.00;QD=16.18;ReadPosRankSum=-1.803e+00;SOR=0.446</td><td>GT:AD:DP:GQ:PGT:PID:PL:PS</td><td>0|1:5,4:9:99:0|1:6718_T_TAGTTGTACTA:153,0,198:6718</td><td>BF.7</td><td>no</td></tr>
</table>

6730	C	T	375.74	PASS	AC=2;AF=1.00;AN=2;BaseQRankSum=0.514;DP=21;ExcessHet=0.0000;FS=0.000;MLEAC=1;MLEAF=0.500;MQ=60.00;MQRankSum=0.00;QD=19.78;ReadPosRankSum=-1.951e+00;SOR=0.804	GT:AD:DP:GQ:PL	1/1:2,17:19:7:388,7,0	BF.7	no
6779	T	A	47.08	PASS	AC=1;AF=0.500;AN=2;DP=2;ExcessHet=0.0000;FS=0.000;MLEAC=1;MLEAF=0.500;MQ=60.00;QD=30.36;SOR=0.693	GT:AD:DP:GQ:PL	0/1:0,1:2:14:39,0,14	BF.7	no
6785	A	G	52.64	PASS	AC=1;AF=0.500;AN=2;BaseQRankSum=-8.440e-01;DP=25;ExcessHet=0.0000;FS=0.000;MLEAC=1;MLEAF=0.500;MQ=60.00;MQRankSum=0.00;QD=2.11;ReadPosRankSum=0.378;SOR=1.179	GT:AD:DP:GQ:PL	0/1:22,3:25:60:60,0,889	BF.7	no
6850	G	A	12810.64	PASS	AC=1;AF=0.500;AN=2;BaseQRankSum=1.27;DP=4752;ExcessHet=0.0000;FS=1.533;MLEAC=1;MLEAF=0.500;MQ=60.00;MQRankSum=0.00;QD=2.83;ReadPosRankSum=-5.467e+00;SOR=0.842	GT:AD:DP:GQ:PL	0/1:3787,738:4525:99:12818,0,120599	BF.7	no
7058	G	A	4775.06	PASS	AC=2;AF=1.00;AN=2;BaseQRankSum=0.696;DP=198;ExcessHet=0.0000;FS=0.000;MLEAC=2;MLEAF=1.00;MQ=60.00;MQRankSum=0.00;QD=27.44;ReadPosRankSum=-1.208e+00;SOR=0.693	GT:AD:DP:GQ:PL	1/1:2,172:174:99:4789,472,0	BF.7	no
7528	C	T	231352.1	PASS	AC=2;AF=1.00;AN=2;BaseQRankSum=0.838;DP=6152;ExcessHet=0.0000;FS=0.000;MLEAC=2;MLEAF=1.00;MQ=60.00;MQRankSum=0.033;QD=26.00;ReadPosRankSum=6.57;SOR=0.658	GT:AD:DP:GQ:PL	1/1:99,4495:4606:99:231366,14088,0	BF.7	yes

<table>
<tr><td>7782</td><td>A</td><td>G</td><td>2502.06</td><td>PASS</td><td>AC=2;AF=1.00;AN=2;DP=90;ExcessHet=0.0000;FS=0.000;MLEAC=2;MLEAF=1.00;MQ=60.00;QD=29.44;SOR=0.979</td><td>GT:AD:DP:GQ:PL</td><td>1/1:0,85:85:99:2516,254,0</td><td>BF.7</td><td>yes</td></tr>
<tr><td>7840</td><td>C</td><td>A</td><td>78.32</td><td>PASS</td><td>AC=2;AF=1.00;AN=2;DP=2;ExcessHet=0.0000;FS=0.000;MLEAC=1;MLEAF=0.500;MQ=60.00;QD=35.18;SOR=0.693</td><td>GT:AD:DP:GQ:PGT:PID:PL:PS</td><td>1|1:0,2:2:6:1|1:7831_TA_T:90,6,0:7831</td><td>BF.7</td><td>no</td></tr>
<tr><td>7969</td><td>T</td><td>A</td><td>78.32</td><td>PASS</td><td>AC=2;AF=1.00;AN=2;DP=4;ExcessHet=0.0000;FS=0.000;MLEAC=1;MLEAF=0.500;MQ=60.00;QD=34.86;SOR=0.693</td><td>GT:AD:DP:GQ:PGT:PID:PL:PS</td><td>1|1:0,2:2:6:1|1:7969_T_A:90,6,0:7969</td><td>BF.7</td><td>no</td></tr>
<tr><td>7972</td><td>A</td><td>C</td><td>78.32</td><td>PASS</td><td>AC=2;AF=1.00;AN=2;DP=2;ExcessHet=0.0000;FS=0.000;MLEAC=1;MLEAF=0.500;MQ=60.00;QD=29.05;SOR=0.693</td><td>GT:AD:DP:GQ:PGT:PID:PL:PS</td><td>1|1:0,2:2:6:1|1:7969_T_A:90,6,0:7969</td><td>BF.7</td><td>no</td></tr>
<tr><td>7998</td><td>C</td><td>A</td><td>73.64</td><td>PASS</td><td>AC=1;AF=0.500;AN=2;BaseQRankSum=0.00;DP=3;ExcessHet=0.0000;FS=0.000;MLEAC=1;MLEAF=0.500;MQ=60.00;MQRankSum=0.00;QD=24.55;ReadPosRankSum=0.00;SOR=0.223</td><td>GT:AD:DP:GQ:PGT:PID:PL:PS</td><td>0|1:1,2:3:36:0|1:7996_G_GGGATTGGTT:81,0,36:7996</td><td>BF.7</td><td>no</td></tr>
</table>

8226	G	T	78.32	PASS	AC=2;AF=1.00;AN=2;DP=2;ExcessHet=0.0000;FS=0.000;MLEAC=1;MLEAF=0.500;MQ=60.00;QD=30.31;SOR=0.693	GT:AD:DP:GQ:PGT:PID:PL:PS	1|1:0,2:2:6:1|1:8226_G_T:90,6,0:8226	BF.7	no
8227	T	A	78.32	PASS	AC=2;AF=1.00;AN=2;DP=2;ExcessHet=0.0000;FS=0.000;MLEAC=1;MLEAF=0.500;MQ=60.00;QD=29.84;SOR=0.693	GT:AD:DP:GQ:PGT:PID:PL:PS	1|1:0,2:2:6:1|1:8226_G_T:90,6,0:8226	BF.7	no
9344	C	T	216687.1	PASS	AC=2;AF=1.00;AN=2;DP=5481;ExcessHet=0.0000;FS=0.000;MLEAC=2;MLEAF=1.00;MQ=60.00;QD=30.02;SOR=0.945	GT:AD:DP:GQ:PL	1/1:0,5322:5322:99:216701,16006,0	BF.7	yes
9424	A	G	210741.1	PASS	AC=2;AF=1.00;AN=2;BaseQRankSum=0.984;DP=5328;ExcessHet=0.0000;FS=0.000;MLEAC=2;MLEAF=1.00;MQ=60.00;MQRankSum=-1.620e-01;QD=31.98;ReadPosRankSum=2.64;SOR=0.022	GT:AD:DP:GQ:PL	1/1:15,5125:5140:99:210755,14900,0	BF.7	yes
9534	C	T	209542.1	PASS	AC=2;AF=1.00;AN=2;BaseQRankSum=1.48;DP=5597;ExcessHet=0.0000;FS=0.000;MLEAC=2;MLEAF=1.00;MQ=60.00;MQRankSum=0.00;QD=27.51;ReadPosRankSum=1.67;SOR=0.733	GT:AD:DP:GQ:PL	1/1:2,5408:5415:99:209556,16206,0	BF.7	yes
10029	C	T	225466.1	PASS	AC=2;AF=1.00;AN=2;BaseQRankSum=-1.400e+00;DP=5887;ExcessHet=0.0000;FS=0.000;MLEAC=2;MLEAF=1.00;MQ=6	GT:AD:DP:	1/1:3,5696:5699:99:22548	BF.7	yes

					0.00;MQRankSum=0.00;QD=29.11;ReadPosRankSum=1.78;SOR=0.398	GQ:PL	0,16996,0		
10162	T	G	73.64	PASS	AC=1;AF=0.500;AN=2;BaseQRankSum=0.00;DP=3;ExcessHet=0.0000;FS=0.000;MLEAC=1;MLEAF=0.500;MQ=60.00;MQRankSum=0.00;QD=24.55;ReadPosRankSum=0.967;SOR=0.223	GT:AD:DP:GQ:PGT:PID:PL:PS	0|1:1,2:3:36:0|1:10157_GTA_G:81,0,36:10157	BF.7	no
10198	C	T	234521.1	PASS	AC=2;AF=1.00;AN=2;BaseQRankSum=2.76;DP=6147;ExcessHet=0.0000;FS=0.000;MLEAC=2;MLEAF=1.00;MQ=60.00;MQRankSum=-4.510e-01;QD=28.08;ReadPosRankSum=2.91;SOR=0.965	GT:AD:DP:GQ:PL	1/1:4,5899:5916:99:234535,17637,0	BF.7	yes
10447	G	A	253575.1	PASS	AC=2;AF=1.00;AN=2;DP=6092;ExcessHet=0.0000;FS=0.000;MLEAC=2;MLEAF=1.00;MQ=60.00;MQRankSum=0.254;QD=23.23;SOR=1.073	GT:AD:DP:GQ:PL	1/1:1,4318:4435:99:253589,17069,0	BF.7	yes
10449	C	A	250868.1	PASS	AC=2;AF=1.00;AN=2;BaseQRankSum=-8.140e-01;DP=5839;ExcessHet=0.0000;FS=0.000;MLEAC=2;MLEAF=1.00;MQ=60.00;MQRankSum=0.00;QD=29.03;ReadPosRankSum=5.43;SOR=0.227	GT:AD:DP:GQ:PL	1/1:31,4303:4339:99:250882,15720,0	BF.7	yes
10615	C	T	5771.06	PASS	AC=2;AF=1.00;AN=2;BaseQRankSum=0.621;DP=234;ExcessHet=0.0000;FS=0.000;MLEAC=2;MLEAF=1.00;MQ=60.00;MQRankSum=0.00;QD=26.00;ReadPosRankSum=-2.708e+00;SOR=0.396	GT:AD:DP:GQ:PL	1/1:3,219:222:99:5785,590,0	BF.7	yes
10792	T	A	78.32	PA	AC=2;AF=1.00;AN=2;DP=2;ExcessHet=0.0000;FS=0.000;MLEAC=1;MLEAF=0.5	GT:AD:	1|1:0,2:2:6:1|1:1078	BF.7	no

				SS	00;MQ=60.00;QD=28.65;SOR=0.693	DP:GQ:PGT:PID:PL:PS	7_G_GACCCCTTAC:90,6,0:10787		
10794	T	G	78.32	PASS	AC=2;AF=1.00;AN=2;DP=2;ExcessHet=0.0000;FS=0.000;MLEAC=1;MLEAF=0.500;MQ=60.00;QD=28.44;SOR=0.693	GT:AD:DP:GQ:PGT:PID:PL:PS	1|1:0,2:2:6:1|1:10787_G_GACCCCTTAC:90,6,0:10787	BF.7	no
10798	C	A	78.32	PASS	AC=2;AF=1.00;AN=2;DP=2;ExcessHet=0.0000;FS=0.000;MLEAC=1;MLEAF=0.500;MQ=60.00;QD=35.95;SOR=0.693	GT:AD:DP:GQ:PGT:PID:PL:PS	1|1:0,2:2:6:1|1:10787_G_GACCCCTTAC:90,6,0:10787	BF.7	no
11202	C	G	58.64	PASS	AC=1;AF=0.500;AN=2;BaseQRankSum=-3.660e-01;DP=9;ExcessHet=0.0000;FS=0.000;MLEAC=1;MLEAF=0.500;MQ=60.00;MQRankSum=0.00;QD=7.33;ReadPosRankSum=-2.100e+00;SOR=0.307	GT:AD:DP:GQ:PGT:PID:PL:PS	0|1:6,2:8:66:0|1:11202_C_G:66,0,246:11202	BF.7	no
11224	C	A	30.48	PASS	AC=2;AF=1.00;AN=2;DP=2;ExcessHet=0.0000;FS=0.000;MLEAC=1;MLEAF=0.500;MQ=60.00;QD=30.48;SOR=1.609	GT:AD:DP:GQ:PGT:PID:	1|1:0,1:1:3:1|1:11221_G_GAATGTGTA:40,3,0:11221	BF.7	no

						PL:PS			
11831	G	C	78.32	PASS	AC=2;AF=1.00;AN=2;DP=2;ExcessHet=0.0000;FS=0.000;MLEAC=1;MLEAF=0.500;MQ=60.00;QD=28.50;SOR=0.693	GT:AD:DP:GQ:PGT:PID:PL:PS	1|1:0,2:2:6:1|1:11831_G_C:90,6,0:11831	BF.7	no
11859	T	G	35.48	PASS	AC=2;AF=1.00;AN=2;DP=1;ExcessHet=0.0000;FS=0.000;MLEAC=1;MLEAF=0.500;MQ=60.00;QD=26.72;SOR=1.609	GT:AD:DP:GQ:PGT:PID:PL:PS	1|1:0,1:1:3:1|1:11859_T_G:45,3,0:11859	BF.7	no
11860	A	C	35.48	PASS	AC=2;AF=1.00;AN=2;DP=1;ExcessHet=0.0000;FS=0.000;MLEAC=1;MLEAF=0.500;MQ=60.00;QD=24.84;SOR=1.609	GT:AD:DP:GQ:PGT:PID:PL:PS	1|1:0,1:1:3:1|1:11859_T_G:45,3,0:11859	BF.7	no
11863	G	T	35.48	PASS	AC=2;AF=1.00;AN=2;DP=1;ExcessHet=0.0000;FS=0.000;MLEAC=1;MLEAF=0.500;MQ=60.00;QD=25.31;SOR=1.609	GT:AD:DP:GQ:PGT:PID:PL:PS	1|1:0,1:1:3:1|1:11859_T_G:45,3,0:11859	BF.7	no
12005	C	A	31.64	PASS	AC=1;AF=0.500;AN=2;BaseQRankSum=0.00;DP=2;ExcessHet=0.0000;FS=0.000;MLEAC=1;MLEAF=0.500;	GT:AD:DP:	0|1:1,1:2:39:0|1:12005_C	BF.7	no

					MQ=60.00;MQRankSum=0.00;QD=15.82;ReadPosRankSum=0.674;SOR=0.693	GQ:PGT:PID:PL:PS	_A:39,0,39:12005		
12006	T	C	31.64	PASS	AC=1;AF=0.500;AN=2;DP=2;ExcessHet=0.0000;FS=0.000;MLEAC=1;MLEAF=0.500;MQ=60.00;MQRankSum=0.00;QD=15.82;SOR=0.693	GT:AD:DP:GQ:PGT:PID:PL:PS	0|1:1,1:2:39:0|1:12005_C_A:39,0,39:12005	BF.7	no
12009	T	C	35.48	PASS	AC=2;AF=1.00;AN=2;DP=1;ExcessHet=0.0000;FS=0.000;MLEAC=1;MLEAF=0.500;MQ=60.00;QD=31.63;SOR=1.609	GT:AD:DP:GQ:PGT:PID:PL:PS	1|1:0,1:1:3:1|1:12005_C_A:45,3,0:12005	BF.7	no
12160	G	A	236027.1	PASS	AC=2;AF=1.00;AN=2;BaseQRankSum=1.39;DP=6233;ExcessHet=0.0000;FS=0.000;MLEAC=2;MLEAF=1.00;MQ=60.00;MQRankSum=-2.220e-01;QD=23.23;ReadPosRankSum=4.35;SOR=0.066	GT:AD:DP:GQ:PL	1/1:17,5957:5976:99:236041,17336,0	BF.7	yes
12430	T	C	35.48	PASS	AC=2;AF=1.00;AN=2;DP=1;ExcessHet=0.0000;FS=0.000;MLEAC=1;MLEAF=0.500;MQ=60.00;QD=32.70;SOR=1.609	GT:AD:DP:GQ:PGT:PID:PL:PS	1|1:0,1:1:3:1|1:12428_G_GAATTA:45,3,0:12428	BF.7	no

12640	T	A	35.48	PASS	AC=2;AF=1.00;AN=2;DP=1;ExcessHet=0.0000;FS=0.000;MLEAC=1;MLEAF=0.500;MQ=60.00;QD=29.96;SOR=1.609	GT:AD:DP:GQ:PGT:PID:PL:PS	1|1:0,1:1:3:1|1:12638_C_CTT:45,3,0:12638	BF.7	no
12697	T	A	35.48	PASS	AC=2;AF=1.00;AN=2;DP=1;ExcessHet=0.0000;FS=0.000;MLEAC=1;MLEAF=0.500;MQ=60.00;QD=29.19;SOR=1.609	GT:AD:DP:GQ:PGT:PID:PL:PS	1|1:0,1:1:3:1|1:12695_C_CACACAGAGTCTA:45,3,0:12695	BF.7	no
12844	G	T	67.64	PASS	AC=1;AF=0.500;AN=2;BaseQRankSum=0.00;DP=8;ExcessHet=0.0000;FS=0.000;MLEAC=1;MLEAF=0.500;MQ=60.00;MQRankSum=0.00;QD=8.45;ReadPosRankSum=0.816;SOR=0.693	GT:AD:DP:GQ:PL	0/1:4,4:8:75:75,0,75	BF.7	yes
12880	C	T	188876.1	PASS	AC=2;AF=1.00;AN=2;BaseQRankSum=-1.492e+00;DP=5286;ExcessHet=0.0000;FS=0.000;MLEAC=2;MLEAF=1.00;MQ=60.00;MQRankSum=0.301;QD=29.20;ReadPosRankSum=2.40;SOR=1.006	GT:AD:DP:GQ:PL	1/1:4,5070:5074:99:188890,15064,0	BF.7	yes
13173	G	A	35.48	PASS	AC=2;AF=1.00;AN=2;DP=1;ExcessHet=0.0000;FS=0.000;MLEAC=1;MLEAF=0.500;MQ=60.00;QD=30.34;SOR=1.609	GT:AD:DP:GQ:PGT:PID:PL:PS	1|1:0,1:1:3:1|1:13171_T_TATAGAA:45,3,0:13171	BF.7	no

13339	T	G	770.64	PASS	AC=1;AF=0.500;AN=2;BaseQRankSum=0.348;DP=287;ExcessHet=0.0000;FS=1.797;MLEAC=1;MLEAF=0.500;MQ=60.00;MQRankSum=0.00;QD=2.71;ReadPosRankSum=1.34;SOR=0.861	GT:AD:DP:GQ:PL	0/1:225,59:284:99:778,0,5323	BF.7	no
13540	G	A	58.64	PASS	AC=1;AF=0.500;AN=2;BaseQRankSum=-2.100e+00;DP=9;ExcessHet=0.0000;FS=0.000;MLEAC=1;MLEAF=0.500;MQ=60.00;MQRankSum=0.00;QD=7.33;ReadPosRankSum=-1.242e+00;SOR=0.307	GT:AD:DP:GQ:PGT:PID:PL:PS	0|1:6,2:8:66:0|1:13540_G_A:66,0,246:13540	BF.7	no
13546	G	A	58.64	PASS	AC=1;AF=0.500;AN=2;BaseQRankSum=-2.100e+00;DP=8;ExcessHet=0.0000;FS=0.000;MLEAC=1;MLEAF=0.500;MQ=60.00;MQRankSum=0.00;QD=7.33;ReadPosRankSum=-1.242e+00;SOR=0.307	GT:AD:DP:GQ:PGT:PID:PL:PS	0|1:6,2:8:66:0|1:13540_G_A:66,0,246:13540	BF.7	no
13547	A	C	58.64	PASS	AC=1;AF=0.500;AN=2;BaseQRankSum=-2.100e+00;DP=8;ExcessHet=0.0000;FS=0.000;MLEAC=1;MLEAF=0.500;MQ=60.00;MQRankSum=0.00;QD=7.33;ReadPosRankSum=-2.100e+00;SOR=0.307	GT:AD:DP:GQ:PGT:PID:PL:PS	0|1:6,2:8:66:0|1:13540_G_A:66,0,246:13540	BF.7	no
13549	A	T	58.64	PASS	AC=1;AF=0.500;AN=2;BaseQRankSum=-2.100e+00;DP=8;ExcessHet=0.0000;FS=0.000;MLEAC=1;MLEAF=0.500;MQ=60.00;MQRankSum=0.00;QD=7.33;ReadPosRankSum=-2.100e+00;SOR=0.307	GT:AD:DP:GQ:PGT:PID:	0|1:6,2:8:66:0|1:13540_G_A:66,0,246:13540	BF.7	no

<table>
<tr><th></th><th></th><th></th><th></th><th></th><th></th><th></th><th></th><th></th><th></th></tr>
<tr><td></td><td></td><td></td><td></td><td></td><td></td><td>PL:PS</td><td></td><td></td><td></td></tr>
<tr><td>14126</td><td>G</td><td>A</td><td>22518.64</td><td>PASS</td><td>AC=1;AF=0.500;AN=2;BaseQRankSum=-3.358e+00;DP=6062;ExcessHet=0.0000;FS=0.000;MLEAC=1;MLEAF=0.500;MQ=60.00;MQRankSum=0.00;QD=3.82;ReadPosRankSum=-1.090e+00;SOR=0.712</td><td>GT:AD:DP:GQ:PL</td><td>0/1:4834,1068:5902:99:22526,0,168156</td><td>BF.7</td><td>no</td></tr>
<tr><td>14181</td><td>G</td><td>C</td><td>193426.1</td><td>PASS</td><td>AC=2;AF=1.00;AN=2;DP=5320;ExcessHet=0.0000;FS=0.000;MLEAC=2;MLEAF=1.00;MQ=60.00;QD=30.67;SOR=1.242</td><td>GT:AD:DP:GQ:PL</td><td>1/1:0,2777:2904:99:193440,15204,0</td><td>BF.7</td><td>yes</td></tr>
<tr><td>14265</td><td>C</td><td>T</td><td>4706.06</td><td>PASS</td><td>AC=2;AF=1.00;AN=2;BaseQRankSum=-5.540e-01;DP=179;ExcessHet=0.0000;FS=2.147;MLEAC=2;MLEAF=1.00;MQ=60.00;MQRankSum=0.00;QD=27.36;ReadPosRankSum=-2.690e-01;SOR=0.484</td><td>GT:AD:DP:GQ:PL</td><td>1/1:3,169:172:99:4720,428,0</td><td>BF.7</td><td>no</td></tr>
<tr><td>14408</td><td>C</td><td>T</td><td>211646.1</td><td>PASS</td><td>AC=2;AF=1.00;AN=2;BaseQRankSum=-1.810e-01;DP=5754;ExcessHet=0.0000;FS=0.000;MLEAC=2;MLEAF=1.00;MQ=60.00;MQRankSum=0.285;QD=28.55;ReadPosRankSum=0.849;SOR=0.577</td><td>GT:AD:DP:GQ:PL</td><td>1/1:8,5449:5525:99:211660,16129,0</td><td>BF.7</td><td>yes</td></tr>
<tr><td>14977</td><td>A</td><td>G</td><td>35.48</td><td>PASS</td><td>AC=2;AF=1.00;AN=2;DP=1;ExcessHet=0.0000;FS=0.000;MLEAC=1;MLEAF=0.500;MQ=60.00;QD=26.13;SOR=1.609</td><td>GT:AD:DP:GQ:PGT:PID:PL:PS</td><td>1|1:0,1:1:3:1|1:14977_A_G:45,3,0:14977</td><td>BF.7</td><td>no</td></tr>
<tr><td>14982</td><td>T</td><td>A</td><td>35.48</td><td>PASS</td><td>AC=2;AF=1.00;AN=2;DP=1;ExcessHet=0.0000;FS=0.000;MLEAC=1;MLEAF=0.5</td><td>GT:AD:DP:</td><td>1|1:0,1:1:3:1|1:14977_A_</td><td>BF.7</td><td>no</td></tr>
</table>

<table>
<tr><th></th><th></th><th></th><th></th><th></th><th></th><th></th><th></th><th></th><th></th></tr>
<tr><td></td><td></td><td></td><td></td><td></td><td>00;MQ=60.00;QD=29.87;SOR=1.609</td><td>GQ:PGT:PID:PL:PS</td><td>G:45,3,0:14977</td><td></td><td></td></tr>
<tr><td>15444</td><td>G</td><td>T</td><td>213283.1</td><td>PASS</td><td>AC=2;AF=1.00;AN=2;BaseQRankSum=2.06;DP=5790;ExcessHet=0.0000;FS=0.000;MLEAC=2;MLEAF=1.00;MQ=60.00;MQRankSum=0.00;QD=26.61;ReadPosRankSum=2.03;SOR=0.811</td><td>GT:AD:DP:GQ:PL</td><td>1/1:2,5584:5591:99:213297,16748,0</td><td>BF.7</td><td>no</td></tr>
<tr><td>15451</td><td>G</td><td>A</td><td>982.06</td><td>PASS</td><td>AC=2;AF=1.00;AN=2;DP=38;ExcessHet=0.0000;FS=0.000;MLEAC=2;MLEAF=1.00;MQ=60.00;QD=26.54;SOR=0.746</td><td>GT:AD:DP:GQ:PL</td><td>1/1:0,37:37:99:996,111,0</td><td>BF.7</td><td>no</td></tr>
<tr><td>15714</td><td>C</td><td>T</td><td>228944.1</td><td>PASS</td><td>AC=2;AF=1.00;AN=2;BaseQRankSum=2.73;DP=6107;ExcessHet=0.0000;FS=0.000;MLEAC=2;MLEAF=1.00;MQ=60.00;MQRankSum=0.00;QD=34.42;ReadPosRankSum=2.84;SOR=0.631</td><td>GT:AD:DP:GQ:PL</td><td>1/1:3,5254:5257:99:228958,17602,0</td><td>BF.7</td><td>yes</td></tr>
<tr><td>15865</td><td>C</td><td>A</td><td>35.48</td><td>PASS</td><td>AC=2;AF=1.00;AN=2;DP=1;ExcessHet=0.0000;FS=0.000;MLEAC=1;MLEAF=0.500;MQ=60.00;QD=28.89;SOR=1.609</td><td>GT:AD:DP:GQ:PGT:PID:PL:PS</td><td>1|1:0,1:1:3:1|1:15856_ACT_A:45,3,0:15856</td><td>BF.7</td><td>no</td></tr>
<tr><td>15932</td><td>A</td><td>T</td><td>35.48</td><td>PASS</td><td>AC=2;AF=1.00;AN=2;DP=1;ExcessHet=0.0000;FS=0.000;MLEAC=1;MLEAF=0.500;MQ=60.00;QD=32.35;SOR=1.609</td><td>GT:AD:DP:GQ:PGT:PID:PL:PS</td><td>1|1:0,1:1:3:1|1:15927_T_TTGAACATG:45,3,0:15927</td><td>BF.7</td><td>no</td></tr>
</table>

16092	C	T	35.48	PASS	AC=2;AF=1.00;AN=2;DP=1;ExcessHet=0.0000;FS=0.000;MLEAC=1;MLEAF=0.500;MQ=60.00;QD=29.61;SOR=1.609	GT:AD:DP:GQ:PGT:PID:PL:PS	1|1:0,1:1:3:1|1:16089_G_GTCTGGCT:45,3,0:16089	BF.7	no
16482	A	G	11947.06	PASS	AC=2;AF=1.00;AN=2;BaseQRankSum=1.29;DP=470;ExcessHet=0.0000;FS=0.000;MLEAC=2;MLEAF=1.00;MQ=60.00;MQRankSum=0.00;QD=26.14;ReadPosRankSum=1.72;SOR=0.698	GT:AD:DP:GQ:PL	1/1:20,437:457:99:11961,841,0	BF.7	no
16935	G	T	103.64	PASS	AC=1;AF=0.500;AN=2;BaseQRankSum=0.417;DP=39;ExcessHet=0.0000;FS=1.854;MLEAC=1;MLEAF=0.500;MQ=60.00;MQRankSum=0.00;QD=2.73;ReadPosRankSum=-1.823e+00;SOR=0.911	GT:AD:DP:GQ:PGT:PID:PL:PS	0|1:33,5:38:99:0|1:16935_G_T:111,0,1290:16935	BF.7	no
16986	T	A	78.31	PASS	AC=2;AF=1.00;AN=2;DP=2;ExcessHet=0.0000;FS=0.000;MLEAC=1;MLEAF=0.500;MQ=60.00;QD=31.78;SOR=0.693	GT:AD:DP:GQ:PL	1/1:0,2:2:6:90,6,0	BF.7	no
16995	C	G	78.32	PASS	AC=2;AF=1.00;AN=2;DP=2;ExcessHet=0.0000;FS=0.000;MLEAC=1;MLEAF=0.500;MQ=60.00;QD=31.99;SOR=0.693	GT:AD:DP:GQ:PL	1/1:0,2:2:6:90,6,0	BF.7	no
17130	C	T	29069.06	PASS	AC=2;AF=1.00;AN=2;BaseQRankSum=0.185;DP=1052;ExcessHet=0.0000;FS=0.000;MLEAC=2;MLEAF=1.00;MQ=60.00;MQRankSum=0.00;QD=28.67;ReadPosRankSum=0.457;SOR=0.553	GT:AD:DP:GQ:PL	1/1:5,1009:1014:99:29083,2897,0	BF.7	no

17410	C	T	222119.1	PASS	AC=2;AF=1.00;AN=2;BaseQRankSum=-3.640e-01;DP=5792;ExcessHet=0.0000;FS=0.000;MLEAC=2;MLEAF=1.00;MQ=60.00;MQRankSum=0.793;QD=28.53;ReadPosRankSum=1.64;SOR=0.441	GT:AD:DP:GQ:PL	1/1:1,5558:5562:99:222133,16718,0	BF.7	yes
17661	T	C	35.48	PASS	AC=2;AF=1.00;AN=2;DP=1;ExcessHet=0.0000;FS=0.000;MLEAC=1;MLEAF=0.500;MQ=60.00;QD=25.40;SOR=1.609	GT:AD:DP:GQ:PGT:PID:PL:PS	1|1:0,1:1:3:1|1:17658_G_GAA:45,3,0:17658	BF.7	no
17746	C	T	15469.06	PASS	AC=2;AF=1.00;AN=2;BaseQRankSum=-2.651e+00;DP=592;ExcessHet=0.0000;FS=0.000;MLEAC=2;MLEAF=1.00;MQ=60.00;MQRankSum=0.00;QD=26.76;ReadPosRankSum=0.929;SOR=0.318	GT:AD:DP:GQ:PL	1/1:1,577:580:99:15483,1691,0	BF.7	yes
17880	A	T	31.63	PASS	AC=2;AF=1.00;AN=2;DP=2;ExcessHet=0.0000;FS=0.000;MLEAC=1;MLEAF=0.500;MQ=60.00;QD=31.63;SOR=2.303	GT:AD:DP:GQ:PGT:PID:PL:PS	1|1:0,1:2:3:1|1:17880_A_*:45,3,0:17880	BF.7	no
18160	G	A	14288.06	PASS	AC=2;AF=1.00;AN=2;DP=330;ExcessHet=0.0000;FS=0.000;MLEAC=2;MLEAF=1.00;MQ=60.00;QD=27.24;SOR=0.921	GT:AD:DP:GQ:PGT:PID:PL:PS	1|1:0,328:328:99:1|1:18160_G_A:14302,987,0:18160	BF.7	no

18163	A	G	214934.1	PASS	AC=2;AF=1.00;AN=2;BaseQRankSum=0.586;DP=5681;ExcessHet=0.0000;FS=0.000;MLEAC=2;MLEAF=1.00;MQ=60.00;MQRankSum=0.00;QD=27.23;ReadPosRankSum=1.50;SOR=0.955	GT:AD:DP:GQ:PL	1/1:2,5482:5484:99:214948,16409,0	BF.7	yes
18249	T	G	34.64	PASS	AC=1;AF=0.500;AN=2;BaseQRankSum=-7.400e-01;DP=17;ExcessHet=0.0000;FS=0.000;MLEAC=1;MLEAF=0.500;MQ=60.00;MQRankSum=0.00;QD=2.16;ReadPosRankSum=-1.209e+00;SOR=0.307	GT:AD:DP:GQ:PGT:PID:PL:PS	0|1:14,2:16:42:0|1:18249_T_G:42,0,582:18249	BF.7	no
18250	A	T	34.64	PASS	AC=1;AF=0.500;AN=2;BaseQRankSum=-7.400e-01;DP=16;ExcessHet=0.0000;FS=0.000;MLEAC=1;MLEAF=0.500;MQ=60.00;MQRankSum=0.00;QD=2.16;ReadPosRankSum=-1.865e+00;SOR=0.307	GT:AD:DP:GQ:PGT:PID:PL:PS	0|1:14,2:16:42:0|1:18249_T_G:42,0,582:18249	BF.7	no
18311	T	C	94.64	PASS	AC=1;AF=0.500;AN=2;BaseQRankSum=0.00;DP=11;ExcessHet=0.0000;FS=0.000;MLEAC=1;MLEAF=0.500;MQ=60.00;MQRankSum=0.00;QD=8.60;ReadPosRankSum=-2.029e+00;SOR=1.179	GT:AD:DP:GQ:PGT:PID:PL:PS	0|1:8,3:11:99:0|1:18311_T_C:102,0,327:18311	BF.7	no
18313	G	C	94.64	PASS	AC=1;AF=0.500;AN=2;BaseQRankSum=0.00;DP=11;ExcessHet=0.0000;FS=0.000;MLEAC=1;MLEAF=0.500;MQ=60.00;MQRankSum=0.00;QD=8.60;ReadPosRankSum=-1.922e+00;SOR=1.179	GT:AD:DP:GQ:PGT:PID:	0|1:8,3:11:99:0|1:18311_T_C:102,0,327:18311	BF.7	no

						PL:PS			
18668	G	C	150.64	PASS	AC=1;AF=0.500;AN=2;BaseQRankSum=-4.300e-02;DP=55;ExcessHet=0.0000;FS=1.639;MLEAC=1;MLEAF=0.500;MQ=60.00;MQRankSum=0.00;QD=2.90;ReadPosRankSum=-1.225e+00;SOR=0.747	GT:AD:DP:GQ:PGT:PID:PL:PS	0|1:45,7:52:99:0|1:18668_G_C:158,0,1869:18668	BF.7	no
18669	T	A	150.64	PASS	AC=1;AF=0.500;AN=2;BaseQRankSum=-6.400e-02;DP=55;ExcessHet=0.0000;FS=1.639;MLEAC=1;MLEAF=0.500;MQ=60.00;MQRankSum=0.00;QD=2.90;ReadPosRankSum=-1.451e+00;SOR=0.747	GT:AD:DP:GQ:PGT:PID:PL:PS	0|1:45,7:52:99:0|1:18668_G_C:158,0,1869:18668	BF.7	no
18670	G	C	150.64	PASS	AC=1;AF=0.500;AN=2;BaseQRankSum=-6.400e-02;DP=52;ExcessHet=0.0000;FS=1.639;MLEAC=1;MLEAF=0.500;MQ=60.00;MQRankSum=0.00;QD=2.90;ReadPosRankSum=-1.775e+00;SOR=0.747	GT:AD:DP:GQ:PGT:PID:PL:PS	0|1:45,7:52:99:0|1:18668_G_C:158,0,1868:18668	BF.7	no
18696	T	C	724.64	PASS	AC=1;AF=0.500;AN=2;BaseQRankSum=1.32;DP=196;ExcessHet=0.0000;FS=2.197;MLEAC=1;MLEAF=0.500;MQ=60.00;MQRankSum=0.00;QD=4.05;ReadPosRankSum=-1.830e-01;SOR=0.672	GT:AD:DP:GQ:PL	0/1:135,44:179:99:732,0,3241	BF.7	no
19124	A	G	74.64	PASS	AC=1;AF=0.500;AN=2;BaseQRankSum=0.00;DP=22;ExcessHet=0.0000;FS=0.000;MLEAC=1;MLEAF=0.500;MQ=60.00;MQRankSum=0.	GT:AD:DP:GQ:PL	0/1:16,6:22:82:82,0,329	BF.7	no

					00;QD=3.39;ReadPosRankSum=-4.240e-01;SOR=0.693				
19132	G	T	95.64	PASS	AC=1;AF=0.500;AN=2;BaseQRankSum=-8.100e-01;DP=17;ExcessHet=0.0000;FS=2.197;MLEAC=1;MLEAF=0.500;MQ=60.00;MQRankSum=0.00;QD=5.63;ReadPosRankSum=1.09;SOR=1.402	GT:AD:DP:GQ:PGT:PID:PL:PS	0|1:13,4:17:99:0|1:19132_G_T:103,0,462:19132	BF.7	no
19137	A	C	95.64	PASS	AC=1;AF=0.500;AN=2;BaseQRankSum=-1.423e+00;DP=17;ExcessHet=0.0000;FS=2.197;MLEAC=1;MLEAF=0.500;MQ=60.00;MQRankSum=0.00;QD=5.63;ReadPosRankSum=0.789;SOR=1.402	GT:AD:DP:GQ:PGT:PID:PL:PS	0|1:13,4:17:99:0|1:19132_G_T:103,0,462:19132	BF.7	no
19679	G	T	127.61	PASS	AC=1;AF=0.500;AN=2;BaseQRankSum=-4.520e-01;DP=17;ExcessHet=0.0000;FS=5.119;MLEAC=1;MLEAF=0.500;MQ=60.00;MQRankSum=0.00;QD=8.51;ReadPosRankSum=-3.460e-01;SOR=1.944	GT:AD:DP:GQ:PGT:PID:PL:PS	0|1:11,4:16:99:0|1:19679_G_*:135,0,450:19679	BF.7	no
19680	T	A	127.58	PASS	AC=1;AF=0.500;AN=2;BaseQRankSum=0.110;DP=16;ExcessHet=0.0000;FS=5.119;MLEAC=1;MLEAF=0.500;MQ=60.00;MQRankSum=0.00;QD=8.51;ReadPosRankSum=-2.900e-01;SOR=1.944	GT:AD:DP:GQ:PGT:PID:PL:PS	0|1:11,4:16:99:0|1:19679_G_*:135,0,450:19679	BF.7	no
19859	C	T	612.06	PASS	AC=2;AF=1.00;AN=2;DP=24;ExcessHet=0.0000;FS=0.000;MLEAC=2;MLEAF=1.	GT:AD:DP:	1/1:0,22:22:66:626,66,0	BF.7	no

					00;MQ=60.00;QD=27.82;SOR=0.874	GQ:PL			
19955	C	T	230311.1	PASS	AC=2;AF=1.00;AN=2;DP=6010;ExcessHet=0.0000;FS=0.000;MLEAC=2;MLEAF=1.00;MQ=60.00;QD=28.53;SOR=0.842	GT:AD:DP:GQ:PL	1/1:0,5897:5897:99:230325,17726,0	BF.7	yes
20055	A	G	219906.1	PASS	AC=2;AF=1.00;AN=2;BaseQRankSum=3.11;DP=5956;ExcessHet=0.0000;FS=0.000;MLEAC=2;MLEAF=1.00;MQ=60.00;MQRankSum=0.00;QD=33.47;ReadPosRankSum=1.08;SOR=0.677	GT:AD:DP:GQ:PL	1/1:1,5643:5713:99:219920,16924,0	BF.7	yes
20703	C	T	142072.1	PASS	AC=2;AF=1.00;AN=2;BaseQRankSum=-4.110e-01;DP=4236;ExcessHet=0.0000;FS=0.000;MLEAC=2;MLEAF=1.00;MQ=60.00;MQRankSum=0.031;QD=34.14;ReadPosRankSum=1.31;SOR=0.660	GT:AD:DP:GQ:PL	1/1:3,4159:4162:99:142086,12419,0	BF.7	yes
21077	C	T	648.06	PASS	AC=2;AF=1.00;AN=2;DP=24;ExcessHet=0.0000;FS=0.000;MLEAC=2;MLEAF=1.00;MQ=60.00;QD=27.00;SOR=0.859	GT:AD:DP:GQ:PL	1/1:0,24:24:72:662,72,0	BF.7	no
21282	A	G	129286.1	PASS	AC=2;AF=1.00;AN=2;BaseQRankSum=1.88;DP=3840;ExcessHet=0.0000;FS=0.000;MLEAC=2;MLEAF=1.00;MQ=60.00;MQRankSum=0.00;QD=32.91;ReadPosRankSum=1.73;SOR=0.339	GT:AD:DP:GQ:PL	1/1:5,3683:3688:99:129300,10936,0	BF.7	yes
21618	C	T	195747.1	PASS	AC=2;AF=1.00;AN=2;DP=4637;ExcessHet=0.0000;FS=0.000;MLEAC=2;MLEAF=1.00;MQ=60.00;QD=32.91;SOR=0.792	GT:AD:DP:GQ:PL	1/1:0,4550:4557:99:195761,13695,0	BF.7	yes
21987	G	A	227538.1	PASS	AC=2;AF=1.00;AN=2;DP=6008;ExcessHet=0.0000;FS=0.000;MLEAC=2;MLEAF=1.00;MQ=60.00;MQRankS	GT:AD:DP:	1/1:1,5814:5817:99:22755	BF.7	yes

					um=-3.000e-02;QD=33.27;SOR=0.555	GQ:PL	2,17485,0		
22180	G	C	37.64	PASS	AC=1;AF=0.500;AN=2;BaseQRankSum=-1.960e-01;DP=15;ExcessHet=0.0000;FS=0.000;MLEAC=1;MLEAF=0.500;MQ=60.00;MQRankSum=0.00;QD=2.51;ReadPosRankSum=-2.133e+00;SOR=0.569	GT:AD:DP:GQ:PGT:PID:PL:PS	0|1:13,2:15:45:0|1:22176_C_CATTACAGCTA:45,0,540:22176	BF.7	no
22199	G	C	976.06	PASS	AC=2;AF=1.00;AN=2;DP=24;ExcessHet=0.0000;FS=0.000;MLEAC=2;MLEAF=1.00;MQ=60.00;QD=29.56;SOR=1.085	GT:AD:DP:GQ:PGT:PID:PL:PS	1|1:0,22:22:66:1|1:22199_G_C:990,66,0:22199	BF.7	no
22200	T	G	218596.1	PASS	AC=2;AF=1.00;AN=2;BaseQRankSum=3.98;DP=6018;ExcessHet=0.0000;FS=0.000;MLEAC=2;MLEAF=1.00;MQ=60.00;MQRankSum=0.270;QD=31.26;ReadPosRankSum=0.691;SOR=0.490	GT:AD:DP:GQ:PL	1/1:10,5756:5766:99:218610,17084,0	BF.7	yes
22550	C	T	1388.64	PASS	AC=1;AF=0.500;AN=2;BaseQRankSum=1.08;DP=248;ExcessHet=0.0000;FS=0.504;MLEAC=1;MLEAF=0.500;MQ=60.00;MQRankSum=0.00;QD=5.81;ReadPosRankSum=3.80;SOR=0.622	GT:AD:DP:GQ:PL	0/1:160,79:239:99:1396,0,3510	BF.7	no
22560	C	A	53.64	PASS	AC=1;AF=0.500;AN=2;BaseQRankSum=0.00;DP=21;ExcessHet=0.0000;FS=0.000;MLEAC=1;MLEAF=0.500;MQ=60.00;MQRankSum=0.00;QD=2.55;ReadPosRankSum=-8.230e-01;SOR=1.022	GT:AD:DP:GQ:PL	0/1:16,5:21:61:61,0,373	BF.7	no
22578	G	A	2123	PA	AC=2;AF=1.00;AN=2;BaseQRankSum=3.47;DP=4966;ExcessHet=0.0000;FS=0.00	GT:AD:	1/1:16,4844:4860:9	BF.7	yes

			73.1	SS	0;MLEAC=2;MLEAF=1.00;MQ=60.00;MQRankSum=-1.240e-01;QD=27.76;ReadPosRankSum=2.14;SOR=0.419	DP:GQ:PL	9:212387,13987,0		
22599	G	C	196262.1	PASS	AC=2;AF=1.00;AN=2;BaseQRankSum=0.095;DP=4639;ExcessHet=0.0000;FS=0.000;MLEAC=2;MLEAF=1.00;MQ=60.00;MQRankSum=0.171;QD=31.26;ReadPosRankSum=0.920;SOR=0.513	GT:AD:DP:GQ:PL	1/1:4,4570:4574:99:196276,13610,0	BF.7	yes
22667	T	C	101.64	PASS	AC=1;AF=0.500;AN=2;BaseQRankSum=-6.150e-01;DP=13;ExcessHet=0.0000;FS=0.000;MLEAC=1;MLEAF=0.500;MQ=60.00;MQRankSum=0.00;QD=7.82;ReadPosRankSum=1.59;SOR=0.495	GT:AD:DP:GQ:PL	0/1:7,6:13:99:109,0,151	BF.7	no
22674	C	T	163406.1	PASS	AC=2;AF=1.00;AN=2;DP=3797;ExcessHet=0.0000;FS=0.000;MLEAC=2;MLEAF=1.00;MQ=60.00;QD=26.08;SOR=0.814	GT:AD:DP:GQ:PL	1/1:0,3665:3665:99:163420,11039,0	BF.7	yes
22679	T	C	131106.1	PASS	AC=2;AF=1.00;AN=2;BaseQRankSum=-1.908e+00;DP=3538;ExcessHet=0.0000;FS=3.329;MLEAC=2;MLEAF=1.00;MQ=60.00;MQRankSum=0.00;QD=33.85;ReadPosRankSum=0.305;SOR=0.698	GT:AD:DP:GQ:PL	1/1:34,3398:3432:99:131120,8977,0	BF.7	yes
22686	C	T	123199.1	PASS	AC=2;AF=1.00;AN=2;BaseQRankSum=0.114;DP=3375;ExcessHet=0.0000;FS=0.000;MLEAC=2;MLEAF=1.00;MQ=60.00;MQRankSum=0.00;QD=27.08;ReadPosRankSum=1.64;SOR=0.575	GT:AD:DP:GQ:PL	1/1:70,3202:3272:99:123213,6913,0	BF.7	yes
22688	A	G	120437.1	PASS	AC=2;AF=1.00;AN=2;BaseQRankSum=-5.330e-01;DP=3310;ExcessHet=0.0000;FS=0.000;MLEAC=2;MLEAF=1.00;MQ=60.00;M	GT:AD:DP:	1/1:31,3133:3226:99:1204	BF.7	yes

					QRankSum=0.00;QD=25.42;ReadPosRankSum=1.90;SOR=0.878	GQ:PL	51,8377,0		
22775	G	A	139948.1	PASS	AC=2;AF=1.00;AN=2;BaseQRankSum=0.887;DP=3287;ExcessHet=0.0000;FS=0.000;MLEAC=2;MLEAF=1.00;MQ=60.00;MQRankSum=0.00;QD=29.48;ReadPosRankSum=2.12;SOR=0.061	GT:AD:DP:GQ:PGT:PID:PL:PS	1|1:3,3172:3175:99:1|1:22775_G_A:139962,9482,0:22775	BF.7	yes
22786	A	C	167274.1	PASS	AC=2;AF=1.00;AN=2;DP=3775;ExcessHet=0.0000;FS=0.000;MLEAC=2;MLEAF=1.00;MQ=60.00;QD=29.48;SOR=0.726	GT:AD:DP:GQ:PL	1/1:0,3644:3645:99:167288,11345,0	BF.7	yes
22813	G	T	166245.1	PASS	AC=2;AF=1.00;AN=2;BaseQRankSum=2.96;DP=4042;ExcessHet=0.0000;FS=0.000;MLEAC=2;MLEAF=1.00;MQ=60.00;MQRankSum=0.00;QD=27.08;ReadPosRankSum=0.700;SOR=0.138	GT:AD:DP:GQ:PL	1/1:3,3959:3962:99:166259,11851,0	BF.7	yes
22882	T	G	198234.1	PASS	AC=2;AF=1.00;AN=2;BaseQRankSum=-8.110e-01;DP=4653;ExcessHet=0.0000;FS=0.000;MLEAC=2;MLEAF=1.00;MQ=60.00;MQRankSum=0.00;QD=33.63;ReadPosRankSum=1.21;SOR=0.275	GT:AD:DP:GQ:PL	1/1:1,4462:4465:99:198248,13651,0	BF.7	yes
22917	T	G	227579.1	PASS	AC=2;AF=1.00;AN=2;DP=5113;ExcessHet=0.0000;FS=0.000;MLEAC=2;MLEAF=1.00;MQ=60.00;QD=30.55;SOR=0.765	GT:AD:DP:GQ:PGT:PID:PL:PS	1|1:0,5104:5104:99:1|1:22917_T_G:227593,15363,0:22917	BF.7	yes

22992	G	A	225305.1	PASS	AC=2;AF=1.00;AN=2;DP=5085;ExcessHet=0.0000;FS=0.000;MLEAC=2;MLEAF=1.00;MQ=60.00;QD=25.42;SOR=1.024	GT:AD:DP:GQ:PGT:PID:PL:PS	1|1:0,5025:5025:99:1|1:22917_T_G:225319,15125,0:22917	BF.7	yes
22995	C	A	225325.1	PASS	AC=2;AF=1.00;AN=2;DP=5017;ExcessHet=0.0000;FS=0.000;MLEAC=2;MLEAF=1.00;MQ=60.00;QD=27.65;SOR=1.013	GT:AD:DP:GQ:PGT:PID:PL:PS	1|1:0,5017:5017:99:1|1:22917_T_G:225339,15101,0:22917	BF.7	yes
23013	A	C	196142.1	PASS	AC=2;AF=1.00;AN=2;DP=4360;ExcessHet=0.0000;FS=0.000;MLEAC=2;MLEAF=1.00;MQ=60.00;QD=29.48;SOR=0.996	GT:AD:DP:GQ:PGT:PID:PL:PS	1|1:0,4360:4360:99:1|1:22917_T_G:196156,13124,0:22917	BF.7	yes
23018	T	G	186904.1	PASS	AC=2;AF=1.00;AN=2;DP=4163;ExcessHet=0.0000;FS=0.000;MLEAC=2;MLEAF=1.00;MQ=60.00;QD=30.56;SOR=1.007	GT:AD:DP:GQ:PGT:PID:PL:PS	1|1:0,4155:4155:99:1|1:22917_T_G:186918,12507,0:22917	BF.7	yes
23055	A	G	136843.1	PASS	AC=2;AF=1.00;AN=2;DP=3044;ExcessHet=0.0000;FS=0.000;MLEAC=2;MLEAF=1.00;MQ=60.00;QD=34.30;SOR=0.763	GT:AD:DP:GQ:PL	1/1:0,3043:3043:99:136857,9160,0	BF.7	yes

23063	A	T	137215.1	PASS	AC=2;AF=1.00;AN=2;DP=3059;ExcessHet=0.0000;FS=0.000;MLEAC=2;MLEAF=1.00;MQ=60.00;QD=27.00;SOR=0.829	GT:AD:DP:GQ:PL	1/1:0,3059:3059:99:137229,9208,0	BF.7	yes
23075	T	C	137367.1	PASS	AC=2;AF=1.00;AN=2;DP=3175;ExcessHet=0.0000;FS=0.000;MLEAC=2;MLEAF=1.00;MQ=60.00;QD=30.82;SOR=0.852	GT:AD:DP:GQ:PL	1/1:0,3110:3110:99:137381,9360,0	BF.7	yes
23191	C	T	12586.06	PASS	AC=2;AF=1.00;AN=2;BaseQRankSum=0.946;DP=452;ExcessHet=0.0000;FS=0.000;MLEAC=2;MLEAF=1.00;MQ=60.00;MQRankSum=0.00;QD=28.74;ReadPosRankSum=2.08;SOR=0.521	GT:AD:DP:GQ:PL	1/1:3,435:438:99:12600,1230,0	BF.7	yes
23403	A	G	238939.1	PASS	AC=2;AF=1.00;AN=2;BaseQRankSum=-1.388e+00;DP=6196;ExcessHet=0.0000;FS=0.000;MLEAC=2;MLEAF=1.00;MQ=60.00;MQRankSum=-1.000e-03;QD=35.92;ReadPosRankSum=-2.370e-01;SOR=0.474	GT:AD:DP:GQ:PL	1/1:3,5904:5915:99:238953,17846,0	BF.7	yes
23439	C	T	5591.64	PASS	AC=1;AF=0.500;AN=2;BaseQRankSum=1.71;DP=461;ExcessHet=0.0000;FS=1.746;MLEAC=1;MLEAF=0.500;MQ=60.00;MQRankSum=0.00;QD=12.77;ReadPosRankSum=-8.730e-01;SOR=0.777	GT:AD:DP:GQ:PL	0/1:194,244:438:99:5599,0,4075	BF.7	yes
23525	C	T	246620.1	PASS	AC=2;AF=1.00;AN=2;BaseQRankSum=3.00;DP=6018;ExcessHet=0.0000;FS=0.000;MLEAC=2;MLEAF=1.00;MQ=60.00;MQRankSum=-1.000e-03;QD=29.63;ReadPosRankSum=-1.310e+00;SOR=0.461	GT:AD:DP:GQ:PL	1/1:1,5914:5915:99:246634,17791,0	BF.7	yes

23599	T	G	211523.1	PASS	AC=2;AF=1.00;AN=2;BaseQRankSum=-1.302e+00;DP=4795;ExcessHet=0.0000;FS=0.000;MLEAC=2;MLEAF=1.00;MQ=60.00;MQRankSum=0.104;QD=28.76;ReadPosRankSum=3.50;SOR=0.835	GT:AD:DP:GQ:PL	1/1:19,4759:4778:99:211537,13526,0	BF.7	yes
23604	C	A	202559.1	PASS	AC=2;AF=1.00;AN=2;DP=4628;ExcessHet=0.0000;FS=0.000;MLEAC=2;MLEAF=1.00;MQ=60.00;MQRankSum=0.00;QD=27.00;SOR=0.945	GT:AD:DP:GQ:PL	1/1:4,4509:4517:99:202573,13561,0	BF.7	yes
23854	C	A	180109.1	PASS	AC=2;AF=1.00;AN=2;BaseQRankSum=-6.060e-01;DP=5020;ExcessHet=0.0000;FS=0.000;MLEAC=2;MLEAF=1.00;MQ=60.00;MQRankSum=0.00;QD=29.94;ReadPosRankSum=-1.539e+00;SOR=0.342	GT:AD:DP:GQ:PL	1/1:1,4856:4857:99:180123,14568,0	BF.7	yes
23948	G	T	185316.1	PASS	AC=2;AF=1.00;AN=2;BaseQRankSum=2.49;DP=5028;ExcessHet=0.0000;FS=0.000;MLEAC=2;MLEAF=1.00;MQ=60.00;MQRankSum=0.260;QD=31.54;ReadPosRankSum=3.32;SOR=0.476	GT:AD:DP:GQ:PL	1/1:4,4892:4898:99:185330,14610,0	BF.7	yes
24106	C	T	406.64	PASS	AC=1;AF=0.500;AN=2;BaseQRankSum=0.242;DP=68;ExcessHet=0.0000;FS=0.000;MLEAC=1;MLEAF=0.500;MQ=60.00;MQRankSum=0.00;QD=6.16;ReadPosRankSum=0.307;SOR=0.770	GT:AD:DP:GQ:PL	0/1:44,22:66:99:414,0,1009	BF.7	yes
24150	C	G	35.48	PASS	AC=2;AF=1.00;AN=2;DP=1;ExcessHet=0.0000;FS=0.000;MLEAC=1;MLEAF=0.500;MQ=60.00;QD=28.93;SOR=1.609	GT:AD:DP:GQ:PGT:PID:PL:PS	1|1:0,1:1:3:1|1:24150_C_G:45,3,0:24150	BF.7	no

24151	T	A	35.48	PASS	AC=2;AF=1.00;AN=2;DP=1;ExcessHet=0.0000;FS=0.000;MLEAC=1;MLEAF=0.500;MQ=60.00;QD=28.80;SOR=1.609	GT:AD:DP:GQ:PGT:PID:PL:PS	1|1:0,1:1:3:1|1:24150_C_G:45,3,0:24150	BF.7	no
24381	C	T	371.06	PASS	AC=2;AF=1.00;AN=2;DP=13;ExcessHet=0.0000;FS=0.000;MLEAC=2;MLEAF=1.00;MQ=60.00;QD=30.92;SOR=1.022	GT:AD:DP:GQ:PL	1/1:0,12:12:36:385,36,0	BF.7	no
24424	A	T	247851.1	PASS	AC=2;AF=1.00;AN=2;BaseQRankSum=-1.301e+00;DP=6018;ExcessHet=0.0000;FS=0.000;MLEAC=2;MLEAF=1.00;MQ=60.00;MQRankSum=0.00;QD=31.54;ReadPosRankSum=-2.670e-01;SOR=0.033	GT:AD:DP:GQ:PL	1/1:12,5741:5766:99:247865,17076,0	BF.7	yes
24469	T	A	240009.1	PASS	AC=2;AF=1.00;AN=2;DP=5834;ExcessHet=0.0000;FS=0.000;MLEAC=2;MLEAF=1.00;MQ=60.00;QD=28.86;SOR=0.860	GT:AD:DP:GQ:PL	1/1:0,5728:5752:99:240023,17209,0	BF.7	yes
24720	C	T	52.64	PASS	AC=1;AF=0.500;AN=2;BaseQRankSum=-2.530e-01;DP=10;ExcessHet=0.0000;FS=0.000;MLEAC=1;MLEAF=0.500;MQ=60.00;MQRankSum=0.00;QD=5.26;ReadPosRankSum=-1.593e+00;SOR=0.693	GT:AD:DP:GQ:PGT:PID:PL:PS	0|1:8,2:10:60:0|1:24720_C_T:60,0,330:24720	BF.7	no
24731	C	T	35.64	PASS	AC=1;AF=0.500;AN=2;BaseQRankSum=1.29;DP=16;ExcessHet=0.0000;FS=0.000;MLEAC=1;MLEAF=0.500;MQ=60.00;MQRankSum=0.	GT:AD:DP:GQ:PL	0/1:13,3:16:43:43,0,291	BF.7	no

					00;QD=2.23;ReadPosRankSum=0.405;SOR=1.002				
25000	C	T	187992.1	PASS	AC=2;AF=1.00;AN=2;BaseQRankSum=0.936;DP=5288;ExcessHet=0.0000;FS=0.000;MLEAC=2;MLEAF=1.00;MQ=60.00;MQRankSum=0.00;QD=28.76;ReadPosRankSum=0.037;SOR=0.437	GT:AD:DP:GQ:PL	1/1:1,5136:5137:99:188006,15391,0	BF.7	yes
25290	G	T	182737.1	PASS	AC=2;AF=1.00;AN=2;BaseQRankSum=3.81;DP=5583;ExcessHet=0.0000;FS=0.000;MLEAC=2;MLEAF=1.00;MQ=60.00;MQRankSum=-3.800e-02;QD=33.91;ReadPosRankSum=6.12;SOR=0.819	GT:AD:DP:GQ:PL	1/1:298,5091:5392:99:182751,5430,0	BF.7	yes
25333	T	A	183386.1	PASS	AC=2;AF=1.00;AN=2;DP=4548;ExcessHet=0.0000;FS=0.000;MLEAC=2;MLEAF=1.00;MQ=60.00;MQRankSum=-2.880e-01;QD=31.54;SOR=0.430	GT:AD:DP:GQ:PGT:PID:PL:PS	1|1:1,4487:4488:99:1|1:25290_G_T:183400,13447,0:25290	BF.7	no
25584	C	T	233719.1	PASS	AC=2;AF=1.00;AN=2;BaseQRankSum=-6.410e-01;DP=6477;ExcessHet=0.0000;FS=0.000;MLEAC=2;MLEAF=1.00;MQ=60.00;MQRankSum=-1.000e-03;QD=29.41;ReadPosRankSum=0.811;SOR=0.354	GT:AD:DP:GQ:PL	1/1:1,5925:5951:99:233733,17886,0	BF.7	yes
26060	C	T	227549.1	PASS	AC=2;AF=1.00;AN=2;BaseQRankSum=2.68;DP=6003;ExcessHet=0.0000;FS=0.000;MLEAC=2;MLEAF=1.00;MQ=60.00;MQRankSum=0.382;QD=31.11;ReadPosRankSum=1.66;SOR=0.461	GT:AD:DP:GQ:PL	1/1:5,5743:5754:99:227563,17037,0	BF.7	yes
26270	C	T	237507.1	PASS	AC=2;AF=1.00;AN=2;BaseQRankSum=1.85;DP=6199;ExcessHet=0.0000;FS=0.000;MLEAC=2;MLEAF=1.00;	GT:AD:DP:	1/1:2,4378:4410:99:23752	BF.7	yes

					MQ=60.00;MQRankSum=-1.660e-01;QD=34.66;ReadPosRankSum=-3.670e-01;SOR=0.231	GQ:PL	1,17917,0		
26529	G	A	247802.1	PASS	AC=2;AF=1.00;AN=2;BaseQRankSum=0.413;DP=5928;ExcessHet=0.0000;FS=0.000;MLEAC=2;MLEAF=1.00;MQ=60.00;MQRankSum=-4.970e-01;QD=29.40;ReadPosRankSum=1.21;SOR=0.110	GT:AD:DP:GQ:PL	1/1:3,5771:5776:99:247816,17270,0	BF.7	yes
26577	C	G	253342.1	PASS	AC=2;AF=1.00;AN=2;DP=5987;ExcessHet=0.0000;FS=0.000;MLEAC=2;MLEAF=1.00;MQ=60.00;MQRankSum=-6.360e-01;QD=36.76;SOR=0.187	GT:AD:DP:GQ:PL	1/1:2,5906:5908:99:253356,17733,0	BF.7	yes
26620	G	A	35.48	PASS	AC=2;AF=1.00;AN=2;DP=1;ExcessHet=0.0000;FS=0.000;MLEAC=1;MLEAF=0.500;MQ=60.00;QD=28.07;SOR=1.609	GT:AD:DP:GQ:PGT:PID:PL:PS	1|1:0,1:1:3:1|1:26619_T_TAATAA:45,3,0:26619	BF.7	no
26622	C	A	35.48	PASS	AC=2;AF=1.00;AN=2;DP=1;ExcessHet=0.0000;FS=0.000;MLEAC=1;MLEAF=0.500;MQ=60.00;QD=29.04;SOR=1.609	GT:AD:DP:GQ:PGT:PID:PL:PS	1|1:0,1:1:3:1|1:26619_T_TAATAA:45,3,0:26619	BF.7	no
26709	G	A	226413.1	PASS	AC=2;AF=1.00;AN=2;BaseQRankSum=-4.450e-01;DP=5950;ExcessHet=0.0000;FS=0.000;MLEAC=2;MLEAF=1.00;MQ=60.00;MQRankSum=-2.500e-	GT:AD:DP:GQ:PL	1/1:1,5810:5813:99:226427,17409,0	BF.7	yes

<table>
<tr><td></td><td></td><td></td><td></td><td></td><td>02;QD=36.76;ReadPosRankSum=1.50;SOR=0.615</td><td></td><td></td><td></td><td></td></tr>
<tr><td>26776</td><td>C</td><td>T</td><td>9328.64</td><td>PASS</td><td>AC=1;AF=0.500;AN=2;BaseQRankSum=0.419;DP=735;ExcessHet=0.0000;FS=1.114;MLEAC=1;MLEAF=0.500;MQ=60.00;MQRankSum=0.00;QD=12.88;ReadPosRankSum=-9.680e-01;SOR=0.597</td><td>GT:AD:DP:GQ:PL</td><td>0/1:311,413:724:99:9336,0,6321</td><td>BF.7</td><td>no</td></tr>
<tr><td>26897</td><td>T</td><td>C</td><td>119587.1</td><td>PASS</td><td>AC=2;AF=1.00;AN=2;BaseQRankSum=2.91;DP=4205;ExcessHet=0.0000;FS=0.000;MLEAC=2;MLEAF=1.00;MQ=60.00;MQRankSum=0.00;QD=29.48;ReadPosRankSum=1.28;SOR=0.644</td><td>GT:AD:DP:GQ:PL</td><td>1/1:331,3725:4056:99:119601,1053,0</td><td>BF.7</td><td>no</td></tr>
<tr><td>27038</td><td>A</td><td>G</td><td>211100.1</td><td>PASS</td><td>AC=2;AF=1.00;AN=2;BaseQRankSum=1.54;DP=6130;ExcessHet=0.0000;FS=0.000;MLEAC=2;MLEAF=1.00;MQ=60.00;MQRankSum=-5.300e-02;QD=21.53;ReadPosRankSum=7.27;SOR=0.562</td><td>GT:AD:DP:GQ:PL</td><td>1/1:243,5534:5820:99:211114,7560,0</td><td>BF.7</td><td>yes</td></tr>
<tr><td>27335</td><td>C</td><td>T</td><td>93.64</td><td>PASS</td><td>AC=1;AF=0.500;AN=2;BaseQRankSum=0.396;DP=13;ExcessHet=0.0000;FS=0.000;MLEAC=1;MLEAF=0.500;MQ=60.00;MQRankSum=0.00;QD=7.20;ReadPosRankSum=0.111;SOR=0.527</td><td>GT:AD:DP:GQ:PGT:PID:PL:PS</td><td>0|1:9,4:13:99:0|1:27335_C_T:101,0,290:27335</td><td>BF.7</td><td>no</td></tr>
<tr><td>27344</td><td>A</td><td>T</td><td>58.64</td><td>PASS</td><td>AC=1;AF=0.500;AN=2;BaseQRankSum=0.00;DP=8;ExcessHet=0.0000;FS=0.000;MLEAC=1;MLEAF=0.500;MQ=60.00;MQRankSum=0.00;QD=7.33;ReadPosRankSum=-6.190e-01;SOR=0.693</td><td>GT:AD:DP:GQ:PGT:PID:PL:PS</td><td>0|1:6,2:8:66:0|1:27335_C_T:66,0,246:27335</td><td>BF.7</td><td>no</td></tr>
</table>

27345	A	T	58.64	PASS	AC=1;AF=0.500;AN=2;BaseQRankSum=0.00;DP=8;ExcessHet=0.0000;FS=0.000;MLEAC=1;MLEAF=0.500;MQ=60.00;MQRankSum=0.00;QD=7.33;ReadPosRankSum=-6.190e-01;SOR=0.693	GT:AD:DP:GQ:PGT:PID:PL:PS	0|1:6,2:8:66:0|1:27335_C_T:66,0,246:27335	BF.7	no
27354	A	G	52.64	PASS	AC=1;AF=0.500;AN=2;BaseQRankSum=0.253;DP=10;ExcessHet=0.0000;FS=0.000;MLEAC=1;MLEAF=0.500;MQ=60.00;MQRankSum=0.00;QD=5.26;ReadPosRankSum=-2.287e+00;SOR=0.693	GT:AD:DP:GQ:PGT:PID:PL:PS	0|1:8,2:10:60:0|1:27335_C_T:60,0,249:27335	BF.7	no
27438	T	C	8012.06	PASS	AC=2;AF=1.00;AN=2;BaseQRankSum=1.09;DP=308;ExcessHet=0.0000;FS=0.000;MLEAC=2;MLEAF=1.00;MQ=60.00;MQRankSum=0.00;QD=26.89;ReadPosRankSum=-2.600e-02;SOR=0.721	GT:AD:DP:GQ:PL	1/1:6,292:298:99:8026,744,0	BF.7	no
27461	G	A	67.64	PASS	AC=1;AF=0.500;AN=2;BaseQRankSum=-1.242e+00;DP=8;ExcessHet=0.0000;FS=0.000;MLEAC=1;MLEAF=0.500;MQ=60.00;MQRankSum=0.00;QD=8.45;ReadPosRankSum=0.00;SOR=0.693	GT:AD:DP:GQ:PL	0/1:4,4:8:75:75,0,109	BF.7	no
27532	C	T	209715.1	PASS	AC=2;AF=1.00;AN=2;BaseQRankSum=-5.430e-01;DP=5830;ExcessHet=0.0000;FS=0.000;MLEAC=2;MLEAF=1.00;MQ=60.00;MQRankSum=0.055;QD=34.87;ReadPosRankSum=1.98;SOR=0.628	GT:AD:DP:GQ:PL	1/1:198,5436:5634:99:209729,9293,0	BF.7	yes

<table>
<tr><td>27634</td><td>T</td><td>A</td><td>61.64</td><td>PASS</td><td>AC=1;AF=0.500;AN=2;BaseQRankSum=-2.720e-01;DP=15;ExcessHet=0.0000;FS=2.796;MLEAC=1;MLEAF=0.500;MQ=60.00;MQRankSum=0.00;QD=4.11;ReadPosRankSum=-6.280e-01;SOR=0.939</td><td>GT:AD:DP:GQ:PGT:PID:PL:PS</td><td>0|1:12,3:15:69:0|1:27634_T_A:69,0,414:27634</td><td>BF.7</td><td>no</td></tr>
<tr><td>27807</td><td>C</td><td>T</td><td>245181.1</td><td>PASS</td><td>AC=2;AF=1.00;AN=2;DP=6083;ExcessHet=0.0000;FS=0.000;MLEAC=2;MLEAF=1.00;MQ=60.00;QD=25.57;SOR=0.764</td><td>GT:AD:DP:GQ:PL</td><td>1/1:0,4387:4402:99:245195,17918,0</td><td>BF.7</td><td>yes</td></tr>
<tr><td>27889</td><td>C</td><td>T</td><td>240261.1</td><td>PASS</td><td>AC=2;AF=1.00;AN=2;BaseQRankSum=2.41;DP=5971;ExcessHet=0.0000;FS=0.000;MLEAC=2;MLEAF=1.00;MQ=60.00;MQRankSum=-5.010e-01;QD=33.11;ReadPosRankSum=2.69;SOR=0.592</td><td>GT:AD:DP:GQ:PL</td><td>1/1:3,5824:5827:99:240275,17435,0</td><td>BF.7</td><td>yes</td></tr>
<tr><td>28093</td><td>C</td><td>T</td><td>207718.1</td><td>PASS</td><td>AC=2;AF=1.00;AN=2;BaseQRankSum=-1.340e+00;DP=5628;ExcessHet=0.0000;FS=0.000;MLEAC=2;MLEAF=1.00;MQ=60.00;MQRankSum=0.00;QD=27.86;ReadPosRankSum=1.63;SOR=0.453</td><td>GT:AD:DP:GQ:PL</td><td>1/1:1,5417:5423:99:207732,16261,0</td><td>BF.7</td><td>no</td></tr>
<tr><td>28271</td><td>A</td><td>T</td><td>254280.1</td><td>PASS</td><td>AC=2;AF=1.00;AN=2;DP=6024;ExcessHet=0.0000;FS=0.000;MLEAC=2;MLEAF=1.00;MQ=60.00;QD=34.17;SOR=0.794</td><td>GT:AD:DP:GQ:PGT:PID:PL:PS</td><td>1|1:0,5904:5904:99:1|1:28271_A_T:254294,17762,0:28271</td><td>BF.7</td><td>yes</td></tr>
<tr><td>28311</td><td>C</td><td>T</td><td>259885.1</td><td>PASS</td><td>AC=2;AF=1.00;AN=2;DP=5867;ExcessHet=0.0000;FS=0.000;MLEAC=2;MLEAF</td><td>GT:AD:DP:</td><td>1/1:2,4789:4791:99:25989</td><td>BF.7</td><td>yes</td></tr>
</table>

					=1.00;MQ=60.00;QD=28.60;SOR=0.969	GQ:PL	9,17468,0		
28330	A	G	2498 89.1	PASS	AC=2;AF=1.00;AN=2;DP=5589;ExcessHet=0.0000;FS=0.000;MLEAC=2;MLEAF=1.00;MQ=60.00;QD=25.57;SOR=0.933	GT:AD:DP:GQ:PL	1/1:0,5563:5578:99:249903,16746,0	BF.7	yes
28509	G	T	1098 8.06	PASS	AC=2;AF=1.00;AN=2;BaseQRankSum=-8.050e-01;DP=442;ExcessHet=0.0000;FS=0.000;MLEAC=2;MLEAF=1.00;MQ=60.00;MQRankSum=0.00;QD=26.87;ReadPosRankSum=-2.384e+00;SOR=0.368	GT:AD:DP:GQ:PL	1/1:3,406:409:99:11002,1135,0	BF.7	no
28690	G	C	3300.64	PASS	AC=1;AF=0.500;AN=2;BaseQRankSum=-4.700e-02;DP=355;ExcessHet=0.0000;FS=1.266;MLEAC=1;MLEAF=0.500;MQ=60.00;MQRankSum=0.00;QD=9.48;ReadPosRankSum=0.050;SOR=0.749	GT:AD:DP:GQ:PL	0/1:193,155:348:99:3308,0,4414	BF.7	no
28881	G	A	2264 07.1	PASS	AC=2;AF=1.00;AN=2;BaseQRankSum=-1.509e+00;DP=5213;ExcessHet=0.0000;FS=0.000;MLEAC=2;MLEAF=1.00;MQ=60.00;MQRankSum=0.00;QD=33.11;ReadPosRankSum=-1.504e+00;SOR=0.074	GT:AD:DP:GQ:PL	1/1:21,5048:5078:99:226421,14460,0	BF.7	yes
28882	G	A	2023 24.1	PASS	AC=2;AF=1.00;AN=2;BaseQRankSum=-6.430e-01;DP=4711;ExcessHet=0.0000;FS=0.000;MLEAC=2;MLEAF=1.00;MQ=60.00;MQRankSum=0.00;QD=30.36;ReadPosRankSum=1.85;SOR=0.749	GT:AD:DP:GQ:PL	1/1:4,3966:3992:99:202338,12818,0	BF.7	yes
28883	G	C	1957 93.1	PASS	AC=2;AF=1.00;AN=2;BaseQRankSum=-6.520e-01;DP=4492;ExcessHet=0.0000;FS=0.000;MLEAC=2;MLEAF=1.00;MQ=60.00;MQRankSum=0.168;QD=29.5	GT:AD:DP:GQ:PL	1/1:2,4379:4410:99:196576,12830,0	BF.7	yes

					3;ReadPosRankSum=1.71;SOR=0.169				
29090	G	T	19295.06	PASS	AC=2;AF=1.00;AN=2;BaseQRankSum=-1.073e+00;DP=708;ExcessHet=0.0000;FS=0.000;MLEAC=2;MLEAF=1.00;MQ=60.00;MQRankSum=0.00;QD=28.50;ReadPosRankSum=-1.670e+00;SOR=0.728	GT:AD:DP:GQ:PL	1/1:7,670:677:99:19309,1806,0	BF.7	no
29510	A	C	204213.1	PASS	AC=2;AF=1.00;AN=2;BaseQRankSum=3.20;DP=5586;ExcessHet=0.0000;FS=0.000;MLEAC=2;MLEAF=1.00;MQ=60.00;MQRankSum=-3.490e-01;QD=31.67;ReadPosRankSum=1.61;SOR=1.269	GT:AD:DP:GQ:PL	1/1:4,5300:5304:99:204227,15847,0	BF.7	yes
29518	C	T	152.64	PASS	AC=1;AF=0.500;AN=2;BaseQRankSum=1.08;DP=74;ExcessHet=0.0000;FS=0.000;MLEAC=1;MLEAF=0.500;MQ=60.00;MQRankSum=0.00;QD=2.24;ReadPosRankSum=-2.756e+00;SOR=0.854	GT:AD:DP:GQ:PGT:PID:PL:PS	0|1:56,12:68:99:0|1:29510_A_C:160,0,1409:29510	BF.7	no
29614	C	T	105244.06	PASS	AC=2;AF=1.00;AN=2;DP=391;ExcessHet=0.0000;FS=0.000;MLEAC=2;MLEAF=1.00;MQ=60.00;QD=27.48;SOR=0.981	GT:AD:DP:GQ:PL	1/1:0,383:383:99:10538,1148,0	BF.7	yes
29632	C	T	192356.1	PASS	AC=2;AF=1.00;AN=2;BaseQRankSum=4.79;DP=5364;ExcessHet=0.0000;FS=0.000;MLEAC=2;MLEAF=1.00;MQ=60.00;MQRankSum=-1.550e-01;QD=29.53;ReadPosRankSum=0.756;SOR=1.414	GT:AD:DP:GQ:PL	1/1:16,5104:5120:99:192370,15281,0	BF.7	yes
29666	C	T	5269.06	PASS	AC=2;AF=1.00;AN=2;BaseQRankSum=0.671;DP=196;ExcessHet=0.0000;FS=0.000;MLEAC=2;MLEAF=1.00;	GT:AD:DP:	1/1:2,188:190:99:5	BF.7	yes

					MQ=60.00;MQRankSum=0.00;QD=27.73;ReadPosRankSum=2.34;SOR=0.901	GQ:PL	283,521,0		
29845	T	G	166.14	PASS	AC=2;AF=1.00;AN=2;DP=4;ExcessHet=0.0000;FS=0.000;MLEAC=2;MLEAF=1.00;MQ=60.00;QD=31.31;SOR=0.693	GT:AD:DP:GQ:PGT:PID:PL:PS	1|1:0,4:4:12:1|1:29845_T_G:180,12,0:29845	BF.7	no
29846	T	A	166.14	PASS	AC=2;AF=1.00;AN=2;DP=4;ExcessHet=0.0000;FS=0.000;MLEAC=2;MLEAF=1.00;MQ=60.00;QD=31.45;SOR=0.693	GT:AD:DP:GQ:PGT:PID:PL:PS	1|1:0,4:4:12:1|1:29845_T_G:180,12,0:29845	BF.7	no
29868	G	A	1002.06	PASS	AC=2;AF=1.00;AN=2;DP=41;ExcessHet=0.0000;FS=0.000;MLEAC=2;MLEAF=1.00;MQ=60.00;QD=28.63;SOR=1.148	GT:AD:DP:GQ:PL	1/1:0,35:35:99:1016,105,0	BF.7	yes

Table S4. The negative controls of the top 20 bacteria and fungi

NC1	NC2	NC3	NC4	Bacteria Species
13.7404580152672	5.46355457034815	18.2940165599125	4.04562083064343	*Pseudomonas(Pseudomonas)*
0	0	0	29.600719917053	*Capnocytophaga(Capnocytophaga)*
0	22.9430173425479	0.187470707701922	0.514505937359391	*Allorhizobium-Neorhizobium-Pararhizobium-Rhizobium(Allorhizobium-Neorhizobium-Pararhizobium-Rhizobium)*
0	22.8321815099752	0	0.060645186532856	*Brevundimonas(Brevundimonas)*
19.8473282442748	0.0260790194288695	1.49455814195699	0.12911555842479	*Kocuria(Kocuria)*
17.5572519083969	0	0	0	*Pseudoxanthomonas(Pseudoxanthomonas)*
0.763358778625954	4.36823575433564	1.32791751288861	7.19721423401217	*(Proteobacteria)(Proteobacteria)*
0	2.36015125831269	9.40998802270479	1.54938670109748	*Delftia(Delftia)* *aureus,Candida albicans*
3.05343511450382	2.11892032859565	4.95235119512576	0.843163722440675	*(Comamonadaceae)(Comamonadaceae)*
7.63358778625954	0.0521580388577389	0.609279800031245	0.34626445213921	*Corynebacterium(Corynebacterium)*
3.81679389312977	0.599817446863998	3.67650887882102	0.0469511121544692	*Chryseobacterium(Chryseobacterium)*
3.05343511450382	1.11487808058417	2.40587408217466	1.30680595496606	*(Bacteria) (Bacteria)*
0	1.2778719520146	0.624902359006405	4.10430972083651	*Enhydrobacter(Enhydrobacter)*
1.52671755725191	0.847568131438258	2.73394782065302	0.643621495784181	*Acinetobacter(Acinetobacter)*
3.05343511450382	0.541139653149042	2.12987554028016	0.0039125926795391	*(Sphingomonadaceae)(Sphingomonadaceae)*
0	0	0	5.51284308547059	*Capnocytophaga uncultured Capnocytophaga sp.*
0	0	0.281206061552882	5.19592307842792	*Fusobacterium (Fusobacterium)*
0	5.17668535663059	0.0989428735093475	0	*Caulobacter (Caulobacter)*
4.58015267175572	0	0.489506847888351	0	*Corynebacterium uncultured bacterium*
0	5.03977050462903	0	0	*(Caulobacteraceae)(Caulobacteraceae)*

				Fungi Species
85.71428571	54.46428571	87.06165337	83.40168357	*AglaucusCBS516.65*
0	15.47619048	8.12761109	7.446578891	*AmnigerUAMH3544*
14.28571429	9.821428571	2.709203697	3.345564429	*AmutatusUAMH3576*
0	8.779761905	0.050639321	1.057630045	*AsydowiiCBS593.65*
0	6.398809524	0.215217116	0.280595726	*BcinereaB05-10*
0	4.31547619	0.025319661	0.884955752	*CeuropaeaCBS101466*
0	0	0.746929991	1.273472912	*CparapsilosisCDC317*
0	0	0	1.208720052	*CqueenslandicumCBS280.77*
0	0	0.113938473	1.036045759	*EoligospermaCBS72588*
0	0.595238095	0.36713508	0	*MglobosaCBS7966*
0	0	0.582352196	0.06475286	*MrestrictaKCTC27527*
0	0.148809524	0	0	*PjiroveciiSE8*
0	0	0	0	*PrubensWisconsin54-1255*
0	0	0	0	*Smacrosporak-hell*
0	0	0	0	*YlipolyticaCLIB122*
0	0	0	0	*YlipolyticaCLIB89W29*

Table S5. Escherichia coli and Aspergillus in bronchoalveolar lavage fluid detected by RNA-seq, confirmed by sputum culture

Sample	Clinical outcomes	RNA-seq	Sputum culture
s26	death	*Escherichia coli*，*Pseudomonas*，*Neisseria*	*Escherichia coli*
s2	death	*Escherichia coli*，*Streptococcus pneumoniae*，*Granulicatella*	*Escherichia coli*
s28	death	*Escherichia coli*，*Gemella*，*Klebsiella*	*Escherichia coli*
ss21	survival	*Aspergillus*，*Corynebacterium*，*Neisseria*	*Aspergillus*
ss7	death	*Aspergillus*，*Catellicoccus*，*Aliterella*	*Aspergillus*

Table S6. Characteristics of the HAP/CAP

	Survival(n=20) Median (IQR)	Death(n=7) Median (IQR)	*P* value
Age, yr	73.50 (26.00)	71.00 (24.00)	0.69
Sex			1.00
Male，n (%)	16.00(75.00)	6.00(85.71)	
Female，n (%)	4.00(25.00)	1.00(14.29)	

HAP/CAP:Hospital/Community Acquired Pneumonia,IQR=interquartile range, *P* values were evulated by Mann-Whitney test or Chi-square test ,Two-tailed degree of significance between groups was set at $P < 0.05$.

Table S7. Sputum culture and sequencing analysis identified pathogens in the HAP/CAP cohort

Sample	RNA-seq of Bronchoalveolar Lavage Fluid	Sputum culture
B20	*Proteus mirabilis*	*Proteus mirabilis*
B24	*C.albicans*	*Candida albicans*
B25	*C.albicans*	*Candida albicans*
B26	*Enterococcus*	*negative*
B27	*Acinetobacter baumannii*	*Acinetobacter baumannii*
B28	*C.albicans,Acinetobacter baumannii*	*Candida albicans,Acinetobacter baumannii*
B37	*Acinetobacter baumannii*	*Acinetobacter baumannii*
X13	*Staphylococcus aureus,Candida albicans*	*Staphylococcus aureus,Candida albicans*
X23	*Enterococcus,Candida*	*Enterococcus,Candida*
X5	*Acinetobacter baumannii,Klebsiella pneumoniae*	*Acinetobacter baumannii,Klebsiella pneumoniae*
X6	*Neisseria*	*Viridans Streptococci,Neisseria*
X8	*Pseudomonas aeruginosa*	*Pseudomonas aeruginosa*
X9	*Pseudomonas aeruginosa*	*Pseudomonas aeruginosa*
X3	*Acinetobacter baumannii*	*Acinetobacter baumannii*
